# Challenges and Opportunities of Pretrained Machine Learning Interatomic Potentials in Heterogeneous Catalysis

**DOI:** 10.1021/acscatal.5c08945

**Published:** 2026-02-18

**Authors:** Oliver Loveday, Kamila Kaźmierczak, Núria López

**Affiliations:** † 202569 Institute of Chemical Research of Catalonia (ICIQ-CERCA), The Barcelona Institute of Science and Technology, Av. Països Catalans 16, Tarragona 43007, Spain; ‡ Department of Physical and Inorganic Chemistry, Universitat Rovira i Virgili, Campus Sescelades, N4 Block, C. Marcellí Domingo 1, Tarragona 43007, Spain; § 133614 TotalEnergies, TotalEnergies One Tech Belgium, Zone Industrielle C, Feluy 7181, Belgium

**Keywords:** machine learning, MLIPs, foundational models, heterogeneous catalysis, RWGS

## Abstract

The design of catalysts gets its fundamental rationale from accurate and efficient modeling of reactivity on surfaces and materials. To reach this detailed atomistic understanding, density functional theory (DFT) has been the key computational technique. However, the emergence of machine learning interatomic potentials (MLIPs) marks a significant paradigm shift, offering the potential to match DFT accuracy at a drastically reduced computational cost. This perspective provides an overview of state-of-the-art MLIPs for heterogeneous catalysis as “out-of-the-box” tools. We summarize the different families of MLIPs and their training processes and then apply these pretrained models to heterogeneous catalysis problems. Furthermore, we critically address the challenges of model transferability and integration in unified frameworks, underscoring the necessity for standardized protocols to benchmark performance across different architectures. Finally, we assess the capacity of pretrained models to democratize computational catalysis, highlighting the specific hurdles that remain in achieving reliable, predictive power for widespread use.

## Graphical abstract

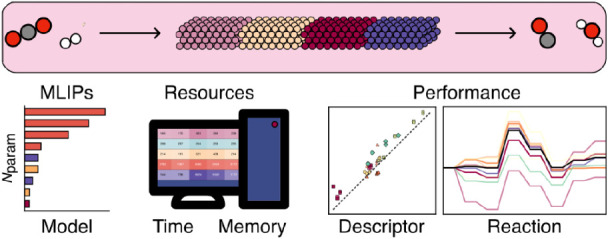

## Introduction

Heterogeneous catalysts are fundamental in the chemical industry, enabling sustainable and energy-efficient pathways for a wide range of reactions.^1,2^ The performance of a catalyst is dictated by the interaction of the environments and the chemical transformations occurring at specific sites under external stimuli in photo-, electro-, and thermal catalysis. Developing new and optimizing existing catalysts benefits from the precise understanding of these surface reaction mechanisms.^3^ Since the 1990s, density functional theory (DFT) has been the workhorse to generate this knowledge, offering key atomic-level insights into surface structures, adsorbate–surface interactions, and reaction pathways.^4^ DFT methodologies have been centered on highly idealized systems, such as periodic, clean metal surfaces, and have been key in establishing conceptual advances such as fundamental reactivity descriptors, linear scaling relationships, and volcano plots.^1^ These descriptors define modern catalysis research via the identification of activity trends,^5^ enabling rational catalyst design. Since DFT methodologies have become more robust, the differences between different implementations have become small, and the technology has been considered very mature and completely transferable to different types of materials.^6^ In parallel, the computational capabilities have increased enormously over the years, and databases with completely computationally produced data sets are now referenced just as handbooks were for other types of data.^7^

Focusing on mechanistic studies, reactions on surfaces are difficult to characterize, as they involve complex interfaces between gas or liquid-phase species and solid materials. This challenge is amplified by the fact that defining a realistic catalytic system is inherently complex. Heterogeneous catalysts are typically multicomponent and multiphasic, often exhibiting specific interactions with a support. Even in the simplest scenarios, active metal nanoparticles display diverse crystallographic orientations and dynamic effects. Consequently, the search for transition states connecting reactants and products relies on expensive, long, and tedious algorithms that require significant manual intervention.^8^ When these factors are coupled with lateral interactions, solvent effects, and the vast configurational space associated with larger molecules, exploration of full reaction networks becomes cumbersome. Ultimately, the practical application of DFT in this domain extends far beyond a static materials problem.

Constrained by this persistent trade-off between accuracy, computational cost, and the need for exhaustive analysis of mechanistic aspects of catalytic systems, the necessity for methods lighter than DFT emerges. This necessity is effectively addressed by the emergence of machine learning interatomic potentials (MLIPs). Capitalizing on the robustness of DFT algorithms and the high volume of materials data previously described, ML techniques are now used to learn the interactions between different types of atoms.^9–12^ Now, MLIPs neural networks are fitted to structures, energies, and forces, learning the interactions between different types of chemical entities (atoms) in a way that resembles force fields. By doing so, MLIPs can deliver near-DFT accuracy at a fraction of the cost, achieving computational speed-ups on the order of 10^4^.^13,14^

The growing ecosystem of sophisticated MLIPs has promoted the development of large-scale computational databases (e.g., MPTraj^15^ Alexandria,^16–18^ OC20 ^19^) that retroactively enable the training of new foundational models capable of generalizing across vast chemical spaces.

However, the rapid development of the field has revealed a gap between model-centric benchmarks and real-world applications. Many models, even those described as “universal”, often require significant fine-tuning to achieve the necessary accuracy for a specific case study. Thus, to quantify the performance of MLIPs, the community has responded by developing a wide range of benchmarking platforms^20–24^ to systematically rank the performance of MLIPs for materials, though these often focus on specific physical property descriptors at the atomic level. Recently, emerging frameworks have become powerful tools for evaluating MLIPs on an application basis. These include MLIP Arena^23^ for general downstream applications, and CatBench^25^ or MLIPX^26^ for unified environments in surface reactivity. These frameworks employ multistep evaluations that assess metrics such as predicted adsorbate-surface geometries and adsorption energies.

While these benchmarking frameworks provide essential quantitative metrics, the current literature landscape has predominantly focused on the architectural evolution and training methodologies of system-specific potentials. Recent reviews have traced the development of MLIPs from local descriptors to equivariant message-passing networks^27,28^ or categorized models based on their ability to capture physical phenomena such as long-range interactions or charge transfers.^29^ Similarly, recent advances highlight the advantages of integrating global optimization algorithms to active training of MLIPs for the search of catalyst structures and potential active sites.^30^

In the present perspective, our approach diverges from a focus on methodology to a readiness focus. Rather than reviewing architectures or new training methodologies, we move beyond general benchmarks to assess the “out-of-the-box” readiness of foundation MLIPs to serve as surrogates for DFT in standard pipeline methodologies. To do so, a challenging reaction, the reverse water–gas shift (RWGS), crucial to balance the CO and H_2_ streams via the utilization of CO_2_ serves as our baseline stress test. By evaluating their performance on this real-case application, we aim to quantify their practical readiness for reactivity studies and highlight the requirements necessary to accelerate implementation. This strategy serves as a stress test designed to transition from theoretical exploration to operational viability, moving beyond direct predicted outcome evaluation to a quantitative analysis of efficiency, reliability, and scalability. By assessing performance against a real-world application, we aim to determine the current robustness of MLIPs when subjected to the complex demands of reactivity studies, highlighting their advantages and bottlenecks, whether in model performance, data quality, or resource allocation, to accelerate implementation.

## Results and Discussion

### Landscape of Machine Learning Interatomic Potentials: Models and Architectures

The computational landscape of machine learning interatomic potentials (MLIPs) has evolved into a diverse ecosystem driven by academic institutions, national laboratories, and major technology companies.^31^ To provide a structured overview of this rapidly expanding field, we categorize the leading model families based on their accessibility, architectural philosophy, and primary application domain in Table 1.

**Table 1:** Overview of Leading Machine Learning Interatomic Potential Families^a^

Family	Architecture	Application Domain	Key Data sets	License
MACE	E(3)-Equivariant MPNN	Universal Materials	MPTraj, OMat, MatPES	MIT/ASL
OCP	Equivariant Transformer	Heterogeneous Catalysis	OC20, OC22	MIT
ORB	Attention-Augmented GNS	Universal Materials	MPTraj, Alexandria, OMat24	Apache-2.0
SevenNet	Parallel-Optimized MPNN	Universal MD	MPTraj, OMat24	GNU GPL
CHGNet	Charge-Informed GNN	Battery/Magnetic Materials	MPTraj	BSD-3
M3GNet	MPNN	Universal Materials	MPTraj	BSD-3
PET-MAD	Transformer-based GNN	Universal Materials	MPTraj	BSD-3
ALIGNN	Atomistic Line GNN	Solid State Properties	JARVIS, OC20	MIT
Matlantis	Equivariant GNN	Universal Materials	Proprietary PFP	Proprietary
LASP	Global-MBNN	Universal Materials & Reactivity	LASP-Global, ZWMD, MPCD	Proprietary

a Models are categorized by their primary architecture, application domain , key datasets , and licensing availability . MPNN = Message Passing Neural Network, GNN = Graph Neural Network, GNS = Graph Network-Based Simulator, MBNN = Many-Body Neural Network.

A prevalent trend is the commitment to open-science principles. Families such as MACE,^32^ OCP,^33^ SevenNet, and ORB^34,35^ provide open-source code and pretrained checkpoints, significantly lowering the entry barrier for researchers. Conversely, the landscape includes tiered-access models in the field of material science like MatterSim^36^ which reserves advanced capabilities for commercial cloud platforms. While this may seem tangential to the field of heterogeneous catalysis, it can be seen as a potential path that future MLIPs may follow. Fully proprietary systems, like Matlantis,^37^ operate behind strict paywalls. This distinction among open-source, restricted-access, and closed-source models was a critical factor in selecting the tools for this study.

An analysis of the architectural designs reveals a convergence on E(3)-equivariance as a foundational principle.^38–41^ By incorporating 3D Euclidean symmetries (rotation, reflection, translation) directly into the network, these models preserve fundamental physics by design rather than attempting to learn them from data.^42^ Table S1 outlines the distinct architectural strategies used for implementations. These strategies include the following: (i) equivariant message passing neural networks (MPNNs)^43^ are the dominant paradigm, employed by models such as MACE^32,44,45^ which use a high-order approach. This architecture iteratively updates atomic representations by passing E(3)-equivariant messages. (ii) Local-descriptor E(3)-equivariant models, such as Allegro,^46^ avoid message passing to achieve increased parallel scalability. Instead, the model relies on strictly local, equivariant tensor representations. (iii) Graph neural networks (GNNs) with explicit physical input such as CHGNet have been built upon a GNN foundation but explicitly incorporate physical information, such as charge or magnetic moments, to enable a deeper predictive layer of physical properties. Similarly, (iv) the LASP framework^47^ implements a global many-body function-corrected neural network. This approach enhances neural networks by explicitly incorporating many-body terms (from two- to four-body functions) into the output layer, allowing the capture of complex potential energy surfaces and long-range interactions with high efficiency. (v) Attention-based graph neural network architectures, present in the Orb models,^34,35^ integrate attention mechanisms into a graph network simulator (GNS) to differentially weight the importance of atomic neighbors and interactions. The OrbMol potentials, trained on the Open Molecules 2025 database,^48^ show the flexibility of these architectures to the addition of physical properties such as charge and spin. Finally, (vi) E(3)-equivariant transformers, exemplified by models such as Equiformer,^33^ adapt the transformer architecture to atomic graphs. These models utilize equivariant self-attention mechanisms to effectively capture nonlocal interactions and higher-body order correlations while enforcing symmetry constraints.

A critical distinction in model architecture related to simulation stability is force conservation. Strictly conservative models such as MACE and SevenNet predict scalar potential energy surfaces (PES) and derive atomic forces through automatic differentiation $\left(F_{i}=-\nabla_{r_{i}}E\right)$ . This constraint ensures energy conservation during NVE molecular dynamics (MD) and ensures geometry optimization convergence. In contrast, architectures used in the Orb and the OCP models employ direct force predictions, where forces are output by a specific vector-valued head of the network. While direct prediction may often lead to lower force errors in static applications, it trades off thermodynamic consistency. This nonconservative behavior can lead to energy drifts during MD and geometry optimizations unless post hoc corrections are applied.

The different design goals and architectural philosophies are most clearly reflected in their scalability, represented by the total number of parameters in the pretrained models. The models listed in Table 2 occupy a wide spectrum of model size, spanning more than 3 orders of magnitude. On the compact, lightweight side, we have models such as CHGNet, reporting ∼400k trainable parameters.^49^ In the middle of the spectrum, we have models like M3GNet, reporting ∼1.1M parameters,^50^ the NequIP and Allegro pretrained models, with 9.6M and 9.7M parameters, respectively. MACE models, while using an unspecific nomenclature, are contained in this range, presenting a count of 3.8M parameters for the smallest model and 15.8M parameters for the largest one. On the largest side of the spectrum, we have models such as EquiformerV2 from the Open Catalyst Project initiative. The smallest model has a size of 31M parameters, while the largest model has ∼153M parameters.

**Table 2:** General Model Specifications for State-of-the-Art Machine Learning Interatomic Potentials

Family	Model Size (Parameters)	Elements Covered
MACE	small, medium, large	89
OCP	1.8M – 300M	89–95
CHGNet	∼400k	94
M3GNet	Not specified	94
SevenNet	840k–3.27M	≥89
PET-MAD	2.8M	94
ORB	∼25M	117
Matlantis	Not specified	55–72
ALIGNN	∼4M	5–118

Transparency in reporting model parameter counts is a significant issue in the MLIP field. As indicated in Table 2, “Model Size (parameters)”, some model families do not directly report the number of parameters in their publications or code repositories. For instance, the MACE family utilizes a qualitative nomenclature (e.g., small, medium, large) to classify the model scale. This labeling hinders the practical assessment of computational resource requirements and limits direct, quantitative comparisons with models outside their ecosystem. Consequently, determining the parameter count requires manual inspection by loading the model. In contrast, the OCP family provides a better standard by explicitly reporting parameter counts for their pretrained models. This level of transparency, while seemingly trivial, is a step toward the accessibility and reproducibility desired in the field and should be encouraged as a standard practice.

### Data Set Diversity, Interoperability, and Transferability

As highlighted in Table 1, the choice of training set fundamentally defines the domain of reliability of a model. Details regarding the application scope and the corresponding level of theory can be found in Tables S2–S3. Ultimately, the quality, diversity, and coverage of this underlying data are pivotal for the performance and transferability of any machine learning interatomic potential (MLIP). Model performance depends directly on training data that adequately cover compositional and configurational spaces relevant to the application. Without comprehensive data, MLIPs cannot faithfully reproduce the underlying physics of known systems and are prone to failure when extrapolating to unseen atomic environments. This often occurs during geometry relaxations or molecular dynamics simulations, where trajectories drift into poorly sampled regions of the potential energy surface.

Consequently, constructing robust training sets has evolved from a challenge of data collection, often a byproduct of high-throughput computing, to purposeful data engineering. The creation of large-scale data sets, summarized in Table 3, is a direct response to this need. Open resources such as MPTraj,^15^ Alexandria,^16–18^ the Open Catalyst 2020 and 2022,^19,51^ or the Open Materials 2024 ^52^ serve as enabling technologies for training, benchmarking, and model generalization. Conversely, proprietary data sets, such as the PFP database,^53^ provide competitive advantages but limit reproducibility and qualitative analysis by the broader community. While Table 3 highlights a diverse heterogeneity in sampling strategies, from static relaxation to molecular dynamics (MD) and nonequilibrium structures, a convergence is observable regarding the level of theory. PBE-based functionals remain the common denominator between data sets, balancing computational cost with accuracy for pretraining large-scale foundation models.

**Table 3:** Overview of Available Large-Scale Computational Chemistry Datasets for Molecules, Materials, and Surface Reactivity

Data set	Domain	Scale & Type	Method	Description
MPTraj^15^	Materials	1.6M Traj. (*E,F,S,μ*)	PBE/PBE + U	Static & relaxed trajectories from Materials Project.^7^
MatPES^56^	Materials	∼400k MD	PBE/r2SCAN	Snapshots from ∼300k MD simulations.
Alexandria,^16–18^	Materials	2.5M Relax.	PBE	Stable and metastable material structures.
JARVIS-DFT^57^	Materials	40k Relax. (1M properties)	Various^a^	Relaxations using meta-GGA, hybrid, and MBPT.
Open Materials 24 ^52^	Materials	118M Noneq (*E,F,S*)	PBE + U	Physically relevant nonequilibrium structures.
Open Catalyst 20 ^19^	Surfaces	1.3M Relax., MD, rattling	PBE	82 C,H,O,N adsorbates on 55 different composition surfaces.
Open Catalyst 22 ^51^	Surfaces	62k Relax.	PBE + U	9 adsorbates on 62k A_*x*_O_*y*_/A_*x*_B_*y*_O_*z*_ oxides.
Open Catalyst 25 ^58^	Surfaces	7.8M NVT-AIMD	RPBE + D3	Solid–liquid interfaces with explicit solvation.
CLAM^59^	Surfaces	1.3M AIMD, Relax.	PBE	Metals, clusters, and 2D materials including Noneq
LASP-Global^59^	Surfaces	+5M Relax.	SSW-NN	Global PES data including xeolites (ZWMD, ZAD) & metal–ligands (MPCD)

a vdW-DF-OptB88, TBmBJ, PBE0, HSE06, DMFT, G_0_W_0_. Labels: Relax.: Relaxations, Traj.: Trajectories, MD: Molecular Dynamics, Non-Eq.: Non-Equilibrium Structures, E: Energy, F: Forces, S: Stress, μ: Magnetic Properties.

Widespread reliance on a limited number of databases may restrict data diversity, while systematic errors or specific simulation protocols within these repositories could induce correlated biases across different models. Recent studies highlight that training on strategically generated data sets with random structure sampling can have a greater impact than the architectural complexity or parameter count of these MLIP models.^54,55^

Many leading models aim to be universal MLIPs (*u*MLIPs) applicable to a wide range of chemical systems.^55^ However, the community is also moving toward specialization. OCP models, for instance, are strongly focused on heterogeneous catalysis, a domain with unique challenges, such as adsorbate–surface interactions. Similarly, many DeepMD^60,61^ and ALIGNN^62^ models are purpose-trained for specific applications, such as semiconductors, battery cathodes, or metal–organic frameworks (MOFs). This trend suggests that, for achieving higher accuracies, specialized models trained on domain-specific data may present better performance. As an example of this shift toward specialization, the LASP^47^ framework has generated massive specialized databases via stochastic surface walking (SSW) global optimization such as the Zeolite Wulff Morphology Database (ZWMD), the Zeolite Acidity Database (ZAD), and the Metal Phosphine-Ligand Catalyst Database (MPCD). Universal models, on the other hand, by embracing a broader chemical space, inevitably trade off some domain-specific performance. This is a recognized compromise, and it is expected that by performing fine-tuning or transfer learning with relatively small subsets of data, these general models can adapt to a specific problem. Specialized MLIPs, on the other hand, may require full training and/or architecture modifications to perform in initially out-of-domain systems.

To address the need for specialized training data for MLIPs, initiatives such as Catalyst-Hub,^63^ the NOMAD toolkit,^64^ and OPTIMADE^65–67^ aim to establish interoperability and standardized APIs. Despite these advancements, the lack of homogeneity and standardization between different origin data sets might be an issue. Although many of these databases are centered on simulations of materials and molecules, for surface reactivity, the situation is different. The OC20 and OC22 data sets are the largest available for adsorption of molecules on metal surfaces and oxides. Regardless of the large number of simulations carried out for the generation of these databases (∼1.2 million DFT relaxations for OC20 and ∼62,000 relaxations for OC22), these only comprise the presence of small C, H, O, N-containing adsorbates on C_1–2_ backbone structures over a wide range of surfaces for OC20, and 9 adsorbates (C*, H*, O*, N*, OH*, OOH*, H_2_O*, CO*, and $O_{2}^{*}$ ) over 62k metal oxides for OC22. This small sample of adsorbates can become a trade-off for these databases. While the accuracy or proper prediction abroad of a larger range of materials and surfaces may be ensured, models trained in such databases may struggle when predicting reactivity for processes involving out-of-distribution species. Thus, fine-tuning is expected to be mandatory when applying models trained on these data sets to larger or more complex reactions. From the same initiative, the recently developed OC25 ^58^ provides more than 7 million data points spanning more than 1.5 million different solvent environments for surface reactivity. To address data diversity, the Catalytic Large Atomic Model (CLAM) data set^59^ offers a unified alternative with 1.3 million structures spanning metals, clusters, and 2D materials. By aligning parameters with OC22 and prioritizing nonequilibrium AIMD configurations, the data set explicitly targets the modeling of dynamic active sites, overcoming inconsistencies found in static-dominated data sets. Alternatively, databases such MatPES^56^ containing ∼400k structures, 10× smaller than the widely used databases, highlight that with lower amounts of balanced data, it is possible to achieve similar or better performance. Similarly, the FG-data set^68^ developed for GAME-Net^69^ and its rutile oxide extension for GAME-Net-Ox^70^ collectively contain ∼8k DFT relaxations of large molecules (up to 4 carbons) on metals and oxides.

These balanced approaches are designed to improve the prediction of properties for new, unseen molecules but are constrained to a smaller space of surfaces. Thus, finding a balanced representation of both adsorbates and surface materials is a relevant factor to consider when generating an ML-oriented data set. Alternatively, new promising strategies are starting to emerge, as seen in the development of active learning frameworks^71^ or data set generation approaches, as seen in REICO,^55^ where random-structure sampling strategies are used as training sets for MLIPs, showing good performance results.

### Accessibility and Availability of Open Data Sets

The commitment to advancing the field toward data-driven approaches is underpinned by the widespread adoption of open-data practices.^72–74^ Major data sets are increasingly hosted on user-friendly, high-availability platforms that ensure accessibility, transparency, and long-term preservation. MatPES,^56^ for instance, is available on HuggingFace and can be downloaded via its own Pythonpackage. MPTraj^15^ is hosted on Figshare, a dedicated repository for research data. The Open Catalyst Project (OC20 and OC22,^19,51^ ) provides its data in precomputed LMDB (Lightning Memory-Mapped Database) formats via direct download links in their tutorials, supplemented by open-source code on GitHub for data processing. The QM9 data set^75,76^ is available on its own website, Figshare, Kaggle) and is also integrated directly into machine learning libraries like TensorFlow^77^ as TensorFlowDatasets. This multiplatform approach ensures that researchers can access the data through various channels, from direct download to API-driven workflows.

Positively, the main available data sets are typically accompanied by detailed documentation. The MatPES data set provides a dedicated website (matpes.ai) and a publication outlining its quality-first philosophy and high-convergence static DFT methodology.^56^ The Open Catalyst Project has also set a high bar for transparency, with dedicated publications for both OC20 ^19^ and OC22 ^51^ that detail the motivation, data generation workflow, DFT settings, and benchmark tasks. The public availability of their data generation and model training code further enhances their reproducibility. Finally, the NIST JARVIS project^57^ also emphasizes transparency, adhering to FAIR (Findable, Accessible, Interoperable, and Reusable) data principles^78^ and providing extensive documentation through its website and associated publications.^57^

As for the long-term availability, this is bolstered by the use of persistent repositories and permissive licensing. Hosting on platforms like Figshare, Zenodo, Hugging Face, and ioChem-DB^79^ provides data sets with stable digital object identifiers (DOIs), ensuring that they remain findable and citable. The licensing of these main data sets is designed to facilitate widespread use. For instance, MPTraj and Open Catalyst initiatives can be found under the MIT license, and MatPES uses the BSD-3-Clause license. The Alexandria and OMat24 data sets are available under a Creative Commons CC-BY-4.0 license. This commitment to open licensing is crucial to removing barriers to both academic research and commercial application.

In conclusion, while the MLIP field is converging toward robust open-science practices, achieving homogeneity and standardization across these growing data resources remains a persistent challenge, for which incentives need to be put in place. Future progress depends not only on improving data availability but also on establishing unified standards that allow the decoupling of the model architecture from data set bias. Ultimately, the field must reconcile this need for consistency with the challenge of balancing broad, universal coverage against the high-fidelity, targeted data required for specialized domains like catalysis.

### Benchmarking on a Real-Case Application

#### Reverse Water–Gas Shift Reaction: A Case Study to Benchmark State-of-the-Art MLIPs

To assess the performance and evaluate the limitations of leading state-of-the-art machine learning interatomic potentials (MLIPs), we selected a 7-step reaction mechanism for the reverse water–gas shift (RWGS) reaction, shown in (eqs 1–8). This reaction is industrially significant for recycling CO_2_ using green H_2_, achieving balanced industrial feeds.^80^ Additionally, it serves as an ideal foundational benchmark because it features adsorbed intermediates common to many catalytic processes, such as CO*, O*, or OH*. This creates a computationally efficient baseline for the initial performance categorization. We posit that this system acts as a scalability filter, where excessive computational demand at this level implies that the model will present prohibitive bottlenecks when applied to complex networks, rendering it impractical without the use of high-performance computing resources.

1a $$R_{1}:,H_{2}\left(g\right)+2^{*}\to {2H}^{*}$$

$$R_{2}:,{\mathrm{CO}}_{2}\left(g\right)\;+\;*\to {{\mathrm{CO}}_{2}}^{*}$$ 1b

$$R_{3}:,{{\mathrm{CO}}_{2}}^{*}\;+\;*\to {\mathrm{CO}}^{*}\;+O^{*}$$ 1c

$$R_{4}:,O^{*}+H^{*}\to {\mathrm{OH}}^{*}+*$$ 1d

$$R_{5}:,{\mathrm{OH}}^{*}+H^{*}\to {{\mathrm{CO}}_{2}}^{*}+*$$ 1e

$$R_{6}:,{\mathrm{CO}}^{*}\to \mathrm{CO}\left(g\right)+*$$ 1f

$$R_{7}:,H_{2}O^{*}\to H_{2}O\left(g\right)+*$$ 1g

1h $$\mathrm{Global}\;R:,{\mathrm{CO}}_{2}\left(g\right)+H_{2}\left(g\right)\to \mathrm{CO}\left(g\right)+H_{2}O\left(g\right)$$

The Catalytic Automatic Reaction Evaluation (CARE) framework,^81^ a tool developed for fast screening and catalytic discovery, has been used to perform the MLIP benchmark and evaluate its integration into pipelines for fast screening and catalyst discovery in the field. The initial strategy involved generating a C_1_O_2_ CRN containing 38 intermediates and 62 reactions. Although this network is larger than that presented in eqs 1–8, it was chosen to ensure a broader and more robust data set for the benchmark analysis. Once the CRN was generated, energies and forces for all species were computed by using a selected set of MLIPs. A 2-fold benchmark has been performed by analyzing predicted geometric and thermodynamic properties of intermediate species and elementary reactions. To establish the ground truth for this assessment, density functional theory (DFT) at the PBE-D3 level was used.^80^ We acknowledge, as detailed in recent benchmarks,^82,83^ that simulations at the PBE level tend to overestimate chemisorption energies on transition metals compared to higher parametrized functionals such as RPBE or BEEF-vdW. To quantify this discrepancy, we performed a comparative benchmark using PBE, PBE-D3, RPBE, and BEEF-vdW functionals (see Note S1, Figures S6–S7). However, we maintain PBE-D3 as the primary reference since PBE is the standard baseline for generating large training data sets such as MPTraj,^49^ Alexandria,^16–18^ OC20,^19^ OC22,^51^ or CLAM^59^ which models evaluated here are trained on. By comparing the MLIP-predicted adsorption energies against the DFT reference, we can pinpoint inaccuracies related to chemistry-related properties.

The selected surfaces consisted of Ag, Au, Pd, and Ni (111), along with Ni(110). This selection encompasses a range of chemical properties defined by their electronic structure, which are expected to accurately transfer from the DFT training data to the MLIP models. For instance, Ag and Au are noble metals with filled *d*-bands, resulting in weak interactions with most adsorbates. On the other hand, Ni and Pd have unfilled, high-energy *d*-bands that are readily available, favoring the interaction with the adsorbates, according to the *d*-band model.^84^ This approach is designed to reveal the fundamental reasons for model limitations in a real-world application in computational heterogeneous catalysis, setting the stage for analyzing their implementation capabilities and accuracy in the following sections.

The set of MLIPs to benchmark was chosen following a selection of criteria designed to ensure both open accessibility and relevance to the target physical domains. A first selection criterion was based on licensing and availability of pretrained models. This initial screening led to discarding models available behind a hard or soft paywall or those that provided only the architecture. Thus, Matlantis was discarded due to the paywall entry barrier. The second criterion was the domain of the models (Table 1). Models trained or built around molecular systems or developed for very specific property predictions such as CHGNet, and M3GNet have not been considered.

The selected family of models to assess the goal of this perspective are MACE, OCP, ORB, SevenNet, and PET-MAD. These models are readily available and integrated as ASE^85^ calculators. Thus, developing and integrating interfaces for each model in a framework remain fairly straightforward task. Subsequently, the required packages per model, the output energy, and how it is referenced are the main considerations to be taken into account. Regarding the implementation of these models into a unified framework, we encountered incompatibilities across the required Python packages. This necessitated the generation of separate environments for each model, designated as care-model. An analysis of these environments identifies significant package conflicts that prevent consolidation into unified ecosystems. As detailed in Table S4, these incompatibilities span core ML frameworks and CUDA libraries, mainly due to the divergence in development timelines for each model, making a unified environment unfeasible.

The primary critical barrier stems from discrepancies in PyTorch versions and the associated CUDA toolkit, the latter being essential for GPU acceleration. Specifically, ORB models require PyTorch >2.6.0, whereas other models rely on version 2.4.0. This presents a major hurdle, as PyTorch 2.6.0 introduces breaking changes and requires different CUDA toolkit versions. Consequently, models trained on one version may fail to load on another. A further source of conflict is the e3nn package, which is a core dependency for models such as MACE and EquiformerV2. MACE utilizes version 0.4.4, while EquiformerV2 requires release 0.5.7. This mismatch in e3nn has historically posed a significant burden when integrating these models into broader pipelines. As of January 2026, the multienvironment approach remains the only viable solution for managing these complex dependencies. By isolating dependencies, we ensure that each module functions correctly, despite the conflicting requirements dictated by their respective development eras.

#### Performance Assessment and Benchmark of MLIPs

To assess the performance of the different models, we start by evaluating execution time and model size, followed by a validation of predicted geometries and energies against reference DFT data.^80^ Structural accuracy was assessed by directly comparing predicted adsorption heights with the reference data, while energetic accuracy was quantified via the calculated adsorption energies. Details on the definition of these parameters can be found in Notes S1–S2. Finally, the predicted energy profile for the RWGS mechanism was compared to the DFT benchmark to quantify the error propagation of these parameters.

Computational cost is a critical factor for practical MLIP deployment and scalability. We analyzed the execution time required for structural relaxations across four metal surfaces (Ag, Au, Ni, and Pd). As shown in Figure 1, all models demonstrate a substantial speed-up compared to conventional DFT, reducing time scales to hours at most to evaluate a full CRN. Notably, MLIPs require longer execution times for Ni and Pd surfaces compared to the noble metals (Ag and Au), likely reflecting the increased computational effort needed to capture the more complex chemistry of the former. Particularly, the OCP and SevenNet families are the most computationally intensive, with execution times ranging from 2,000 to 9,800 s on a 12 GB GPU. Specifically, SevenNet models required nearly 3 h (9,800 s) for the Ni surfaces.

**Figure 1:**
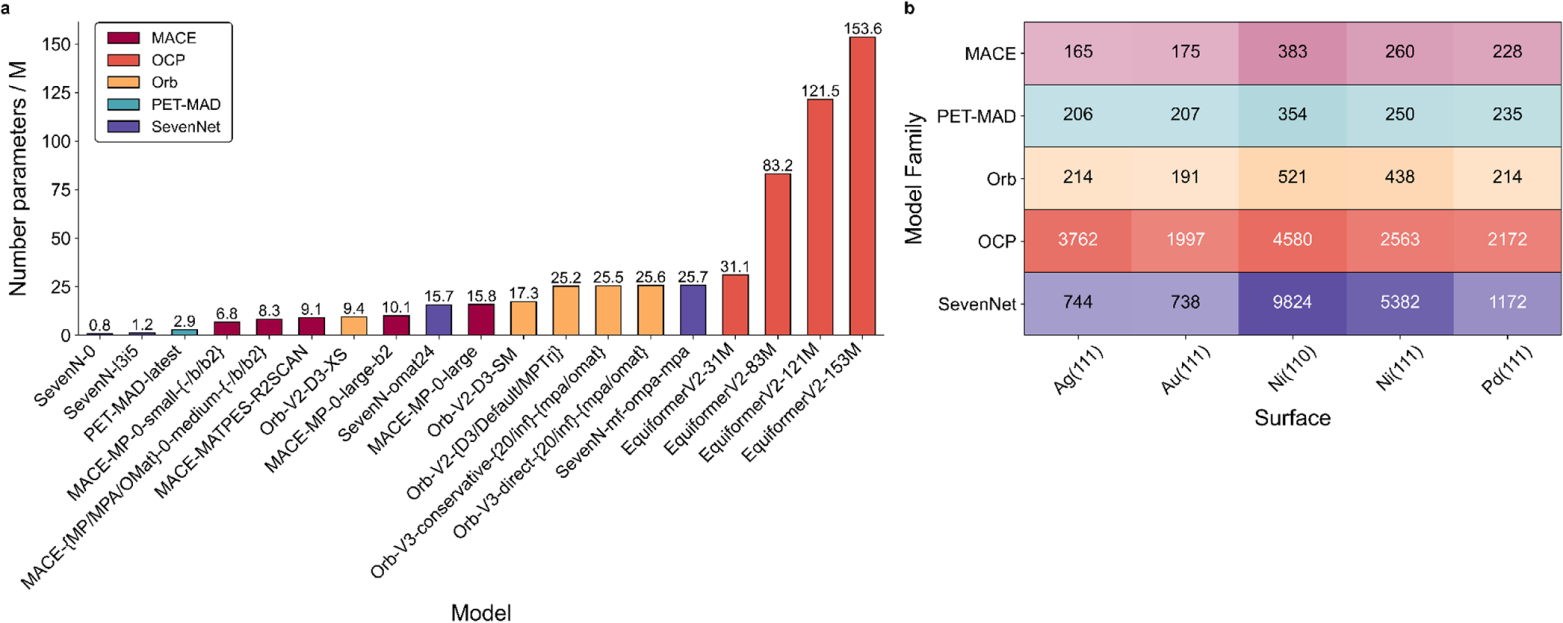
(a) Number of parameters (in millions) for each pretrained MLIP available. (b), Heatmap of average GPU (NVIDIA RTX A2000 12GB) execution times (in seconds) per model family and metal surface, aggregated over the energy evaluation of 38 intermediates and 62 reactions.

For pipelines involving sequential inference, hardware limitations are minimal. By estimating the expected virtual random access memory (VRAM) usage detailed in Note S2 with the unveiled number of parameters in Figure 1, even the largest evaluated model, EquiformerV2–153M, requires approximately 3.4 GB of VRAM. This places state-of-the-art MLIPs well within the capabilities of consumer-grade GPUs of around 4 GB of VRAM. For single-structure evaluations, the memory footprint is dominated by the fixed model framework overhead rather than the model architecture itself. Consequently, as accessibility is high, users generally do not require high-performance computing to sequentially run MLIP predictions.

The landscape changes when parallelization tools are employed or during batched inference. This contrast becomes evident in scenarios such as high-throughput screening, prediction of transition state structures, or batched training, where energies, forces, and intermediate states must be stored for multiple structures simultaneously. While the model weights (*N*_param_) remain static, the memory usage scales linearly with the number of atoms and the batch size. Graph neural networks (GNNs) store dense edge features for each atom in the batch. As the batch size increases, even a 12 GB GPU can quickly encounter out-of-memory errors. For instance, while EquiformerV2-83M fits easily in memory for a single structure, increasing the batch size to process 100 structures simultaneously could demand upwards of 20–30 GB of VRAM, pushing the requirement into the territory of HPC-needed resources.

Thus, a trade-off between throughput and memory constraints becomes evident. Parallelization maximizes compute utilization, preventing core idling between small tasks, but it requires exponential increases in memory availability. For standard hardware, the optimal strategy often involves microbatching to limit parallel structures to modest sizes or utilizing data parallelism to distribute batches across multiple consumer GPUs rather than relying on a hardware unit.

In summary, while computational costs vary significantly across architectures, all evaluated models remain within a competitive time scale. The observed variance in execution time suggests that the models are sensitive to the differing physicochemical complexities of the metal surfaces. Nevertheless, a clear distinction in hardware prerequisites emerges based on the deployment scale. While MLIPs have been democratized for single-trajectory simulations, large-scale parallel screening remains a memory-bound problem that benefits significantly from high-VRAM hardware.

Following the time benchmark, we simultaneously assessed structural fidelity and energy accuracy by comparing predicted adsorption heights and energies against reference DFT data.^80^ Adsorption heights have been defined as the minimum distance between the adsorbate and the surface plane on the *z*-axis. Adsorption energies are defined as the variation in energy when a chemical species, the adsorbate, binds to the surface. Details on the definition of these parameters are available in Note S3. Detailed results for MLIP vs DFT comparisons can be found in Figures S1–S6, Tables S6–S10.

The capability of “out-of-the-box” MLIPs to serve as DFT surrogates hinges on their accuracy in predicting fundamental adsorption properties. The parity plots in Figure 2 reveal that higher-parameter models, specifically OCP, achieve qualitative agreement when predicting adsorption energies (*E*_ads_) and geometric distances (*d*_ads_). However, while large-scale models capture adsorbate–surface interactions better than lighter architectures, deviations in prediction tend to exceed the standard chemical accuracy threshold of 0.2 eV. This indicates that, while these models are effective for identifying trends, quantitative energy prediction is not guaranteed for thermodynamic analysis without DFT verification. MACE models also demonstrate good balance, closely mirroring the DFT ground truth with minimal computational expense. While lighter models, such as Orb and SevenNet, exhibit a wider scatter in their predictions, they are able to capture the broader trends. This distinction suggests a tiered application strategy, where lighter models serve as rapid prescreening tools to filter candidate catalysts, while heavier, more accurate models are reserved for final filtering prior to final DFT validation.

**Figure 2:**
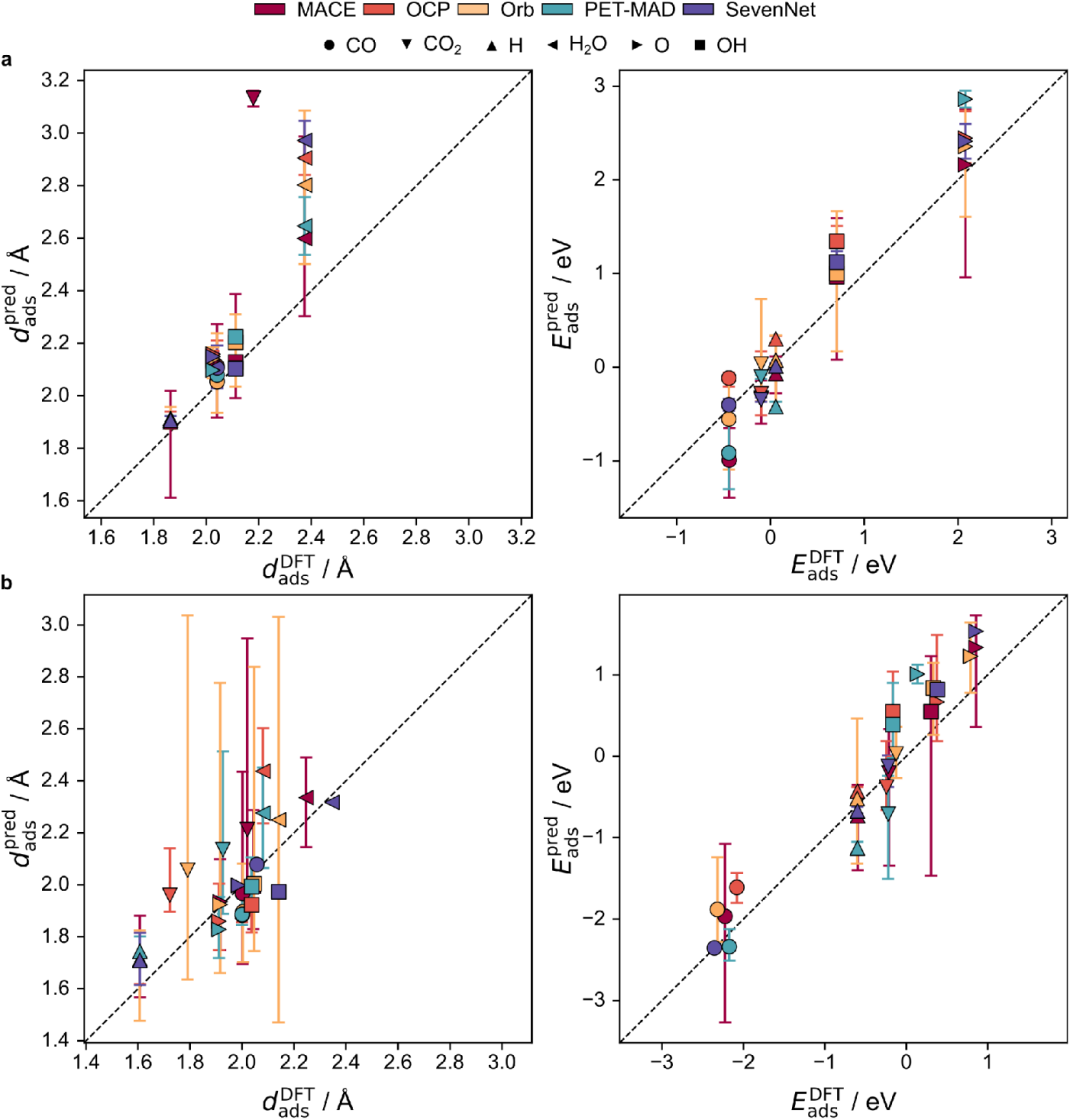
Parity plots of MLIP-predicted vs DFT-calculated (PBE-D3) adsorption properties (left: mean adsorption lengths, *d*_ads_, right: mean adsorption energies, *E*_ads_) for selected adsorbates (CO, CO_2_, H, OH, O, H_2_O) for (a) Ag, Au and (b) Ni, Pd. Predictions with |*E*_pred_ – *E*_DFT|_> 1.0 eV or |*d*_pred_ – *d*_DFT_| > 1.0 Å have been excluded for clarity. Vertical bars indicate the min and max range of predictions within each model family. Dashed lines represent ideal parity. Detailed data are available in Tables S6–S10.

Translating adsorption energies to reaction paths provide the ultimate test for the MLIP transferability. Although the propagation of errors may affect the predicted energies of intermediate species, systematic error cancellation can preserve the expected reaction trends. Figure 3 delineates the thermodynamics for the reaction profiles of the RWGS reaction on noble (Ag and Au) and reactive (Ni and Pd) metals. Detailed thermodynamic results for each step can be found in Table S11. On inert surfaces, most models qualitatively reproduce the trends of the energy profile, though lightweight models can overestimate some reaction steps. The distinction in model capability becomes pronounced on reactive surfaces such as Ni(111) and Pd(111), where strong adsorbate–surface hybridization occurs. Here, the OCP models demonstrate remarkable robustness, reproducing the steps predicted by DFT with high fidelity. MACE similarly captures these complex features, proving that efficient equivariant architectures can compete with larger transformer models. In contrast, other models struggle to resolve the stability of specific intermediates on magnetic or highly reactive surfaces.

**Figure 3:**
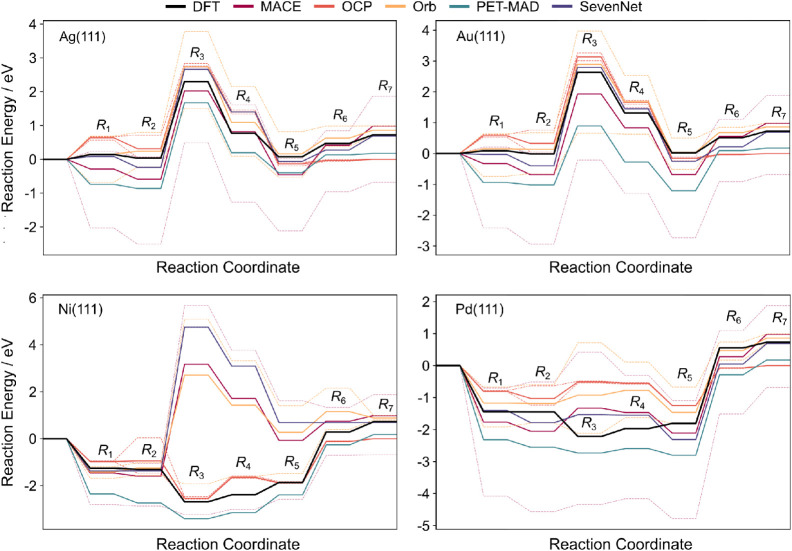
Reaction energy profiles for the reverse water–gas shift (RWGS) reaction on Ag(111), Au(111), Ni(111), and Pd(111) surfaces from varying MLIP model families vs DFT (PBE-D3) calculations (black line). Thick lines represent the mean predicted values for each model family. Dashed lines represent the min and max regions of prediction for each model family. Punctual Orb models have been removed from the Ni(111) plot for visual clarity. Reaction labels: R_1_: H_2_(g) → 2H*, R_2_: ${\mathrm{CO}}_{2}\left(g\right)\to {\mathrm{CO}}_{2}^{*}$ , R_3_: ${\mathrm{CO}}_{2}^{*}\to {\mathrm{CO}}^{*}+O^{*}$ , R_4_: O* + H* → OH*, R_5_: OH*+ H* → H_2_O*, R_6_: CO* → CO­(g), R_7_: H_2_O* → H_2_O­(g).

However, chemical limitations can be observed across the MLIP landscape regarding the treatment of adsorbed oxygen species (O*, OH*) present in *R*_3–5_ on Group 10 metals like Ni and Pd. The distorted energy steps involving these oxygenated species suggest that many current models struggle to fully resolve the complex chemistry driving O* and OH* adsorptions on these surfaces, likely due to difficulties in capturing the strong interactions between *p*-orbitals of oxygen with the d-bands of the surface and charge transfer effects inherent to these systems. Consequently, while the trend (global profile shape) is preserved by most models, the quantitative description of oxygen–metal chemistry remains a challenging frontier where MLIPs occasionally diverge from the DFT ground truth. Benchmarking of the MLIPs against the additional PBE, RPBE, and BEEF-vdW functionals (Note S1 and Figures S7–S8 reveal similar trends.)

In summary, the current landscape of MLIPs offers a versatile toolkit for accelerating traditional DFT methodologies as fast screening tools rather than as a replacement. The OCP framework establishes a good baseline for accuracy, reproducing the reaction trends in complex environments. However, the persistence of high errors on strongly correlated steps for Ni and Pd surfaces leads to reaction shifts of up to 3 eV, confirming that current pretrained models are insufficient as DFT replacements. Thus, models such as OCP and MACE emerge as optimal prescreening tools, offering the necessary balance between speed and accuracy to screen and filter candidate configurations before a necessary final DFT refinement. Collectively, these results confirm that MLIPs have matured from bulk data generators to practical computational tools capable of accelerating the discovery of catalytic materials.

## Conclusion and Outlook

The emergence of machine learning interatomic potentials (MLIPs) marks a paradigm shift in the fields of computational materials science and catalysis. However, realizing their full potential requires navigating specific limitations while leveraging complementary strengths alongside traditional methods.

### Challenges: Hardware, Reliability, and Standardization

For widespread adoption in heterogeneous catalysis, MLIPs must first demonstrate excellent computational efficiency. A critical, often overlooked challenge is the practical implementation of these models on commodity workstations. While MLIPs accelerate individual calculations, the memory footprint and inference costs of state-of-the-art models often preclude their efficient implementation in parallelized workflows on standard hardware. Consequently, the reliance on high-performance computing remains a necessity, limiting their accessibility for rapid, decentralized application.

Furthermore, while this perspective focused primarily on predictive accuracy and speed, widespread adoption hinges on operational reliability. First, robust uncertainty quantification is a prerequisite for human-in-the-loop workflows. Without the ability to estimate confidence levels, MLIPs remain “black boxes”. Second, the field faces a critical bottleneck in data standardization. Despite the increasing availability of computational databases, the lack of unified formats impedes the seamless aggregation of data sets, hindering both the development and benchmarking of current and new MLIPs. This fragmentation extends to the models themselves, where a lack of consistency across institutional frameworks further hinders practical integration.

Finally, a significant barrier is that MLIPs are not a readily reliable universal, “out-of-the-box” replacement for density functional theory (DFT). The data reveal that even the most accurate models carry large inference errors of ∼0.5 eV. While remarkable, this performance does not yet consistently meet the accuracy threshold of DFT (typically defined as 0.2 eV) required for precise mechanistic studies.

### Opportunities: Synergistic Workflows and Active Learning

Despite these gaps, the true utility of MLIPs today lies in their synergistic integration into existing computational workflows. Their ability to reduce execution times by 4–6 orders of magnitude compared to classical DFT can be leveraged to augment and accelerate, rather than supplant, first-principles methods.

First, MLIPs are optimal accelerators for geometry optimization. A significant portion of the computational cost in any DFT run is spent iteratively relaxing the initial structure. Models like MACE, Orb, and PET-MAD can perform this relaxation ∼10^4^ times faster than DFT. The resulting preoptimized structures serve as an ideal starting point for a final, high-accuracy DFT refinement, drastically reducing the required optimization steps.

Second, these models are transformative for high-throughput screening. When discovering new catalysts, researchers can use MLIPs as surrogate models to rapidly predict adsorption energies across vast material landscapes. While absolute values carry inherent errors, these models are exceptionally adept at capturing underlying qualitative chemical trends, efficiently guiding the usage of extensive computational resources toward the most promising candidates.

Finally, although it is out of the scope of this perspective, the integration of active learning workflows represents a promising frontier. By leveraging the aforementioned uncertainty quantification, models can move beyond static screening to dynamic iterative discovery. In this loop, an MLIP can identify regions of the chemical space where its predictions are uncertain and autonomously request ground-truth DFT calculations for specific configurations. This data is then fed back to retrain the model, progressively reducing the error gap. Such workflows represent the ideal synergy between machine learning and *ab initio* methodologies, minimizing the expensive DFT calls while maximizing the exploration of complex landscapes.

## Supplementary Material



## Notes

Detailed definitions of the adsorption parameters, estimations of VRAM memory usage, and the complete results of the comparative MLIP benchmark (PDF)
