# Supplementary material for: Challenges and Opportunities of Pretrained Machine Learning Interatomic Potentials in Heterogeneous Catalysis
Source: ACS Catal. 2026 Feb 18;16(5):4113–24. doi: 10.1021/acscatal.5c08945 (PMC12976938; doi:10.1021/acscatal.5c08945)
Supplement: Supplementary file 1 [file cs5c08945_si_001.pdf]

# Supporting Information: Challenges and Opportunities of Pre-Trained Machine Learning Interatomic Potentials in Heterogeneous Catalysis

Oliver Loveday,[*,†,‡] Kamila Kazmierczak,[¶] and Núria López[*,†]

†*Institute of Chemical Research of Catalonia (ICIQ-CERCA), The Barcelona Institute of Science and Technology, Av. Països Catalans 16, 43007, Tarragona, Spain*

‡*Department of Physical and Inorganic Chemistry, Universitat Rovira i Virgili, Campus Sescelades, N4 Block, C. Marcellí Domingo 1, 43007, Tarragona, Spain*

¶*TotalEnergies, TotalEnergies One Tech Belgium, Zone industrielle C, 7181 Feluy, Belgium*

E-mail: oloveday@iciq.es; nlopez@iciq.es

## Keywords

Machine Learning, MLIPs, Foundational Models, Heterogeneous Catalysis, RWGS

# Supplementary Notes

## Note S1. Benchmark of MLIPs against different DFT functionals

To provide a broader landscape on the accuracy of the predictive capabilities of the MLIPs, an energy benchmark against DFT singlepoint calculations at different leves of theory has been performed. This benchmark stems from the known caveats of PBE, summed with the inclusion of D3 dispersion, to provide overbinding energy values for chemisorbed systems [1, 2]. Thus, the PBE[3], RPBE[4] and BEEF-vdW[5] functionals have been selected as benchmarks for a better understanding of the limitations of the MLIPs presented in this work.

The singlepoint calculations have been carried out with similar computational details as in Ref.[6]: The Vienna Ab Initio Simulation Package[7] (VASP 5.4.4) was used as the computational calculation software. Core electrons treated with the Projector Augmented Wave (PAW) method[8, 9] and valence electrons expanded with plane waves with a basis set cutoff of 450 eV. Electronic convergence was set to $1{\cdot}10^5$ eV. Slabs were represented with a 4-layered $p(2\sqrt{3}{\times}2\sqrt{3})$-R30° supercell, with a vacuum of 12 Å. The Brillouin zone was sampled by a Γ-centered 3×3×1 $k$-point mesh. Dipole in the $z$ direction and spin polarization have been considered. For BEEF-vdW calculations, non-spherical contributions from the gradient of density inside the PAW spheres were included, and the $Z_{ab}$ parameter was set to -1.8867 [-][10].

Reaction profiles for the different functionals (**Figure SS7**) and benchmarked against the different MLIPs (**Figure SS8**) on the different metals highlight the overbinding trend of PBE-D3 compared to standard PBE. This is well known since the inclusion of D3 dispersion, which accounts for van der Waals interactions, stabilizes chemisorbed species. RPBE reduces the adsorption of each species by ≈ 0.4 eV when oxygen-like adsorbates are present, and BEEF-vdW is a well-balanced functional across the different metal surfaces. As for the accuracy performance of the MLIPs, while an adjustment in their energy predictions can be

observed, where values fall in-between these DFT regions, the struggle when predicting Ni and Pd surfaces can still be observed.

## Note S2. Estimation of computational memory requirements

To provide a upper bound for hardware requirements, we employ a conservative estimation model that accounts for peak dynamic activation expansion. Unlike standard neural networks, equivariant graph neural networks perform high-order tensor products that generate large temporary intermediate states, even for small atomic systems.

The estimated safe VRAM allocation, $M_{\text{safe}}$, is defined as:

$$M_{\text{safe}} \approx \mathcal{C}_{\text{ctx}} + (N_{\text{param}} \cdot \rho_{\text{prec}}) \cdot K_{\text{arch}} \tag{1}$$

where $\mathcal{C}_{\text{ctx}}$ stands for the framework context of 1024 MB. We adopt a baseline for modern PyTorch environments which often pre-allocate large caching blocks. $N_{\text{param}} \cdot \rho_{\text{prec}}$ is the raw size of the model weights (4 Bytes per parameter for a float32 precision).$K_{\text{arch}}$ is the expansion factor, set to a value of 4.0 [11]. This multiplier accounts for the standard memory footprint of the Adam optimizer states and gradient buffers. Furthermore, it serves as a conservative buffer for the significant activation overhead observed in equivariant networks, where high-order tensor products generate large intermediate representations [12].

## Note S3. Adsorption height and energy definition

To assess the structural configurations, the adsorption height ($d_{\text{ads}}$) was computed as the absolute vertical distance along the surface normal ($z$-axis) between the lowest atom of the adsorbate and the highest atom of the surface slab:

$$d_{\text{ads}} = |z_{\text{min}}^{\text{adsorbate}} - z_{\text{max}}^{\text{surface}}| \tag{2}$$

The adsorption energy ($E_{\text{ads}}$) has been defined as:

$$E_{\text{ads}} = E_{\text{tot}} - E_{\text{surf}} - E_{\text{adsorbate(gas)}} \tag{3}$$

where $E_{\text{tot}}$ represents the total energy of the relaxed adsorbate-surface system, $E_{\text{surf}}$ is the energy of the clean metal slab, and $E_{\text{adsorbate(gas)}}$ corresponds to the energy of the isolated adsorbate molecule in the gas phase. For the OCP models, the gas-phase energy was approximated according to their set of atomic reference energies (in eV, C: -7.282, H: -3.477, O: -7.204) [13].

# Supplementary Figures

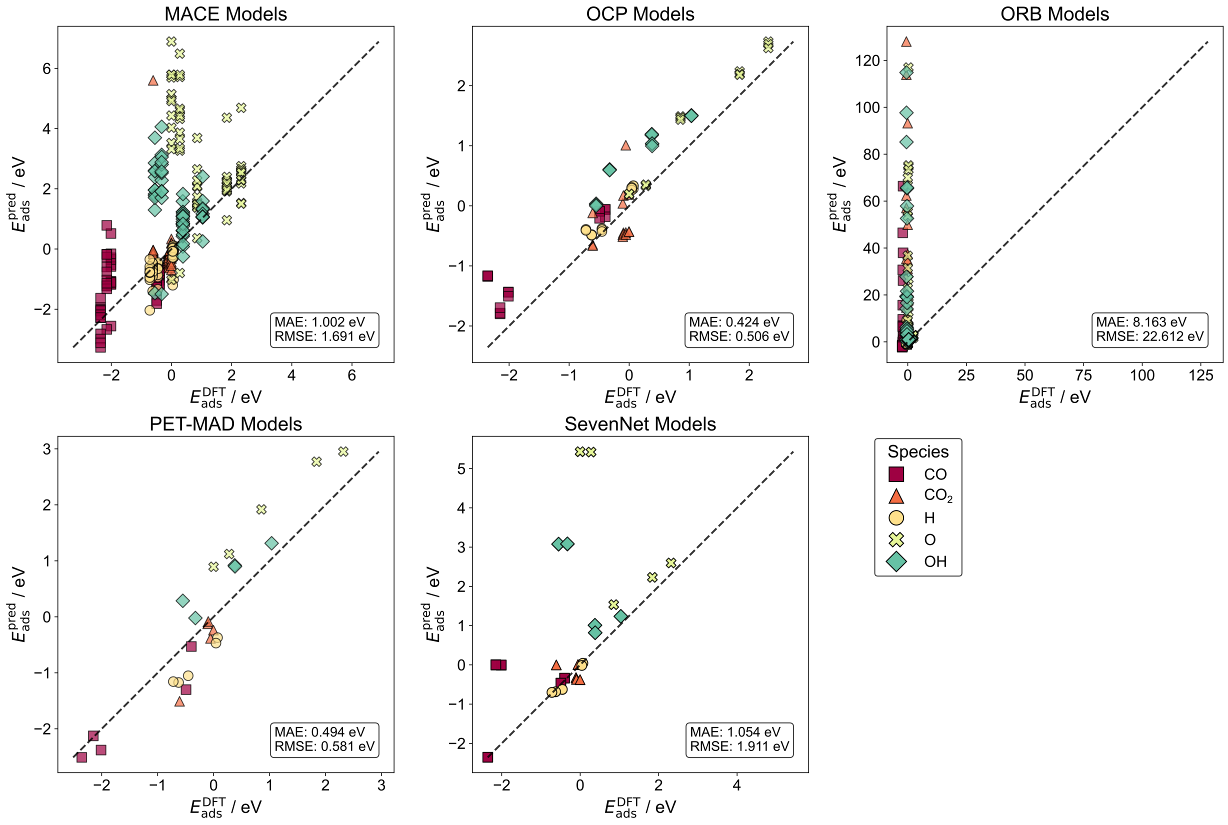


**Figure S1:** Overall parity plots comparing predicted vs. DFT energies for each model family. OCP shows the best correlation, while MACE and ORB suffer from significant outliers.

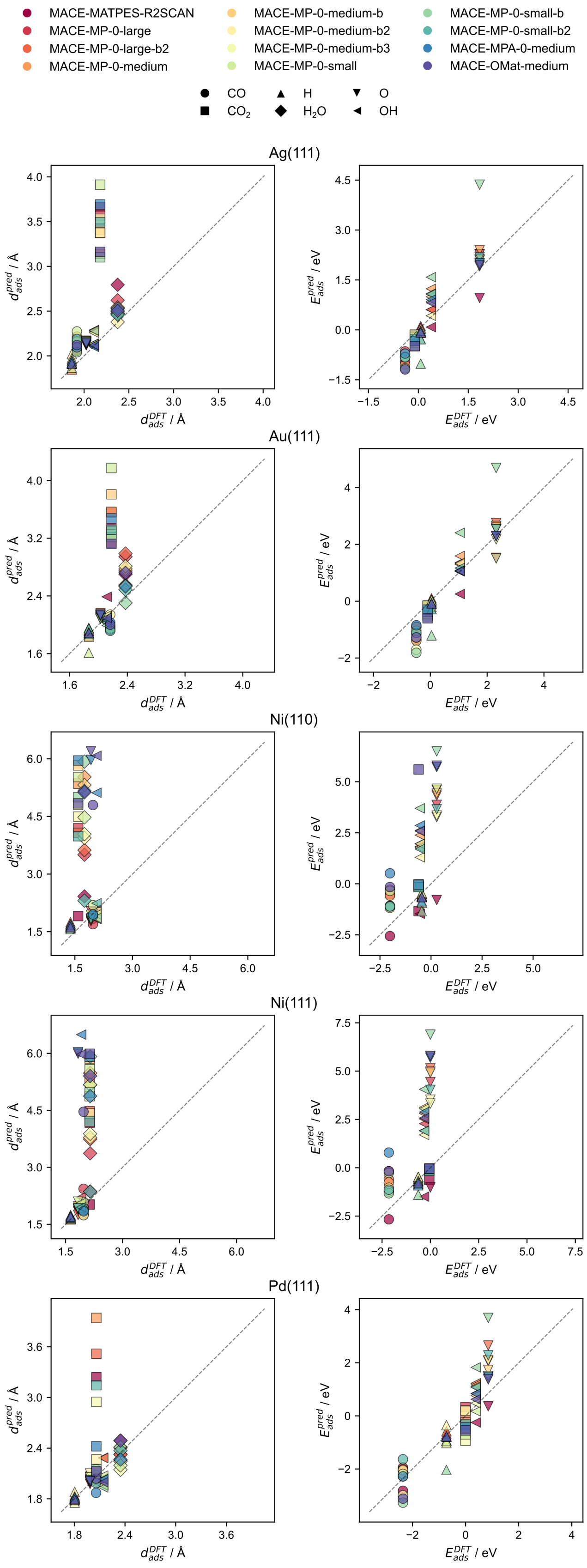


**Figure S2:** Surface-specific parity plots for the MACE model family with respect to DFT (PBE-D3). Left: Adsorption heights. Right: Adsorption energies.

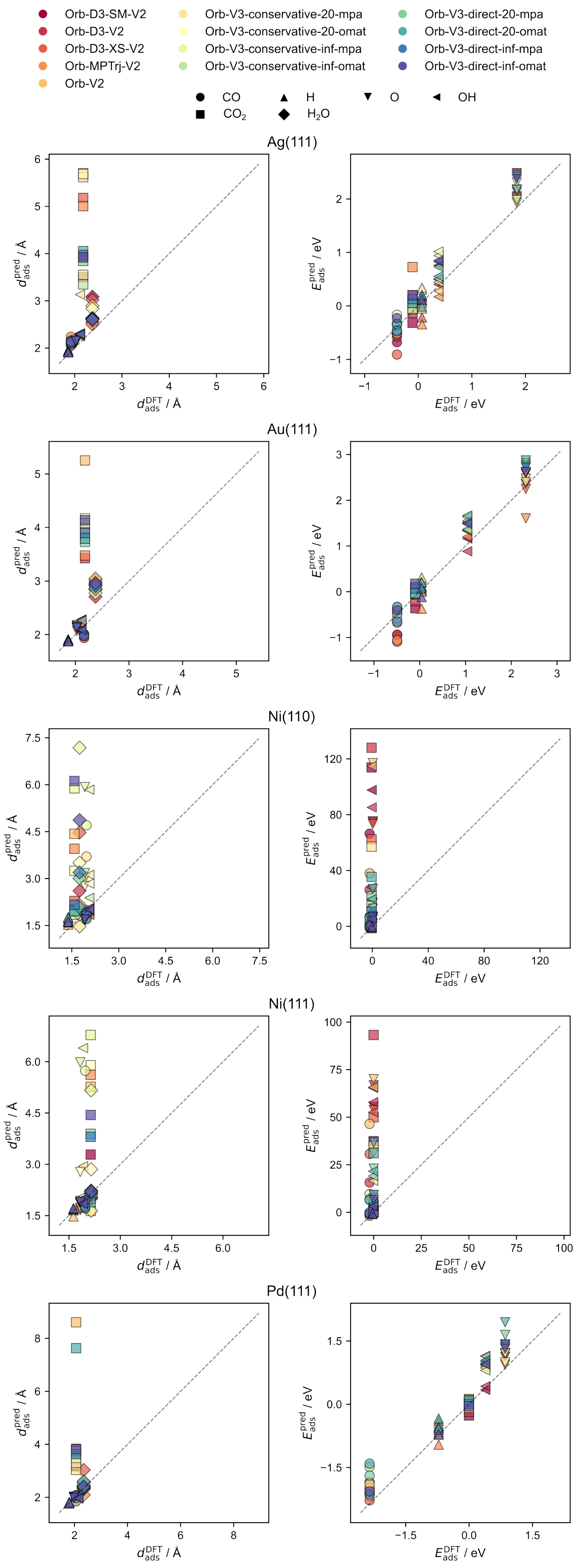


**Figure S3:** Surface-specific parity plots for the Orb model family with respect to DFT (PBE-D3). Left: Adsorption heights. Right: Adsorption energies.

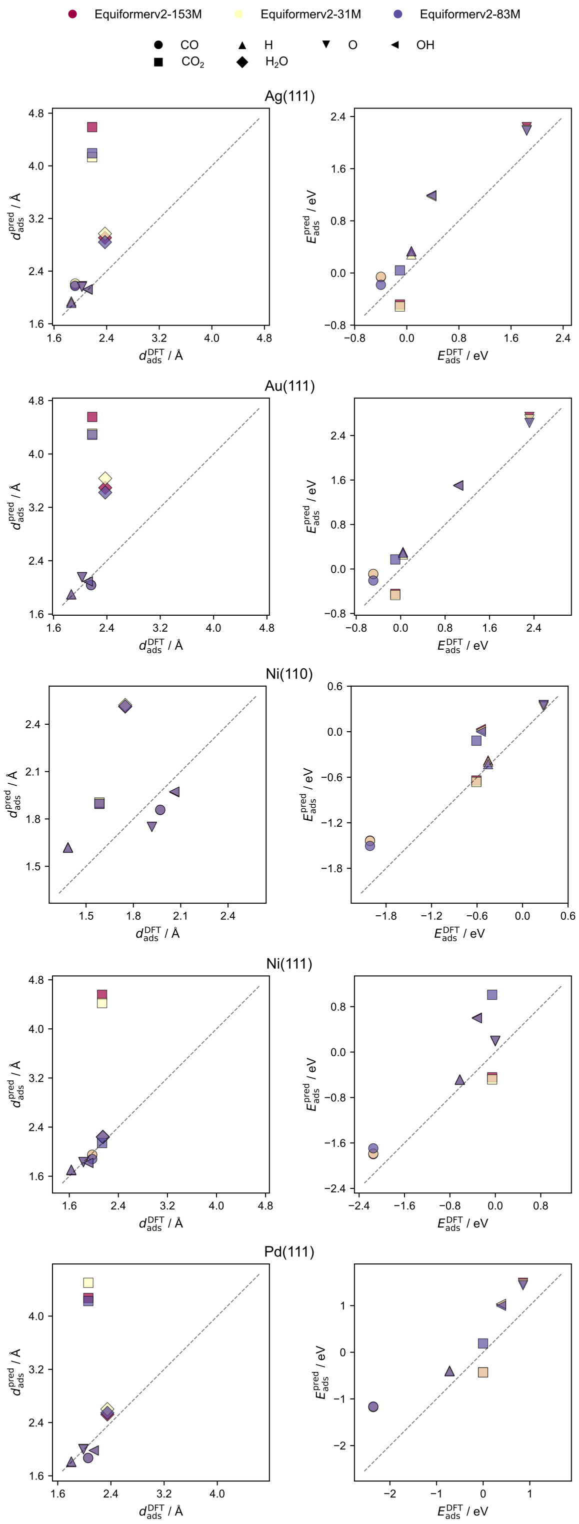


**Figure S4:** Surface-specific parity plots for the OCP model family with respect to DFT (PBE-D3). Left: Adsorption heights. Right: Adsorption energies.

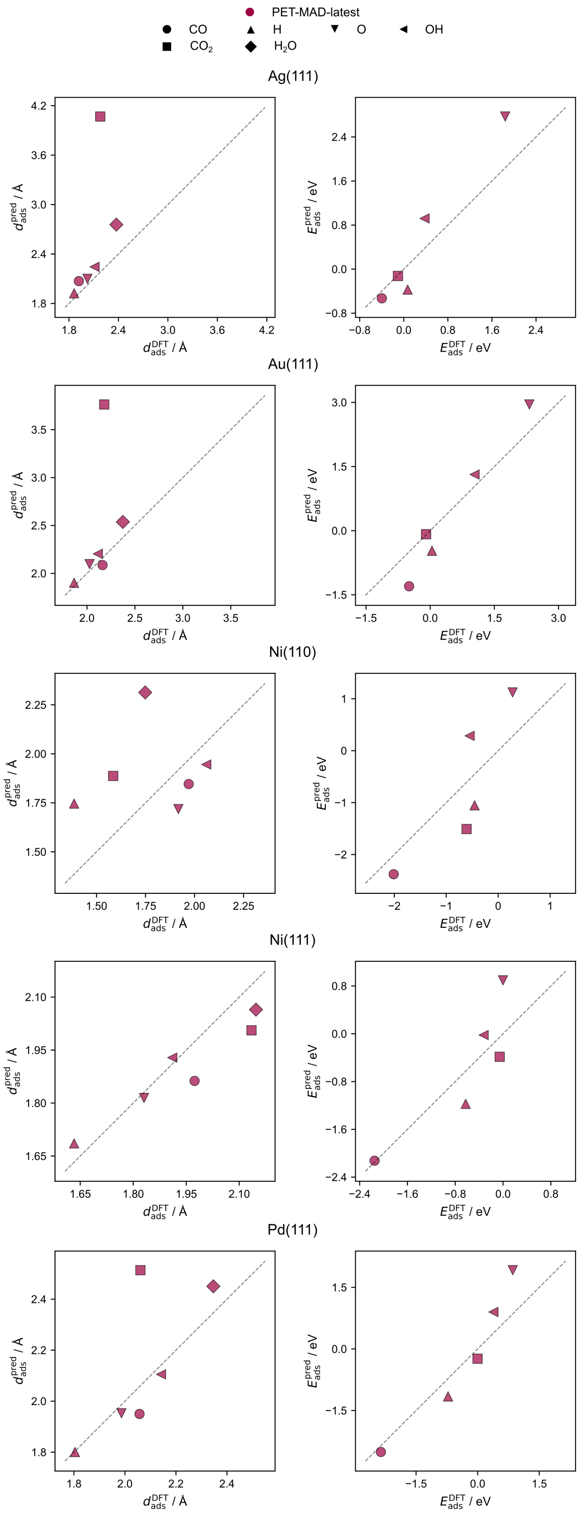


**Figure S5:** Surface-specific parity plots for the PET-MAD model family with respect to DFT (PBE-D3). Left: Adsorption heights. Right: Adsorption energies.

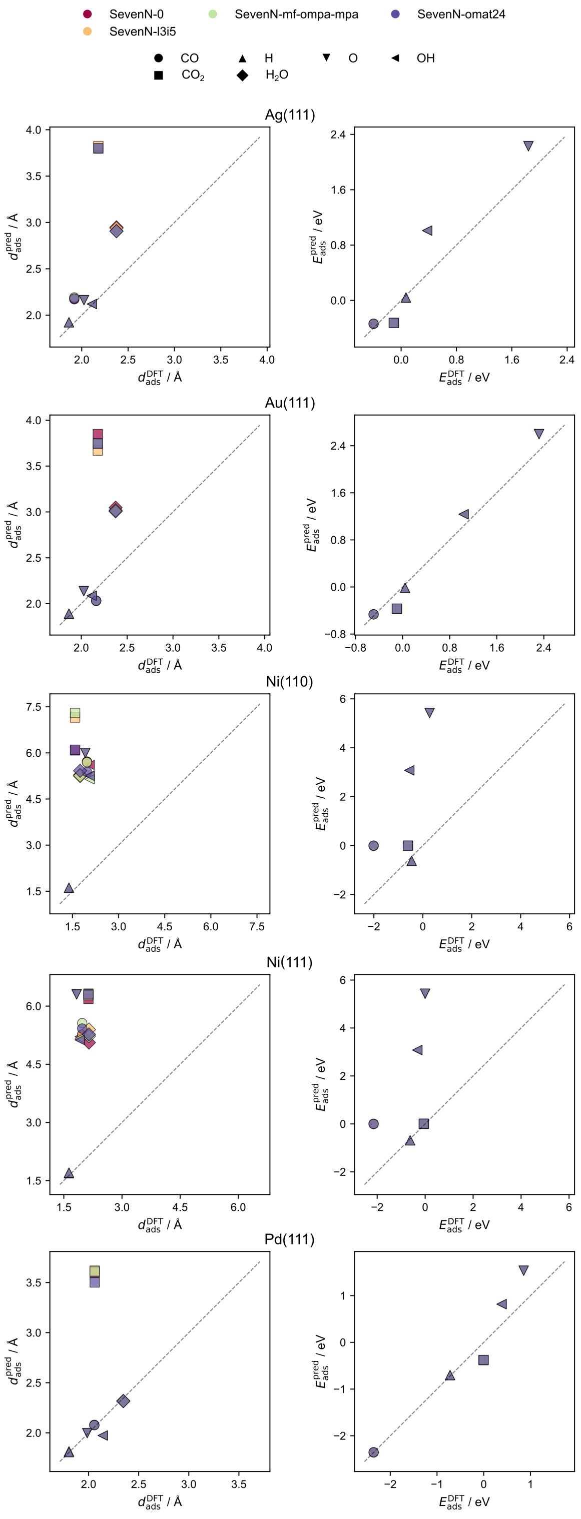


**Figure S6:** Surface-specific parity plots for the SevenNet model family with respect to DFT (PBE-D3). Left: Adsorption heights. Right: Adsorption energies.

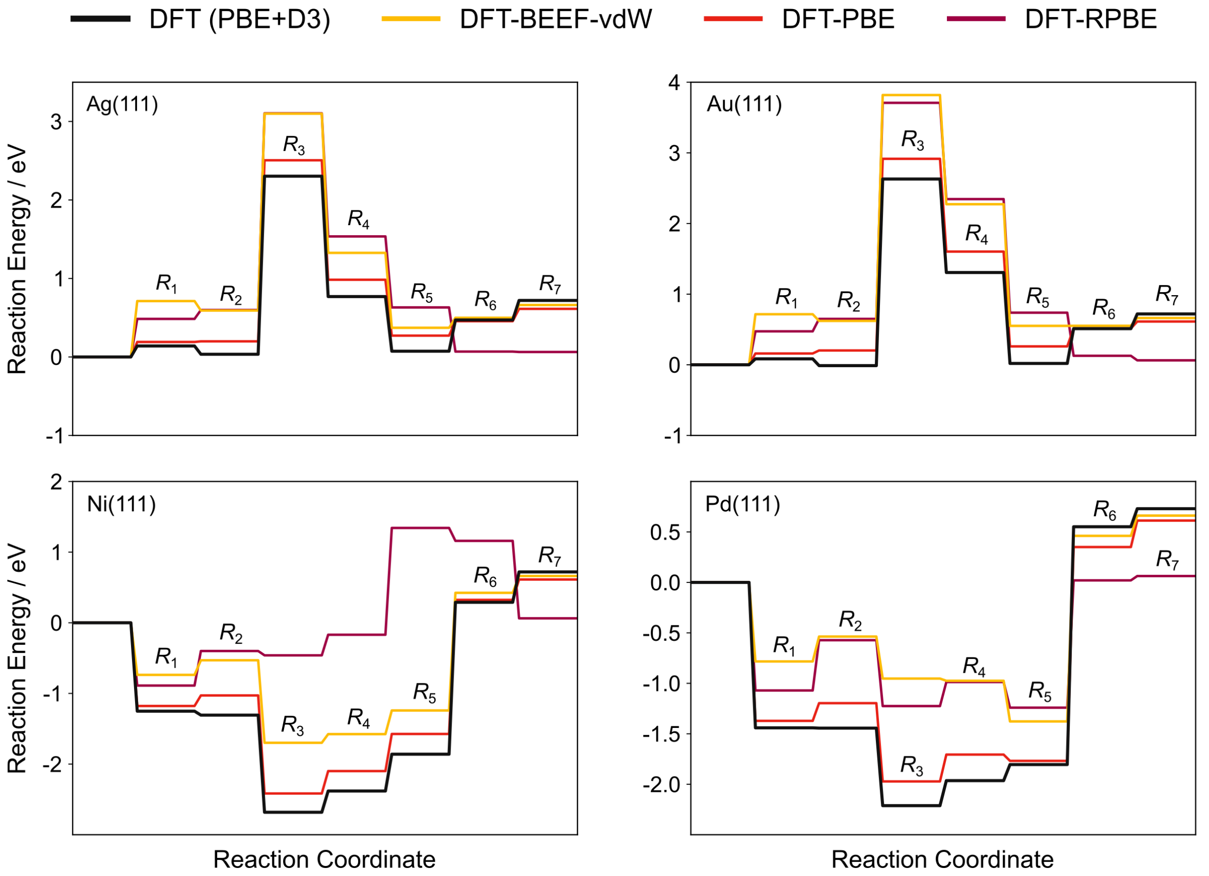


**Figure S7:** Reaction energy profiles for the reverse water gas shift (RWGS) reaction on Ag(111), Au(111), Ni(111), and Pd(111) surfaces for different DFT functionals (PBE, PBE-D3, RPBE, and BEEF-vdW). Reaction labels: $R_1$: $H_2(g) \longrightarrow 2\,H^*$, $R_2$: $CO_2(g) \longrightarrow CO_2{}^*$, $R_3$: $CO_2{}^* \longrightarrow CO^* + O^*$, $R_4$: $O^* + H^* \longrightarrow OH^*$, $R_5$: $OH^* + H^* \longrightarrow H_2O^*$, $R_6$: $CO^* \longrightarrow CO\,(g)$, $R_7$: $H_2O^* \longrightarrow H_2O\,(g)$.

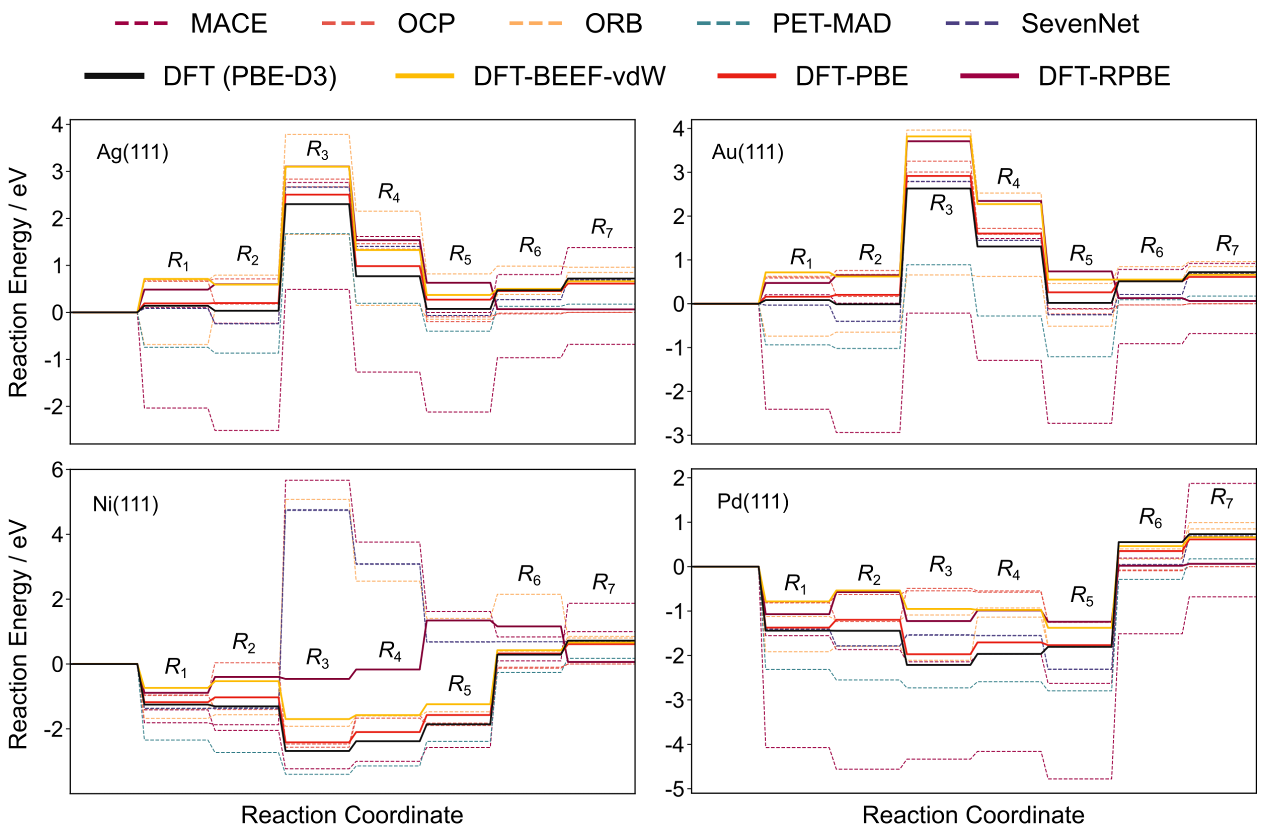


**Figure S8:** Reaction energy profiles for the reverse water gas shift (RWGS) reaction on Ag(111), Au(111), Ni(111), and Pd(111) surfaces of the different MLIPs (dashed lines) for different DFT functionals (thick lines, PBE, PBE-D3, RPBE, and BEEF-vdW). Dashed lines represent the min,max regions of prediction for each model family. Punctual Orb models have been removed from the Ni(111) plot for visual clarity. Reaction labels: $R_1$: $H_2(g) \longrightarrow 2\,H^*$, $R_2$: $CO_2(g) \longrightarrow CO_2{}^*$, $R_3$: $CO_2{}^* \longrightarrow CO^* + O^*$, $R_4$: $O^* + H^* \longrightarrow OH^*$, $R_5$: $OH^* + H^* \longrightarrow H_2O^*$, $R_6$: $CO^* \longrightarrow CO\,(g)$, $R_7$: $H_2O^* \longrightarrow H_2O\,(g)$.

# Supplementary Tables

**Table S1:** Model architecture and specifications for state-of-the-art MLIPs, classified by developing families.

| Family | Model Name | Architectural Class | Model Size (Parameters) | Elements Covered |
|---|---|---|---|---|
| MACE | MACE-MP-0 | *E(3)*-equivariant MPNN with higher-order messages (Multi-ACE) | small, medium, large | 89 |
| | MACE-MPA-0 | | medium | 89 |
| | MACE-OMAT | | small, medium | 89 |
| | MACE-MATPES-PBE-0 | | medium | 89 |
| | MACE-MATPES-r2SCAN-0 | | medium | 89 |
| OCP | DimeNet++ | Directional MPNN | 1.8M / 18.8M / 240.8M | 89 |
| | GemNet | Geometric MPNN | 31M / 300M | 89 |
| | eSCN(OC20) | Equivariant Steerable Convolutional Network | 200M | 89 |
| | EquiformerV2(OC20) | Equivariant transformer with eSCN convolutions | 31.2M / 86.6M / 153.8M | 89 |
| | EquiformerV2(OC22) | Equivariant transformer with eSCN convolutions | 121.8M | 95 |
| CHGNet | CHGNet | Charge-Informed MPNN (Magnon Regularization) | 400,438 | 94 |
| M3GNet | M3GNet | MPNN | ∼228k learnable weights | 94 |
| SevenNet | SevenNet-0 | Optimized NequIP for Parallel MD | 840k | 89 |
| | SevenNet-IS | Optimized NequIP (Higher Accuracy, lmax=3) | 1.17M | 89 |
| | SevenNet-MF-0 | Multi-Fidelity Trained NequIP | 3.27M | 89+ |
| PET-MAD | PET-MAD v1.0.2 | Rotationally unconstrained and transformer-based GNN | 2.8M | 94 |
| ORB | ORB v1 | Attention-Augmented GNS (MPNN) | Not specified | 117 |
| | ORB v2 | Attention-Augmented GNS (Smoothed cutoff) | 25.2M | |
| | ORB v3 | Attention-Augmented GNS (Wider, fewer layers) | 25.5M | |
| Matlantis | PFP v1 | Equivariant GNN (Proprietary) | Not specified | 55 |
| | PFP v3 | | | 72 |
| | PFP v5 | | | |
| ALIGNN | ALIGNN-JARVIS-DFT | Atomistic Line Graph Neural Network (Force Field) | ∼ 4M | 89 |
| | ALIGNN-MP | | - | 118 |
| | ALIGNN-QM9 | | - | 5 (C,H,O,N,F) |
| | ALIGNN-hMOF | | Not specified | - |
| | ALIGNN-FF | | Not specified | 89 |
| | ALIGNN-OCP | | - | 89 |
| | ALIGNN-InterMat | | Not specified | 89 |

**Table S2:** Domain application and training details of state-of-the-art MLIPs (Part 1).

| Family | Model Name | Domain | Level of Theory (Training) | Core Training Set(s) |
|---|---|---|---|---|
| MACE | MACE-MP-0 | Universal Materials | DFT (PBE+U) with VASP | MPTraj |
| | MACE-MPA-0 | Universal Materials | DFT | MPTraj, sAlex |
| | MACE-OMAT-0 | Universal materials | DFT | OMat |
| | MACE-OFF | Organic & Biomolecules | DFT (wB97M-V/def2) | SPICE |
| | MACE-MATPES-PBE-0 | Universal Materials (No +U) | DFT (PBE) | MATPES-PBE |
| | MACE-MATPES-r2SCAN-0 | Universal Materials (r2SCAN) | DFT (r2SCAN) | MATPES-r2SCAN |
| OCP | DimeNet++ | Heterogeneous Catalysis | DFT (PBE) | OC20 |
| | GemNet | | | |
| | EquiformerV2(OC20) | | | |
| | eSCN(OC20) | | | |
| | EquiformerV2(OC22) | Universal Materials | DFT (PBE+U) | OC22 |
| CHGNet | CHGNet | Universal Materials (esp. batteries, magnetic) | DFT (PBE+U) with VASP | MPTraj |
| M3GNet | M3GNet | Molecular & Crystal Properties | DFT (B3LYP / PBE) | QMx, MP-crystals-2018 |
| SevenNet | SevenNet-0 | Universal Materials (scalable MD) | DFT (PBE/PAW) with VASP | MPTraj |
| | SevenNet-IS | | DFT (various) | OMat24, MPTraj, sAlex, MD17, etc. |
| | SevenNet-MF-0 | | | |

**Table S3:** Domain application and training details of state-of-the-art MLIPs (Part 2).

| Family | Model Name | Domain | Level of Theory (Training) | Core Training Set(s) |
|---|---|---|---|---|
| ORB | ORB v1 | Universal Materials (Molecules, MOFs, etc.) | DFT (PBE) with VASP | MPTraj, Alexandria |
| | ORB v2 | Universal Materials (esp. dispersion) | | |
| | ORB v3 | Universal Materials (Molecules, MOFs, etc.) | | OMat24, MPTraj, Alexandria |
| Matlantis | PFP v1 | Universal Materials (Commercial Platform) | DFT (PBE, uB97X-D) | PFP dataset (10M+ structures) |
| | PFP v3 | | | PFP dataset (22M+ structures) |
| | PFP v5 | | | PFP dataset (42M+ structures) |
| ALIGNN | ALIGNN-JARVIS-DFT | Solid-State Properties | DFT (OptB88vdW) | JARVIS-DFT |
| | ALIGNN-MP | Bulk materials | DFT (PBE) | Materials Project |
| | ALIGNN-QM9 | Organic Molecules | DFT (B3LYP) | QM9 |
| | ALIGNN-hMOF | MOF Properties | DFT / GCMC | MOF-FF (JARVIS) |
| | ALIGNN-FF | Structure Optimization & MD | DFT (OptB88vdW) | JARVIS-FF (force data) |
| | ALIGNN-OCP | Catalysis | DFT (PBE) | OC20 |
| | ALIGNN-InterMat | Semiconductor Interfaces | DFT (PBE, OptB88vdW) | JARVIS-DFT, MLEARN |

**Table S4:** Overview of package counts in each CARE environment.

| Environment | Total Packages | Unique Packages |
|---|---|---|
| `care-mace` | 182 | 7 |
| `care-ocp` | 190 | 12 |
| `care-orb` | 215 | 45 |
| `care-petmad` | 180 | 8 |
| `care-sevennet` | 172 | 2 |

**Table S5:** Estimated VRAM consumption for the evaluated MLIP models. The "Safe Allocation" accounts for a fixed framework overhead ($\mathcal{C}_{ctx} = 1024$ MB) and dynamic expansion ($K_{arch} = 4.0$). The estimations have been performed according to **Note S1**

| Model Name | Parameters ($N_{\text{param}}$, Millions) | Raw Weights (float32, MB) | Est. Peak VRAM ($M_{\text{safe}}$, GB) |
|---|---|---|---|
| SevenN-0 | 0.8 | 3.2 | 1.01 |
| SevenN-l3i5 | 1.2 | 4.8 | 1.02 |
| PET-MAD-latest | 2.9 | 11.6 | 1.05 |
| MACE-MP-0-small | 6.8 | 27.2 | 1.11 |
| MACE-MP-0-medium | 8.3 | 33.2 | 1.13 |
| MACE-MATPES-R2SCAN | 9.1 | 36.4 | 1.14 |
| Orb-V2-D3-XS | 9.4 | 37.6 | 1.15 |
| MACE-MP-0-large-b2 | 10.1 | 40.4 | 1.16 |
| SevenN-omat24 | 15.7 | 62.8 | 1.25 |
| MACE-MP-0-large | 15.8 | 63.2 | 1.25 |
| Orb-V2-D3-SM | 17.3 | 69.2 | 1.27 |
| Orb-V2-Default | 25.2 | 100.8 | 1.39 |
| Orb-V3-conservative | 25.5 | 102.0 | 1.40 |
| Orb-V3-direct | 25.6 | 102.4 | 1.40 |
| SevenN-mf-ompa-mpa | 25.7 | 102.8 | 1.40 |
| EquiformerV2-31M | 31.1 | 124.4 | 1.49 |
| EquiformerV2-83M | 83.2 | 332.8 | 2.30 |
| EquiformerV2-121M | 121.5 | 486.0 | 2.90 |
| EquiformerV2-153M | 153.6 | 614.4 | 3.40 |

**Table S6:** Performance metrics for MACE models

| Model | Surface | Species | $d_{DFT}$ / Å | $d_{Pred}$ / Å | $\|\Delta d\|$ | $E_{DFT}$ / eV | $E_{Pred}$ / eV | $\|\Delta E\|$ |
|---|---|---|---|---|---|---|---|---|
| MACE-MATPES-R2SCAN | Ag(111) | CO | 1.92 | 2.13 | 0.21 | -0.40 | -0.95 | 0.56 |
| | | $CO_2$ | 2.18 | 3.66 | 1.48 | -0.11 | -0.16 | 0.05 |
| | | H | 1.86 | 1.92 | 0.06 | 0.07 | 0.11 | 0.04 |
| | | $H_2O$ | 2.37 | 2.79 | 0.42 | – | – | – |
| | | O | 2.02 | 2.12 | 0.10 | 1.84 | 0.96 | 0.88 |
| | | OH | 2.11 | 2.11 | 0.00 | 0.38 | 0.08 | 0.30 |
| MACE-MATPES-R2SCAN | Au(111) | CO | 2.16 | 1.98 | 0.18 | -0.49 | -1.17 | 0.68 |
| | | $CO_2$ | 2.18 | 3.34 | 1.16 | -0.10 | -0.27 | 0.17 |
| | | H | 1.87 | 1.89 | 0.02 | 0.04 | -0.11 | 0.15 |
| | | $H_2O$ | 2.37 | 2.70 | 0.33 | – | – | – |
| | | O | 2.03 | 2.11 | 0.08 | 2.32 | 1.53 | 0.79 |
| | | OH | 2.11 | 2.39 | 0.27 | 1.04 | 0.25 | 0.78 |
| MACE-MATPES-R2SCAN | Ni(110) | CO | 1.97 | 1.84 | 0.13 | -2.01 | -2.56 | 0.55 |
| | | $CO_2$ | 1.59 | 1.91 | 0.32 | -0.61 | -1.34 | 0.73 |
| | | H | 1.39 | 1.64 | 0.25 | -0.45 | -0.62 | 0.16 |
| | | $H_2O$ | 1.75 | 2.42 | 0.67 | – | – | – |
| | | O | 1.92 | 1.76 | 0.16 | 0.28 | -0.80 | 1.08 |
| | | OH | 2.06 | 1.96 | 0.10 | -0.55 | -1.47 | 0.92 |
| MACE-MATPES-R2SCAN | Ni(111) | CO | 1.97 | 1.87 | 0.10 | -2.15 | -2.67 | 0.52 |
| | | $CO_2$ | 2.13 | 2.02 | 0.11 | -0.06 | -0.63 | 0.58 |
| | | H | 1.63 | 1.73 | 0.10 | -0.63 | -0.71 | 0.08 |
| | | $H_2O$ | 2.15 | 2.36 | 0.22 | – | – | – |
| | | O | 1.83 | 1.84 | 0.01 | -0.00 | -1.02 | 1.02 |
| | | OH | 1.91 | 1.94 | 0.03 | -0.33 | -1.50 | 1.17 |
| MACE-MATPES-R2SCAN | Pd(111) | CO | 2.06 | 2.06 | 0.00 | -2.36 | -2.82 | 0.46 |
| | | $CO_2$ | 2.06 | 3.24 | 1.18 | -0.00 | -0.31 | 0.31 |
| | | H | 1.80 | 1.81 | 0.01 | -0.72 | -0.78 | 0.06 |
| | | $H_2O$ | 2.35 | 2.49 | 0.14 | – | – | – |
| | | O | 1.99 | 1.98 | 0.01 | 0.85 | 0.36 | 0.50 |
| | | OH | 2.14 | 2.29 | 0.15 | 0.38 | -0.25 | 0.63 |
| MACE-MP-0-large | Ag(111) | CO | 1.92 | 2.07 | 0.15 | -0.40 | -0.65 | 0.25 |
| | | $CO_2$ | 2.18 | 3.57 | 1.39 | -0.11 | -0.24 | 0.13 |
| | | H | 1.86 | 1.92 | 0.05 | 0.07 | -0.05 | 0.12 |
| | | $H_2O$ | 2.37 | 2.62 | 0.25 | – | – | – |
| | | O | 2.02 | 2.12 | 0.10 | 1.84 | 2.28 | 0.44 |
| | | OH | 2.11 | 2.29 | 0.18 | 0.38 | 0.61 | 0.23 |
| MACE-MP-0-large | Au(111) | CO | 2.16 | 1.93 | 0.23 | -0.49 | -0.89 | 0.40 |
| | | $CO_2$ | 2.18 | 3.56 | 1.38 | -0.10 | -0.19 | 0.10 |
| | | H | 1.87 | 1.91 | 0.05 | 0.04 | 0.10 | 0.06 |
| | | $H_2O$ | 2.37 | 2.94 | 0.57 | – | – | – |
| | | O | 2.03 | 2.14 | 0.11 | 2.32 | 2.55 | 0.24 |
| | | OH | 2.11 | 2.06 | 0.06 | 1.04 | 1.36 | 0.32 |
| MACE-MP-0-large | Ni(110) | CO | 1.97 | 1.70 | 0.27 | -2.01 | -0.52 | 1.49 |
| | | $CO_2$ | 1.59 | 4.19 | 2.61 | -0.61 | -0.12 | 0.49 |
| | | H | 1.39 | 1.70 | 0.31 | -0.45 | -0.49 | 0.04 |
| | | $H_2O$ | 1.75 | 3.50 | 1.75 | – | – | – |
| | | O | 1.92 | 2.03 | 0.12 | 0.28 | 3.87 | 3.59 |

Continued on next page

**Table S6 – Continued from previous page**

| Model | Surface | Species | $d_{DFT}$ / Å | $d_{Pred}$ / Å | $\lvert\Delta d\rvert$ | $E_{DFT}$ / eV | $E_{Pred}$ / eV | $\lvert\Delta E\rvert$ |
|---|---|---|---|---|---|---|---|---|
| | | OH | 2.06 | 2.08 | 0.02 | -0.55 | 1.83 | 2.38 |
| MACE-MP-0-large | Ni(111) | CO | 1.97 | 2.43 | 0.46 | -2.15 | -0.18 | 1.97 |
| | | $CO_2$ | 2.13 | 4.17 | 2.04 | -0.06 | -0.13 | 0.07 |
| | | H | 1.63 | 1.74 | 0.10 | -0.63 | -0.55 | 0.08 |
| | | $H_2O$ | 2.15 | 3.37 | 1.22 | – | – | – |
| | | O | 1.83 | 1.86 | 0.03 | -0.00 | 4.43 | 4.43 |
| | | OH | 1.91 | 2.21 | 0.30 | -0.33 | 2.28 | 2.61 |
| MACE-MP-0-large | Pd(111) | CO | 2.06 | 2.10 | 0.05 | -2.36 | -1.94 | 0.42 |
| | | $CO_2$ | 2.06 | 2.11 | 0.05 | -0.00 | 0.33 | 0.34 |
| | | H | 1.80 | 1.84 | 0.03 | -0.72 | -0.68 | 0.04 |
| | | $H_2O$ | 2.35 | 2.33 | 0.02 | – | – | – |
| | | O | 1.99 | 2.02 | 0.04 | 0.85 | 1.50 | 0.65 |
| | | OH | 2.14 | 1.96 | 0.18 | 0.38 | 0.84 | 0.46 |
| MACE-MP-0-large-b2 | Ag(111) | CO | 1.92 | 2.05 | 0.13 | -0.40 | -0.82 | 0.42 |
| | | $CO_2$ | 2.18 | 3.41 | 1.23 | -0.11 | -0.28 | 0.18 |
| | | H | 1.86 | 1.91 | 0.05 | 0.07 | 0.03 | 0.04 |
| | | $H_2O$ | 2.37 | 2.53 | 0.16 | – | – | – |
| | | O | 2.02 | 2.17 | 0.15 | 1.84 | 2.26 | 0.42 |
| | | OH | 2.11 | 2.28 | 0.17 | 0.38 | 0.61 | 0.23 |
| MACE-MP-0-large-b2 | Au(111) | CO | 2.16 | 1.93 | 0.23 | -0.49 | -1.35 | 0.86 |
| | | $CO_2$ | 2.18 | 3.14 | 0.96 | -0.10 | -0.49 | 0.40 |
| | | H | 1.87 | 1.96 | 0.09 | 0.04 | -0.09 | 0.13 |
| | | $H_2O$ | 2.37 | 2.99 | 0.61 | – | – | – |
| | | O | 2.03 | 2.16 | 0.13 | 2.32 | 2.75 | 0.43 |
| | | OH | 2.11 | 2.06 | 0.05 | 1.04 | 1.31 | 0.27 |
| MACE-MP-0-large-b2 | Ni(110) | CO | 1.97 | 1.93 | 0.04 | -2.01 | -0.29 | 1.72 |
| | | $CO_2$ | 1.59 | 4.07 | 2.49 | -0.61 | -0.09 | 0.52 |
| | | H | 1.39 | 1.75 | 0.36 | -0.45 | -0.57 | 0.12 |
| | | $H_2O$ | 1.75 | 3.62 | 1.87 | – | – | – |
| | | O | 1.92 | 1.84 | 0.08 | 0.28 | 4.32 | 4.05 |
| | | OH | 2.06 | 1.87 | 0.19 | -0.55 | 1.96 | 2.51 |
| MACE-MP-0-large-b2 | Ni(111) | CO | 1.97 | 1.75 | 0.22 | -2.15 | -0.60 | 1.55 |
| | | $CO_2$ | 2.13 | 4.48 | 2.34 | -0.06 | -0.11 | 0.05 |
| | | H | 1.63 | 1.63 | 0.00 | -0.63 | -0.67 | 0.04 |
| | | $H_2O$ | 2.15 | 3.74 | 1.59 | – | – | – |
| | | O | 1.83 | 1.75 | 0.08 | -0.00 | 5.14 | 5.15 |
| | | OH | 1.91 | 1.95 | 0.04 | -0.33 | 2.53 | 2.85 |
| MACE-MP-0-large-b2 | Pd(111) | CO | 2.06 | 2.07 | 0.01 | -2.36 | -2.01 | 0.35 |
| | | $CO_2$ | 2.06 | 3.52 | 1.46 | -0.00 | -0.18 | 0.17 |
| | | H | 1.80 | 1.80 | 0.00 | -0.72 | -0.58 | 0.14 |
| | | $H_2O$ | 2.35 | 2.33 | 0.02 | – | – | – |
| | | O | 1.99 | 2.03 | 0.04 | 0.85 | 2.65 | 1.79 |
| | | OH | 2.14 | 2.01 | 0.13 | 0.38 | 1.23 | 0.85 |
| MACE-MP-0-medium | Ag(111) | CO | 1.92 | 2.22 | 0.30 | -0.40 | -1.04 | 0.64 |
| | | $CO_2$ | 2.18 | 3.14 | 0.96 | -0.11 | -0.31 | 0.20 |
| | | H | 1.86 | 1.85 | 0.01 | 0.07 | -0.09 | 0.16 |
| | | $H_2O$ | 2.37 | 2.54 | 0.17 | – | – | – |

Continued on next page

**Table S6 – Continued from previous page**

| Model | Surface | Species | $d_{DFT}$ / Å | $d_{Pred}$ / Å | $\|\Delta d\|$ | $E_{DFT}$ / eV | $E_{Pred}$ / eV | $\|\Delta E\|$ |
|---|---|---|---|---|---|---|---|---|
| | | O | 2.02 | 2.15 | 0.13 | 1.84 | 2.39 | 0.55 |
| | | OH | 2.11 | 2.14 | 0.03 | 0.38 | 1.23 | 0.86 |
| MACE-MP-0-medium | Au(111) | CO | 2.16 | 1.96 | 0.20 | -0.49 | -0.91 | 0.42 |
| | | $CO_2$ | 2.18 | 3.57 | 1.39 | -0.10 | -0.16 | 0.06 |
| | | H | 1.87 | 1.92 | 0.05 | 0.04 | -0.02 | 0.06 |
| | | $H_2O$ | 2.37 | 2.75 | 0.37 | – | – | – |
| | | O | 2.03 | 2.13 | 0.10 | 2.32 | 2.65 | 0.33 |
| | | OH | 2.11 | 2.06 | 0.05 | 1.04 | 1.59 | 0.56 |
| MACE-MP-0-medium | Ni(110) | CO | 1.97 | 2.01 | 0.04 | -2.01 | -0.58 | 1.43 |
| | | $CO_2$ | 1.59 | 5.35 | 3.77 | -0.61 | -0.05 | 0.56 |
| | | H | 1.39 | 1.61 | 0.22 | -0.45 | -0.64 | 0.18 |
| | | $H_2O$ | 1.75 | 5.53 | 3.78 | – | – | – |
| | | O | 1.92 | 1.84 | 0.07 | 0.28 | 4.60 | 4.33 |
| | | OH | 2.06 | 1.94 | 0.12 | -0.55 | 2.38 | 2.93 |
| MACE-MP-0-medium | Ni(111) | CO | 1.97 | 1.96 | 0.01 | -2.15 | -0.76 | 1.39 |
| | | $CO_2$ | 2.13 | 5.35 | 3.22 | -0.06 | -0.05 | 0.01 |
| | | H | 1.63 | 1.65 | 0.02 | -0.63 | -0.70 | 0.07 |
| | | $H_2O$ | 2.15 | 5.48 | 3.33 | – | – | – |
| | | O | 1.83 | 1.97 | 0.14 | -0.00 | 4.97 | 4.97 |
| | | OH | 1.91 | 1.98 | 0.07 | -0.33 | 2.81 | 3.13 |
| MACE-MP-0-medium | Pd(111) | CO | 2.06 | 2.06 | 0.01 | -2.36 | -2.07 | 0.29 |
| | | $CO_2$ | 2.06 | 3.94 | 1.88 | -0.00 | -0.24 | 0.24 |
| | | H | 1.80 | 1.78 | 0.02 | -0.72 | -0.94 | 0.22 |
| | | $H_2O$ | 2.35 | 2.40 | 0.06 | – | – | – |
| | | O | 1.99 | 2.06 | 0.08 | 0.85 | 2.06 | 1.21 |
| | | OH | 2.14 | 2.28 | 0.14 | 0.38 | 1.17 | 0.78 |
| MACE-MP-0-medium-b | Ag(111) | CO | 1.92 | 2.17 | 0.25 | -0.40 | -0.81 | 0.41 |
| | | $CO_2$ | 2.18 | 3.37 | 1.19 | -0.11 | -0.33 | 0.23 |
| | | H | 1.86 | 1.91 | 0.05 | 0.07 | 0.05 | 0.02 |
| | | $H_2O$ | 2.37 | 2.47 | 0.10 | – | – | – |
| | | O | 2.02 | 2.14 | 0.12 | 1.84 | 2.09 | 0.25 |
| | | OH | 2.11 | 2.12 | 0.01 | 0.38 | 0.99 | 0.62 |
| MACE-MP-0-medium-b | Au(111) | CO | 2.16 | 1.95 | 0.21 | -0.49 | -1.39 | 0.90 |
| | | $CO_2$ | 2.18 | 3.81 | 1.63 | -0.10 | -0.34 | 0.24 |
| | | H | 1.87 | 1.83 | 0.03 | 0.04 | -0.10 | 0.14 |
| | | $H_2O$ | 2.37 | 2.77 | 0.40 | – | – | – |
| | | O | 2.03 | 2.11 | 0.08 | 2.32 | 2.59 | 0.27 |
| | | OH | 2.11 | 2.06 | 0.06 | 1.04 | 1.30 | 0.26 |
| MACE-MP-0-medium-b | Ni(110) | CO | 1.97 | 1.91 | 0.06 | -2.01 | -1.07 | 0.94 |
| | | $CO_2$ | 1.59 | 5.83 | 4.24 | -0.61 | -0.04 | 0.57 |
| | | H | 1.39 | 1.69 | 0.31 | -0.45 | -0.61 | 0.15 |
| | | $H_2O$ | 1.75 | 5.32 | 3.57 | – | – | – |
| | | O | 1.92 | 1.81 | 0.11 | 0.28 | 4.42 | 4.14 |
| | | OH | 2.06 | 1.83 | 0.23 | -0.55 | 2.61 | 3.16 |
| MACE-MP-0-medium-b | Ni(111) | CO | 1.97 | 1.79 | 0.19 | -2.15 | -1.03 | 1.12 |
| | | $CO_2$ | 2.13 | 5.86 | 3.72 | -0.06 | -0.04 | 0.01 |
| | | H | 1.63 | 1.65 | 0.02 | -0.63 | -0.60 | 0.03 |

Continued on next page

**Table S6 – Continued from previous page**

| Model | Surface | Species | $d_{DFT}$ / Å | $d_{Pred}$ / Å | $\|\Delta d\|$ | $E_{DFT}$ / eV | $E_{Pred}$ / eV | $\|\Delta E\|$ |
|---|---|---|---|---|---|---|---|---|
| | | $H_2O$ | 2.15 | 5.17 | 3.02 | – | – | – |
| | | O | 1.83 | 1.90 | 0.07 | -0.00 | 4.91 | 4.92 |
| | | OH | 1.91 | 1.88 | 0.03 | -0.33 | 3.13 | 3.45 |
| MACE-MP-0-medium-b | Pd(111) | CO | 2.06 | 2.01 | 0.05 | -2.36 | -2.28 | 0.07 |
| | | $CO_2$ | 2.06 | 2.12 | 0.06 | -0.00 | 0.20 | 0.21 |
| | | H | 1.80 | 1.82 | 0.02 | -0.72 | -0.93 | 0.21 |
| | | $H_2O$ | 2.35 | 2.25 | 0.10 | – | – | – |
| | | O | 1.99 | 2.06 | 0.07 | 0.85 | 1.73 | 0.87 |
| | | OH | 2.14 | 1.96 | 0.18 | 0.38 | 0.76 | 0.38 |
| MACE-MP-0-medium-b2 | Ag(111) | CO | 1.92 | 2.16 | 0.24 | -0.40 | -0.73 | 0.33 |
| | | $CO_2$ | 2.18 | 3.38 | 1.20 | -0.11 | -0.39 | 0.28 |
| | | H | 1.86 | 2.02 | 0.16 | 0.07 | -0.28 | 0.35 |
| | | $H_2O$ | 2.37 | 2.38 | 0.00 | – | – | – |
| | | O | 2.02 | 2.16 | 0.14 | 1.84 | 2.12 | 0.28 |
| | | OH | 2.11 | 2.26 | 0.15 | 0.38 | 0.42 | 0.05 |
| MACE-MP-0-medium-b2 | Au(111) | CO | 2.16 | 2.14 | 0.02 | -0.49 | -1.29 | 0.79 |
| | | $CO_2$ | 2.18 | 3.29 | 1.11 | -0.10 | -0.49 | 0.40 |
| | | H | 1.87 | 1.94 | 0.07 | 0.04 | -0.20 | 0.24 |
| | | $H_2O$ | 2.37 | 2.81 | 0.44 | – | – | – |
| | | O | 2.03 | 2.09 | 0.07 | 2.32 | 2.53 | 0.21 |
| | | OH | 2.11 | 2.04 | 0.07 | 1.04 | 1.33 | 0.30 |
| MACE-MP-0-medium-b2 | Ni(110) | CO | 1.97 | 1.91 | 0.06 | -2.01 | -1.15 | 0.86 |
| | | $CO_2$ | 1.59 | 4.49 | 2.91 | -0.61 | -0.15 | 0.45 |
| | | H | 1.39 | 1.66 | 0.27 | -0.45 | -0.49 | 0.04 |
| | | $H_2O$ | 1.75 | 3.94 | 2.19 | – | – | – |
| | | O | 1.92 | 1.82 | 0.10 | 0.28 | 3.27 | 2.99 |
| | | OH | 2.06 | 1.84 | 0.22 | -0.55 | 1.30 | 1.85 |
| MACE-MP-0-medium-b2 | Ni(111) | CO | 1.97 | 1.74 | 0.24 | -2.15 | -1.22 | 0.93 |
| | | $CO_2$ | 2.13 | 4.42 | 2.28 | -0.06 | -0.15 | 0.10 |
| | | H | 1.63 | 1.71 | 0.08 | -0.63 | -0.47 | 0.15 |
| | | $H_2O$ | 2.15 | 3.78 | 1.64 | – | – | – |
| | | O | 1.83 | 1.91 | 0.08 | -0.00 | 3.52 | 3.52 |
| | | OH | 1.91 | 1.88 | 0.03 | -0.33 | 1.70 | 2.03 |
| MACE-MP-0-medium-b2 | Pd(111) | CO | 2.06 | 2.15 | 0.09 | -2.36 | -2.04 | 0.32 |
| | | $CO_2$ | 2.06 | 2.27 | 0.21 | -0.00 | 0.19 | 0.19 |
| | | H | 1.80 | 1.88 | 0.08 | -0.72 | -0.35 | 0.37 |
| | | $H_2O$ | 2.35 | 2.24 | 0.10 | – | – | – |
| | | O | 1.99 | 2.10 | 0.11 | 0.85 | 2.10 | 1.24 |
| | | OH | 2.14 | 2.04 | 0.10 | 0.38 | 1.10 | 0.72 |
| MACE-MP-0-medium-b3 | Ag(111) | CO | 1.92 | 2.09 | 0.17 | -0.40 | -0.83 | 0.43 |
| | | $CO_2$ | 2.18 | 3.53 | 1.35 | -0.11 | -0.22 | 0.11 |
| | | H | 1.86 | 1.97 | 0.11 | 0.07 | -0.07 | 0.14 |
| | | $H_2O$ | 2.37 | 2.50 | 0.12 | – | – | – |
| | | O | 2.02 | 2.15 | 0.13 | 1.84 | 1.91 | 0.07 |
| | | OH | 2.11 | 2.12 | 0.01 | 0.38 | 0.85 | 0.47 |
| MACE-MP-0-medium-b3 | Au(111) | CO | 2.16 | 1.96 | 0.21 | -0.49 | -1.71 | 1.22 |
| | | $CO_2$ | 2.18 | 3.26 | 1.08 | -0.10 | -0.44 | 0.34 |

Continued on next page

**Table S6 – Continued from previous page**

| Model | Surface | Species | $d_{\mathrm{DFT}}$ / Å | $d_{\mathrm{Pred}}$ / Å | $\|\Delta d\|$ | $E_{\mathrm{DFT}}$ / eV | $E_{\mathrm{Pred}}$ / eV | $\|\Delta E\|$ |
|---|---|---|---|---|---|---|---|---|
| | | H | 1.87 | 1.86 | 0.01 | 0.04 | 0.05 | 0.01 |
| | | $H_2O$ | 2.37 | 2.55 | 0.18 | – | – | – |
| | | O | 2.03 | 2.08 | 0.06 | 2.32 | 2.18 | 0.14 |
| | | OH | 2.11 | 2.03 | 0.08 | 1.04 | 1.05 | 0.01 |
| MACE-MP-0-medium-b3 | Ni(110) | CO | 1.97 | 1.93 | 0.04 | -2.01 | -1.18 | 0.83 |
| | | $CO_2$ | 1.59 | 4.80 | 3.22 | -0.61 | -0.08 | 0.53 |
| | | H | 1.39 | 1.61 | 0.22 | -0.45 | -0.45 | 0.01 |
| | | $H_2O$ | 1.75 | 4.04 | 2.29 | – | – | – |
| | | O | 1.92 | 1.94 | 0.02 | 0.28 | 3.34 | 3.06 |
| | | OH | 2.06 | 2.04 | 0.02 | -0.55 | 1.77 | 2.32 |
| MACE-MP-0-medium-b3 | Ni(111) | CO | 1.97 | 1.87 | 0.10 | -2.15 | -1.23 | 0.92 |
| | | $CO_2$ | 2.13 | 4.87 | 2.74 | -0.06 | -0.09 | 0.03 |
| | | H | 1.63 | 1.64 | 0.00 | -0.63 | -0.48 | 0.15 |
| | | $H_2O$ | 2.15 | 3.88 | 1.74 | – | – | – |
| | | O | 1.83 | 1.92 | 0.09 | -0.00 | 3.32 | 3.32 |
| | | OH | 1.91 | 1.88 | 0.03 | -0.33 | 1.92 | 2.25 |
| MACE-MP-0-medium-b3 | Pd(111) | CO | 2.06 | 2.08 | 0.02 | -2.36 | -2.99 | 0.63 |
| | | $CO_2$ | 2.06 | 2.95 | 0.89 | -0.00 | -0.94 | 0.93 |
| | | H | 1.80 | 1.75 | 0.05 | -0.72 | -1.02 | 0.30 |
| | | $H_2O$ | 2.35 | 2.14 | 0.20 | – | – | – |
| | | O | 1.99 | 1.99 | 0.01 | 0.85 | 1.40 | 0.55 |
| | | OH | 2.14 | 1.96 | 0.18 | 0.38 | 0.48 | 0.10 |
| MACE-MP-0-small | Ag(111) | CO | 1.92 | 2.27 | 0.35 | -0.40 | -0.81 | 0.41 |
| | | $CO_2$ | 2.18 | 3.91 | 1.73 | -0.11 | -0.14 | 0.04 |
| | | H | 1.86 | 1.87 | 0.01 | 0.07 | -0.04 | 0.11 |
| | | $H_2O$ | 2.37 | 2.47 | 0.09 | – | – | – |
| | | O | 2.02 | 2.16 | 0.14 | 1.84 | 1.99 | 0.15 |
| | | OH | 2.11 | 2.14 | 0.03 | 0.38 | 1.09 | 0.71 |
| MACE-MP-0-small | Au(111) | CO | 2.16 | 1.98 | 0.18 | -0.49 | -1.04 | 0.55 |
| | | $CO_2$ | 2.18 | 4.17 | 1.99 | -0.10 | -0.16 | 0.06 |
| | | H | 1.87 | 1.61 | 0.25 | 0.04 | 0.03 | 0.02 |
| | | $H_2O$ | 2.37 | 2.54 | 0.17 | – | – | – |
| | | O | 2.03 | 2.14 | 0.12 | 2.32 | 1.50 | 0.81 |
| | | OH | 2.11 | 2.13 | 0.01 | 1.04 | 1.06 | 0.03 |
| MACE-MP-0-small | Ni(110) | CO | 1.97 | 2.19 | 0.22 | -2.01 | -0.35 | 1.66 |
| | | $CO_2$ | 1.59 | 5.52 | 3.94 | -0.61 | -0.04 | 0.57 |
| | | H | 1.39 | 1.61 | 0.22 | -0.45 | -0.93 | 0.48 |
| | | $H_2O$ | 1.75 | 4.48 | 2.73 | – | – | – |
| | | O | 1.92 | 1.79 | 0.13 | 0.28 | 4.66 | 4.38 |
| | | OH | 2.06 | 1.95 | 0.11 | -0.55 | 1.95 | 2.50 |
| MACE-MP-0-small | Ni(111) | CO | 1.97 | 2.09 | 0.11 | -2.15 | -0.27 | 1.88 |
| | | $CO_2$ | 2.13 | 5.58 | 3.45 | -0.06 | -0.06 | 0.01 |
| | | H | 1.63 | 1.68 | 0.04 | -0.63 | -0.83 | 0.20 |
| | | $H_2O$ | 2.15 | 5.18 | 3.03 | – | – | – |
| | | O | 1.83 | 2.10 | 0.27 | -0.00 | 5.79 | 5.79 |
| | | OH | 1.91 | 2.06 | 0.15 | -0.33 | 3.06 | 3.38 |
| MACE-MP-0-small | Pd(111) | CO | 2.06 | 2.03 | 0.03 | -2.36 | -2.18 | 0.17 |

Continued on next page

**Table S6 – Continued from previous page**

| Model | Surface | Species | $d_{DFT}$ / Å | $d_{Pred}$ / Å | $\|\Delta d\|$ | $E_{DFT}$ / eV | $E_{Pred}$ / eV | $\|\Delta E\|$ |
|---|---|---|---|---|---|---|---|---|
| | | $CO_2$ | 2.06 | 2.06 | 0.01 | -0.00 | -0.69 | 0.69 |
| | | H | 1.80 | 1.80 | 0.00 | -0.72 | -0.95 | 0.23 |
| | | $H_2O$ | 2.35 | 2.20 | 0.15 | – | – | – |
| | | O | 1.99 | 2.06 | 0.07 | 0.85 | 1.50 | 0.65 |
| | | OH | 2.14 | 2.08 | 0.07 | 0.38 | 0.17 | 0.21 |
| MACE-MP-0-small-b | Ag(111) | CO | 1.92 | 2.04 | 0.12 | -0.40 | -1.16 | 0.76 |
| | | $CO_2$ | 2.18 | 3.10 | 0.92 | -0.11 | -0.48 | 0.37 |
| | | H | 1.86 | 1.95 | 0.09 | 0.07 | -1.02 | 1.09 |
| | | $H_2O$ | 2.37 | 2.48 | 0.10 | – | – | – |
| | | O | 2.02 | 2.13 | 0.11 | 1.84 | 4.36 | 2.52 |
| | | OH | 2.11 | 2.28 | 0.17 | 0.38 | 1.58 | 1.21 |
| MACE-MP-0-small-b | Au(111) | CO | 2.16 | 1.94 | 0.22 | -0.49 | -1.82 | 1.32 |
| | | $CO_2$ | 2.18 | 3.22 | 1.04 | -0.10 | -0.53 | 0.43 |
| | | H | 1.87 | 1.87 | 0.01 | 0.04 | -1.20 | 1.25 |
| | | $H_2O$ | 2.37 | 2.30 | 0.07 | – | – | – |
| | | O | 2.03 | 2.09 | 0.06 | 2.32 | 4.69 | 2.37 |
| | | OH | 2.11 | 1.99 | 0.12 | 1.04 | 2.41 | 1.37 |
| MACE-MP-0-small-b | Ni(110) | CO | 1.97 | 1.94 | 0.03 | -2.01 | -1.09 | 0.92 |
| | | $CO_2$ | 1.59 | 5.00 | 3.42 | -0.61 | -0.09 | 0.52 |
| | | H | 1.39 | 1.57 | 0.18 | -0.45 | -1.34 | 0.89 |
| | | $H_2O$ | 1.75 | 5.93 | 4.18 | – | – | – |
| | | O | 1.92 | 1.81 | 0.11 | 0.28 | 6.49 | 6.21 |
| | | OH | 2.06 | 1.86 | 0.20 | -0.55 | 3.70 | 4.25 |
| MACE-MP-0-small-b | Ni(111) | CO | 1.97 | 1.92 | 0.05 | -2.15 | -1.32 | 0.83 |
| | | $CO_2$ | 2.13 | 4.97 | 2.84 | -0.06 | -0.07 | 0.01 |
| | | H | 1.63 | 1.66 | 0.03 | -0.63 | -1.40 | 0.77 |
| | | $H_2O$ | 2.15 | 5.92 | 3.77 | – | – | – |
| | | O | 1.83 | 1.91 | 0.08 | -0.00 | 6.89 | 6.89 |
| | | OH | 1.91 | 1.92 | 0.01 | -0.33 | 4.06 | 4.39 |
| MACE-MP-0-small-b | Pd(111) | CO | 2.06 | 2.08 | 0.02 | -2.36 | -3.27 | 0.91 |
| | | $CO_2$ | 2.06 | 2.15 | 0.09 | -0.00 | -0.49 | 0.48 |
| | | H | 1.80 | 1.80 | 0.00 | -0.72 | -2.04 | 1.32 |
| | | $H_2O$ | 2.35 | 2.41 | 0.07 | – | – | – |
| | | O | 1.99 | 2.01 | 0.02 | 0.85 | 3.69 | 2.83 |
| | | OH | 2.14 | 1.93 | 0.21 | 0.38 | 1.83 | 1.44 |
| MACE-MP-0-small-b2 | Ag(111) | CO | 1.92 | 2.19 | 0.27 | -0.40 | -0.83 | 0.43 |
| | | $CO_2$ | 2.18 | 3.49 | 1.31 | -0.11 | -0.28 | 0.17 |
| | | H | 1.86 | 1.92 | 0.06 | 0.07 | -0.28 | 0.35 |
| | | $H_2O$ | 2.37 | 2.46 | 0.08 | – | – | – |
| | | O | 2.02 | 2.15 | 0.13 | 1.84 | 2.19 | 0.35 |
| | | OH | 2.11 | 2.12 | 0.01 | 0.38 | 1.07 | 0.69 |
| MACE-MP-0-small-b2 | Au(111) | CO | 2.16 | 1.92 | 0.25 | -0.49 | -1.11 | 0.62 |
| | | $CO_2$ | 2.18 | 3.32 | 1.14 | -0.10 | -0.37 | 0.27 |
| | | H | 1.87 | 1.95 | 0.09 | 0.04 | -0.28 | 0.32 |
| | | $H_2O$ | 2.37 | 2.49 | 0.11 | – | – | – |
| | | O | 2.03 | 2.12 | 0.09 | 2.32 | 2.54 | 0.22 |
| | | OH | 2.11 | 2.04 | 0.07 | 1.04 | 1.17 | 0.13 |

Continued on next page

**Table S6 – Continued from previous page**

| Model | Surface | Species | $d_{\mathrm{DFT}}$ / Å | $d_{\mathrm{Pred}}$ / Å | $\lvert\Delta d\rvert$ | $E_{\mathrm{DFT}}$ / eV | $E_{\mathrm{Pred}}$ / eV | $\lvert\Delta E\rvert$ |
|---|---|---|---|---|---|---|---|---|
| MACE-MP-0-small-b2 | Ni(110) | CO | 1.97 | 1.91 | 0.06 | -2.01 | -1.11 | 0.90 |
| | | $CO_2$ | 1.59 | 3.98 | 2.40 | -0.61 | -0.16 | 0.45 |
| | | H | 1.39 | 1.64 | 0.26 | -0.45 | -0.64 | 0.19 |
| | | $H_2O$ | 1.75 | 2.31 | 0.56 | – | – | – |
| | | O | 1.92 | 1.84 | 0.08 | 0.28 | 3.65 | 3.37 |
| | | OH | 2.06 | 2.24 | 0.18 | -0.55 | 1.70 | 2.25 |
| MACE-MP-0-small-b2 | Ni(111) | CO | 1.97 | 1.87 | 0.10 | -2.15 | -1.14 | 1.01 |
| | | $CO_2$ | 2.13 | 4.21 | 2.07 | -0.06 | -0.17 | 0.11 |
| | | H | 1.63 | 1.74 | 0.10 | -0.63 | -0.68 | 0.05 |
| | | $H_2O$ | 2.15 | 2.36 | 0.21 | – | – | – |
| | | O | 1.83 | 1.92 | 0.09 | -0.00 | 4.03 | 4.03 |
| | | OH | 1.91 | 1.95 | 0.04 | -0.33 | 1.91 | 2.24 |
| MACE-MP-0-small-b2 | Pd(111) | CO | 2.06 | 1.99 | 0.07 | -2.36 | -1.63 | 0.72 |
| | | $CO_2$ | 2.06 | 3.15 | 1.09 | -0.00 | -0.31 | 0.31 |
| | | H | 1.80 | 1.82 | 0.02 | -0.72 | -0.79 | 0.07 |
| | | $H_2O$ | 2.35 | 2.37 | 0.02 | – | – | – |
| | | O | 1.99 | 2.01 | 0.02 | 0.85 | 2.28 | 1.42 |
| | | OH | 2.14 | 2.03 | 0.11 | 0.38 | 1.07 | 0.69 |
| MACE-MPA-0-medium | Ag(111) | CO | 1.92 | 2.10 | 0.18 | -0.40 | -0.71 | 0.31 |
| | | $CO_2$ | 2.18 | 3.69 | 1.51 | -0.11 | -0.33 | 0.23 |
| | | H | 1.86 | 1.94 | 0.08 | 0.07 | -0.03 | 0.10 |
| | | $H_2O$ | 2.37 | 2.53 | 0.15 | – | – | – |
| | | O | 2.02 | 2.13 | 0.11 | 1.84 | 2.01 | 0.17 |
| | | OH | 2.11 | 2.10 | 0.01 | 0.38 | 0.91 | 0.53 |
| MACE-MPA-0-medium | Au(111) | CO | 2.16 | 2.03 | 0.13 | -0.49 | -0.85 | 0.35 |
| | | $CO_2$ | 2.18 | 3.48 | 1.30 | -0.10 | -0.30 | 0.20 |
| | | H | 1.87 | 1.90 | 0.03 | 0.04 | -0.07 | 0.11 |
| | | $H_2O$ | 2.37 | 2.53 | 0.16 | – | – | – |
| | | O | 2.03 | 2.13 | 0.11 | 2.32 | 2.28 | 0.04 |
| | | OH | 2.11 | 2.06 | 0.05 | 1.04 | 1.08 | 0.05 |
| MACE-MPA-0-medium | Ni(110) | CO | 1.97 | 1.95 | 0.02 | -2.01 | 0.51 | 2.53 |
| | | $CO_2$ | 1.59 | 5.95 | 4.37 | -0.61 | -0.04 | 0.57 |
| | | H | 1.39 | 1.69 | 0.30 | -0.45 | -0.86 | 0.40 |
| | | $H_2O$ | 1.75 | 5.16 | 3.41 | – | – | – |
| | | O | 1.92 | 5.97 | 4.05 | 0.28 | 5.71 | 5.44 |
| | | OH | 2.06 | 5.12 | 3.06 | -0.55 | 2.86 | 3.41 |
| MACE-MPA-0-medium | Ni(111) | CO | 1.97 | 1.84 | 0.13 | -2.15 | 0.79 | 2.94 |
| | | $CO_2$ | 2.13 | 5.91 | 3.78 | -0.06 | -0.06 | 0.00 |
| | | H | 1.63 | 1.68 | 0.05 | -0.63 | -0.91 | 0.28 |
| | | $H_2O$ | 2.15 | 4.87 | 2.73 | – | – | – |
| | | O | 1.83 | 6.03 | 4.20 | -0.00 | 5.70 | 5.70 |
| | | OH | 1.91 | 6.49 | 4.58 | -0.33 | 2.88 | 3.21 |
| MACE-MPA-0-medium | Pd(111) | CO | 2.06 | 1.87 | 0.19 | -2.36 | -2.28 | 0.08 |
| | | $CO_2$ | 2.06 | 2.42 | 0.36 | -0.00 | -0.43 | 0.43 |
| | | H | 1.80 | 1.82 | 0.01 | -0.72 | -0.78 | 0.06 |
| | | $H_2O$ | 2.35 | 2.27 | 0.08 | – | – | – |
| | | O | 1.99 | 2.02 | 0.03 | 0.85 | 1.47 | 0.61 |

Continued on next page

**Table S6 – Continued from previous page**

| Model | Surface | Species | $d_{DFT}$ / Å | $d_{Pred}$ / Å | $\|\Delta d\|$ | $E_{DFT}$ / eV | $E_{Pred}$ / eV | $\|\Delta E\|$ |
|---|---|---|---|---|---|---|---|---|
| | | OH | 2.14 | 2.03 | 0.11 | 0.38 | 0.84 | 0.45 |
| MACE-OMat-medium | Ag(111) | CO | 1.92 | 2.12 | 0.20 | -0.40 | -1.19 | 0.79 |
| | | $CO_2$ | 2.18 | 3.16 | 0.98 | -0.11 | -0.49 | 0.39 |
| | | H | 1.86 | 1.91 | 0.05 | 0.07 | -0.08 | 0.15 |
| | | $H_2O$ | 2.37 | 2.50 | 0.13 | – | – | – |
| | | O | 2.02 | 2.14 | 0.12 | 1.84 | 1.95 | 0.11 |
| | | OH | 2.11 | 2.12 | 0.01 | 0.38 | 0.81 | 0.44 |
| MACE-OMat-medium | Au(111) | CO | 2.16 | 2.00 | 0.16 | -0.49 | -1.27 | 0.78 |
| | | $CO_2$ | 2.18 | 3.12 | 0.94 | -0.10 | -0.60 | 0.50 |
| | | H | 1.87 | 1.89 | 0.02 | 0.04 | -0.08 | 0.12 |
| | | $H_2O$ | 2.37 | 2.72 | 0.35 | – | – | – |
| | | O | 2.03 | 2.14 | 0.11 | 2.32 | 2.30 | 0.02 |
| | | OH | 2.11 | 2.10 | 0.02 | 1.04 | 1.06 | 0.03 |
| MACE-OMat-medium | Ni(110) | CO | 1.97 | 4.80 | 2.83 | -2.01 | -0.16 | 1.85 |
| | | $CO_2$ | 1.59 | 4.84 | 3.25 | -0.61 | 5.60 | 6.21 |
| | | H | 1.39 | 1.62 | 0.23 | -0.45 | -0.66 | 0.20 |
| | | $H_2O$ | 1.75 | 5.13 | 3.38 | – | – | – |
| | | O | 1.92 | 6.19 | 4.28 | 0.28 | 5.79 | 5.51 |
| | | OH | 2.06 | 6.08 | 4.02 | -0.55 | 2.58 | 3.13 |
| MACE-OMat-medium | Ni(111) | CO | 1.97 | 4.46 | 2.49 | -2.15 | -0.18 | 1.97 |
| | | $CO_2$ | 2.13 | 5.99 | 3.85 | -0.06 | -0.04 | 0.01 |
| | | H | 1.63 | 1.70 | 0.07 | -0.63 | -0.76 | 0.14 |
| | | $H_2O$ | 2.15 | 5.40 | 3.26 | – | – | – |
| | | O | 1.83 | 5.99 | 4.16 | -0.00 | 5.79 | 5.79 |
| | | OH | 1.91 | 5.97 | 4.06 | -0.33 | 2.57 | 2.90 |
| MACE-OMat-medium | Pd(111) | CO | 2.06 | 2.04 | 0.02 | -2.36 | -3.12 | 0.76 |
| | | $CO_2$ | 2.06 | 2.13 | 0.07 | -0.00 | -0.55 | 0.54 |
| | | H | 1.80 | 1.79 | 0.01 | -0.72 | -0.77 | 0.05 |
| | | $H_2O$ | 2.35 | 2.49 | 0.14 | – | – | – |
| | | O | 1.99 | 2.01 | 0.02 | 0.85 | 1.37 | 0.52 |
| | | OH | 2.14 | 2.00 | 0.15 | 0.38 | 0.62 | 0.24 |

**Table S7:** Performance metrics for OCP models

| Model | Surface | Species | $d_{DFT}$ / Å | $d_{Pred}$ / Å | $\|\Delta d\|$ | $E_{DFT}$ / eV | $E_{Pred}$ / eV | $\|\Delta E\|$ |
|---|---|---|---|---|---|---|---|---|
| Equiformerv2-153M | Ag(111) | CO | 1.92 | 2.19 | 0.27 | -0.40 | -0.06 | 0.33 |
| | | $CO_2$ | 2.18 | 4.59 | 2.41 | -0.11 | -0.48 | 0.38 |
| | | H | 1.86 | 1.94 | 0.08 | 0.07 | 0.33 | 0.26 |
| | | $H_2O$ | 2.37 | 2.91 | 0.53 | – | – | – |
| | | O | 2.02 | 2.17 | 0.15 | 1.84 | 2.24 | 0.40 |
| | | OH | 2.11 | 2.12 | 0.01 | 0.38 | 1.19 | 0.82 |
| Equiformerv2-153M | Au(111) | CO | 2.16 | 2.04 | 0.13 | -0.49 | -0.09 | 0.40 |
| | | $CO_2$ | 2.18 | 4.55 | 2.37 | -0.10 | -0.45 | 0.35 |
| | | H | 1.87 | 1.89 | 0.03 | 0.04 | 0.31 | 0.26 |
| | | $H_2O$ | 2.37 | 3.49 | 1.12 | – | – | – |
| | | O | 2.03 | 2.15 | 0.13 | 2.32 | 2.73 | 0.42 |

Continued on next page

Table S7 – Continued from previous page

| Model | Surface | Species | $d_{DFT}$ / Å | $d_{Pred}$ / Å | $\|\Delta d\|$ | $E_{DFT}$ / eV | $E_{Pred}$ / eV | $\|\Delta E\|$ |
|---|---|---|---|---|---|---|---|---|
| | | OH | 2.11 | 2.09 | 0.02 | 1.04 | 1.51 | 0.47 |
| Equiformerv2-153M | Ni(110) | CO | 1.97 | 1.86 | 0.11 | -2.01 | -1.43 | 0.58 |
| | | $CO_2$ | 1.59 | 1.90 | 0.31 | -0.61 | -0.64 | 0.04 |
| | | H | 1.39 | 1.62 | 0.24 | -0.45 | -0.38 | 0.08 |
| | | $H_2O$ | 1.75 | 2.51 | 0.76 | – | – | – |
| | | O | 1.92 | 1.75 | 0.17 | 0.28 | 0.35 | 0.07 |
| | | OH | 2.06 | 1.97 | 0.09 | -0.55 | 0.04 | 0.59 |
| Equiformerv2-153M | Ni(111) | CO | 1.97 | 1.94 | 0.03 | -2.15 | -1.80 | 0.35 |
| | | $CO_2$ | 2.13 | 4.56 | 2.42 | -0.06 | -0.44 | 0.39 |
| | | H | 1.63 | 1.70 | 0.06 | -0.63 | -0.48 | 0.15 |
| | | $H_2O$ | 2.15 | 2.24 | 0.09 | – | – | – |
| | | O | 1.83 | 1.83 | 0.00 | -0.00 | 0.19 | 0.19 |
| | | OH | 1.91 | 1.82 | 0.09 | -0.33 | 0.61 | 0.93 |
| Equiformerv2-153M | Pd(111) | CO | 2.06 | 1.87 | 0.19 | -2.36 | -1.17 | 1.18 |
| | | $CO_2$ | 2.06 | 4.27 | 2.21 | -0.00 | -0.43 | 0.43 |
| | | H | 1.80 | 1.81 | 0.00 | -0.72 | -0.40 | 0.32 |
| | | $H_2O$ | 2.35 | 2.52 | 0.17 | – | – | – |
| | | O | 1.99 | 2.00 | 0.02 | 0.85 | 1.49 | 0.63 |
| | | OH | 2.14 | 1.98 | 0.16 | 0.38 | 1.03 | 0.65 |
| Equiformerv2-31M | Ag(111) | CO | 1.92 | 2.21 | 0.29 | -0.40 | -0.06 | 0.34 |
| | | $CO_2$ | 2.18 | 4.13 | 1.95 | -0.11 | -0.51 | 0.41 |
| | | H | 1.86 | 1.94 | 0.08 | 0.07 | 0.28 | 0.21 |
| | | $H_2O$ | 2.37 | 2.97 | 0.59 | – | – | – |
| | | O | 2.02 | 2.16 | 0.14 | 1.84 | 2.19 | 0.35 |
| | | OH | 2.11 | 2.12 | 0.01 | 0.38 | 1.18 | 0.80 |
| Equiformerv2-31M | Au(111) | CO | 2.16 | 2.03 | 0.13 | -0.49 | -0.09 | 0.40 |
| | | $CO_2$ | 2.18 | 4.30 | 2.12 | -0.10 | -0.47 | 0.37 |
| | | H | 1.87 | 1.90 | 0.03 | 0.04 | 0.26 | 0.22 |
| | | $H_2O$ | 2.37 | 3.63 | 1.26 | – | – | – |
| | | O | 2.03 | 2.15 | 0.12 | 2.32 | 2.69 | 0.37 |
| | | OH | 2.11 | 2.09 | 0.02 | 1.04 | 1.50 | 0.46 |
| Equiformerv2-31M | Ni(110) | CO | 1.97 | 1.86 | 0.11 | -2.01 | -1.44 | 0.57 |
| | | $CO_2$ | 1.59 | 1.90 | 0.32 | -0.61 | -0.66 | 0.05 |
| | | H | 1.39 | 1.62 | 0.23 | -0.45 | -0.40 | 0.06 |
| | | $H_2O$ | 1.75 | 2.52 | 0.77 | – | – | – |
| | | O | 1.92 | 1.75 | 0.17 | 0.28 | 0.33 | 0.05 |
| | | OH | 2.06 | 1.97 | 0.09 | -0.55 | 0.02 | 0.57 |
| Equiformerv2-31M | Ni(111) | CO | 1.97 | 1.95 | 0.02 | -2.15 | -1.79 | 0.36 |
| | | $CO_2$ | 2.13 | 4.42 | 2.29 | -0.06 | -0.48 | 0.43 |
| | | H | 1.63 | 1.71 | 0.08 | -0.63 | -0.49 | 0.14 |
| | | $H_2O$ | 2.15 | 2.24 | 0.09 | – | – | – |
| | | O | 1.83 | 1.83 | 0.00 | -0.00 | 0.20 | 0.20 |
| | | OH | 1.91 | 1.82 | 0.09 | -0.33 | 0.60 | 0.92 |
| Equiformerv2-31M | Pd(111) | CO | 2.06 | 1.87 | 0.19 | -2.36 | -1.18 | 1.18 |
| | | $CO_2$ | 2.06 | 4.50 | 2.44 | -0.00 | -0.43 | 0.43 |
| | | H | 1.80 | 1.82 | 0.01 | -0.72 | -0.39 | 0.33 |
| | | $H_2O$ | 2.35 | 2.60 | 0.26 | – | – | – |

Continued on next page

**Table S7 – Continued from previous page**

| Model | Surface | Species | $d_{\mathrm{DFT}}$ / Å | $d_{\mathrm{Pred}}$ / Å | $\lvert\Delta d\rvert$ | $E_{\mathrm{DFT}}$ / eV | $E_{\mathrm{Pred}}$ / eV | $\lvert\Delta E\rvert$ |
|---|---|---|---|---|---|---|---|---|
| | | O | 1.99 | 2.00 | 0.02 | 0.85 | 1.47 | 0.61 |
| | | OH | 2.14 | 1.98 | 0.16 | 0.38 | 1.04 | 0.66 |
| Equiformerv2-83M | Ag(111) | CO | 1.92 | 2.17 | 0.25 | -0.40 | -0.18 | 0.22 |
| | | $CO_2$ | 2.18 | 4.19 | 2.01 | -0.11 | 0.04 | 0.15 |
| | | H | 1.86 | 1.92 | 0.05 | 0.07 | 0.34 | 0.27 |
| | | $H_2O$ | 2.37 | 2.84 | 0.47 | – | – | – |
| | | O | 2.02 | 2.16 | 0.14 | 1.84 | 2.18 | 0.34 |
| | | OH | 2.11 | 2.12 | 0.01 | 0.38 | 1.19 | 0.81 |
| Equiformerv2-83M | Au(111) | CO | 2.16 | 2.03 | 0.13 | -0.49 | -0.21 | 0.28 |
| | | $CO_2$ | 2.18 | 4.29 | 2.11 | -0.10 | 0.17 | 0.27 |
| | | H | 1.87 | 1.90 | 0.03 | 0.04 | 0.29 | 0.25 |
| | | $H_2O$ | 2.37 | 3.42 | 1.05 | – | – | – |
| | | O | 2.03 | 2.15 | 0.13 | 2.32 | 2.63 | 0.31 |
| | | OH | 2.11 | 2.09 | 0.02 | 1.04 | 1.50 | 0.46 |
| Equiformerv2-83M | Ni(110) | CO | 1.97 | 1.86 | 0.11 | -2.01 | -1.51 | 0.51 |
| | | $CO_2$ | 1.59 | 1.90 | 0.31 | -0.61 | -0.12 | 0.49 |
| | | H | 1.39 | 1.62 | 0.23 | -0.45 | -0.43 | 0.03 |
| | | $H_2O$ | 1.75 | 2.51 | 0.76 | – | – | – |
| | | O | 1.92 | 1.75 | 0.17 | 0.28 | 0.35 | 0.07 |
| | | OH | 2.06 | 1.97 | 0.09 | -0.55 | -0.00 | 0.55 |
| Equiformerv2-83M | Ni(111) | CO | 1.97 | 1.88 | 0.09 | -2.15 | -1.69 | 0.46 |
| | | $CO_2$ | 2.13 | 2.14 | 0.01 | -0.06 | 1.01 | 1.06 |
| | | H | 1.63 | 1.70 | 0.07 | -0.63 | -0.49 | 0.14 |
| | | $H_2O$ | 2.15 | 2.24 | 0.09 | – | – | – |
| | | O | 1.83 | 1.83 | 0.00 | -0.00 | 0.20 | 0.20 |
| | | OH | 1.91 | 1.82 | 0.09 | -0.33 | 0.60 | 0.92 |
| Equiformerv2-83M | Pd(111) | CO | 2.06 | 1.87 | 0.19 | -2.36 | -1.16 | 1.19 |
| | | $CO_2$ | 2.06 | 4.23 | 2.17 | -0.00 | 0.19 | 0.19 |
| | | H | 1.80 | 1.81 | 0.01 | -0.72 | -0.41 | 0.31 |
| | | $H_2O$ | 2.35 | 2.55 | 0.20 | – | – | – |
| | | O | 1.99 | 1.99 | 0.01 | 0.85 | 1.43 | 0.58 |
| | | OH | 2.14 | 1.98 | 0.16 | 0.38 | 1.00 | 0.61 |

**Table S8:** Performance metrics for ORB models

| Model | Surface | Species | $d_{\mathrm{DFT}}$ / Å | $d_{\mathrm{Pred}}$ / Å | $\lvert\Delta d\rvert$ | $E_{\mathrm{DFT}}$ / eV | $E_{\mathrm{Pred}}$ / eV | $\lvert\Delta E\rvert$ |
|---|---|---|---|---|---|---|---|---|
| Orb-D3-SM-V2 | Ag(111) | CO | 1.92 | 2.11 | 0.19 | -0.40 | -0.68 | 0.28 |
| | | $CO_2$ | 2.18 | 3.92 | 1.74 | -0.11 | -0.31 | 0.21 |
| | | H | 1.86 | 1.93 | 0.07 | 0.07 | 0.16 | 0.09 |
| | | $H_2O$ | 2.37 | 3.09 | 0.71 | – | – | – |
| | | O | 2.02 | 2.17 | 0.14 | 1.84 | 2.48 | 0.64 |
| | | OH | 2.11 | 2.29 | 0.18 | 0.38 | 0.76 | 0.38 |
| Orb-D3-SM-V2 | Au(111) | CO | 2.16 | 1.98 | 0.18 | -0.49 | -0.95 | 0.45 |
| | | $CO_2$ | 2.18 | 3.81 | 1.63 | -0.10 | -0.36 | 0.27 |
| | | H | 1.87 | 1.88 | 0.02 | 0.04 | 0.03 | 0.02 |
| | | $H_2O$ | 2.37 | 2.84 | 0.47 | – | – | – |

Continued on next page

**Table S8 – Continued from previous page**

| Model | Surface | Species | $d_{DFT}$ / Å | $d_{Pred}$ / Å | $\|\Delta d\|$ | $E_{DFT}$ / eV | $E_{Pred}$ / eV | $\|\Delta E\|$ |
|---|---|---|---|---|---|---|---|---|
| | | O | 2.03 | 2.16 | 0.13 | 2.32 | 2.58 | 0.26 |
| | | OH | 2.11 | 2.28 | 0.17 | 1.04 | 1.16 | 0.13 |
| Orb-D3-SM-V2 | Ni(110) | CO | 1.97 | 1.84 | 0.13 | -2.01 | 66.37 | 68.38 |
| | | $CO_2$ | 1.59 | 2.03 | 0.45 | -0.61 | 113.89 | 114.49 |
| | | H | 1.39 | 1.61 | 0.23 | -0.45 | -1.32 | 0.87 |
| | | $H_2O$ | 1.75 | 2.07 | 0.33 | – | – | – |
| | | O | 1.92 | 1.76 | 0.16 | 0.28 | 74.71 | 74.43 |
| | | OH | 2.06 | 2.06 | 0.00 | -0.55 | 97.67 | 98.22 |
| Orb-D3-SM-V2 | Ni(111) | CO | 1.97 | 1.71 | 0.26 | -2.15 | -1.08 | 1.07 |
| | | $CO_2$ | 2.13 | 3.28 | 1.15 | -0.06 | 37.39 | 37.44 |
| | | H | 1.63 | 1.68 | 0.05 | -0.63 | -0.91 | 0.28 |
| | | $H_2O$ | 2.15 | 2.00 | 0.14 | – | – | – |
| | | O | 1.83 | 1.86 | 0.03 | -0.00 | 55.44 | 55.44 |
| | | OH | 1.91 | 1.94 | 0.03 | -0.33 | 65.43 | 65.75 |
| Orb-D3-SM-V2 | Pd(111) | CO | 2.06 | 2.02 | 0.04 | -2.36 | -2.09 | 0.26 |
| | | $CO_2$ | 2.06 | 3.17 | 1.11 | -0.00 | -0.27 | 0.26 |
| | | H | 1.80 | 1.82 | 0.01 | -0.72 | -0.60 | 0.12 |
| | | $H_2O$ | 2.35 | 2.44 | 0.09 | – | – | – |
| | | O | 1.99 | 2.01 | 0.03 | 0.85 | 1.41 | 0.55 |
| | | OH | 2.14 | 2.15 | 0.01 | 0.38 | 0.34 | 0.04 |
| Orb-D3-V2 | Ag(111) | CO | 1.92 | 2.11 | 0.19 | -0.40 | -0.57 | 0.17 |
| | | $CO_2$ | 2.18 | 3.50 | 1.32 | -0.11 | -0.12 | 0.02 |
| | | H | 1.86 | 1.92 | 0.05 | 0.07 | 0.09 | 0.02 |
| | | $H_2O$ | 2.37 | 2.55 | 0.18 | – | – | – |
| | | O | 2.02 | 2.18 | 0.16 | 1.84 | 2.05 | 0.21 |
| | | OH | 2.11 | 2.28 | 0.17 | 0.38 | 0.40 | 0.02 |
| Orb-D3-V2 | Au(111) | CO | 2.16 | 1.97 | 0.19 | -0.49 | -0.94 | 0.45 |
| | | $CO_2$ | 2.18 | 3.42 | 1.24 | -0.10 | -0.21 | 0.11 |
| | | H | 1.87 | 1.89 | 0.02 | 0.04 | 0.09 | 0.05 |
| | | $H_2O$ | 2.37 | 2.71 | 0.33 | – | – | – |
| | | O | 2.03 | 2.14 | 0.11 | 2.32 | 2.35 | 0.03 |
| | | OH | 2.11 | 2.28 | 0.17 | 1.04 | 0.88 | 0.15 |
| Orb-D3-V2 | Ni(110) | CO | 1.97 | 1.92 | 0.05 | -2.01 | 26.13 | 28.14 |
| | | $CO_2$ | 1.59 | 2.02 | 0.43 | -0.61 | 128.01 | 128.62 |
| | | H | 1.39 | 1.66 | 0.28 | -0.45 | -0.05 | 0.40 |
| | | $H_2O$ | 1.75 | 2.61 | 0.86 | – | – | – |
| | | O | 1.92 | 1.67 | 0.25 | 0.28 | 75.22 | 74.95 |
| | | OH | 2.06 | 1.99 | 0.07 | -0.55 | 85.18 | 85.73 |
| Orb-D3-V2 | Ni(111) | CO | 1.97 | 1.77 | 0.20 | -2.15 | 15.64 | 17.79 |
| | | $CO_2$ | 2.13 | 1.98 | 0.15 | -0.06 | 93.22 | 93.28 |
| | | H | 1.63 | 1.68 | 0.04 | -0.63 | 0.00 | 0.63 |
| | | $H_2O$ | 2.15 | 2.04 | 0.10 | – | – | – |
| | | O | 1.83 | 1.72 | 0.11 | -0.00 | 66.77 | 66.77 |
| | | OH | 1.91 | 1.75 | 0.17 | -0.33 | 57.90 | 58.22 |
| Orb-D3-V2 | Pd(111) | CO | 2.06 | 1.87 | 0.19 | -2.36 | -1.85 | 0.51 |
| | | $CO_2$ | 2.06 | 3.21 | 1.15 | -0.00 | -0.18 | 0.18 |
| | | H | 1.80 | 1.80 | 0.01 | -0.72 | -0.66 | 0.06 |

Continued on next page

**Table S8 – Continued from previous page**

| Model | Surface | Species | $d_{DFT}$ / Å | $d_{Pred}$ / Å | $\|\Delta d\|$ | $E_{DFT}$ / eV | $E_{Pred}$ / eV | $\|\Delta E\|$ |
|---|---|---|---|---|---|---|---|---|
| | | $H_2O$ | 2.35 | 2.37 | 0.02 | – | – | – |
| | | O | 1.99 | 2.02 | 0.03 | 0.85 | 1.12 | 0.27 |
| | | OH | 2.14 | 2.11 | 0.03 | 0.38 | 0.43 | 0.04 |
| Orb-D3-XS-V2 | Ag(111) | CO | 1.92 | 2.05 | 0.13 | -0.40 | -0.91 | 0.51 |
| | | $CO_2$ | 2.18 | 5.18 | 3.00 | -0.11 | 0.20 | 0.31 |
| | | H | 1.86 | 1.93 | 0.07 | 0.07 | -0.22 | 0.29 |
| | | $H_2O$ | 2.37 | 3.02 | 0.65 | – | – | – |
| | | O | 2.02 | 2.11 | 0.09 | 1.84 | 2.13 | 0.29 |
| | | OH | 2.11 | 2.27 | 0.16 | 0.38 | 0.50 | 0.12 |
| Orb-D3-XS-V2 | Au(111) | CO | 2.16 | 1.95 | 0.22 | -0.49 | -1.09 | 0.60 |
| | | $CO_2$ | 2.18 | 3.96 | 1.78 | -0.10 | -0.06 | 0.04 |
| | | H | 1.87 | 1.89 | 0.03 | 0.04 | 0.03 | 0.02 |
| | | $H_2O$ | 2.37 | 2.97 | 0.60 | – | – | – |
| | | O | 2.03 | 2.18 | 0.15 | 2.32 | 2.25 | 0.07 |
| | | OH | 2.11 | 2.17 | 0.05 | 1.04 | 1.34 | 0.30 |
| Orb-D3-XS-V2 | Ni(110) | CO | 1.97 | 1.72 | 0.25 | -2.01 | 6.93 | 8.94 |
| | | $CO_2$ | 1.59 | 2.29 | 0.70 | -0.61 | 62.39 | 62.99 |
| | | H | 1.39 | 1.72 | 0.34 | -0.45 | 0.46 | 0.92 |
| | | $H_2O$ | 1.75 | 4.47 | 2.72 | – | – | – |
| | | O | 1.92 | 1.75 | 0.17 | 0.28 | 73.35 | 73.07 |
| | | OH | 2.06 | 1.84 | 0.22 | -0.55 | 27.79 | 28.34 |
| Orb-D3-XS-V2 | Ni(111) | CO | 1.97 | 1.81 | 0.16 | -2.15 | 30.68 | 32.83 |
| | | $CO_2$ | 2.13 | 2.00 | 0.14 | -0.06 | 49.95 | 50.01 |
| | | H | 1.63 | 1.68 | 0.05 | -0.63 | -0.36 | 0.27 |
| | | $H_2O$ | 2.15 | 2.23 | 0.08 | – | – | – |
| | | O | 1.83 | 1.66 | 0.17 | -0.00 | 53.73 | 53.73 |
| | | OH | 1.91 | 1.79 | 0.12 | -0.33 | 52.61 | 52.94 |
| Orb-D3-XS-V2 | Pd(111) | CO | 2.06 | 1.87 | 0.18 | -2.36 | -2.28 | 0.08 |
| | | $CO_2$ | 2.06 | 3.83 | 1.77 | -0.00 | -0.08 | 0.07 |
| | | H | 1.80 | 1.80 | 0.00 | -0.72 | -0.39 | 0.33 |
| | | $H_2O$ | 2.35 | 3.03 | 0.68 | – | – | – |
| | | O | 1.99 | 2.02 | 0.03 | 0.85 | 1.20 | 0.34 |
| | | OH | 2.14 | 2.01 | 0.13 | 0.38 | 1.15 | 0.76 |
| Orb-MPTrj-V2 | Ag(111) | CO | 1.92 | 2.24 | 0.32 | -0.40 | -0.53 | 0.13 |
| | | $CO_2$ | 2.18 | 5.00 | 2.83 | -0.11 | 0.73 | 0.83 |
| | | H | 1.86 | 1.92 | 0.06 | 0.07 | -0.34 | 0.41 |
| | | $H_2O$ | 2.37 | 2.50 | 0.13 | – | – | – |
| | | O | 2.02 | 2.15 | 0.13 | 1.84 | 2.02 | 0.18 |
| | | OH | 2.11 | 2.25 | 0.14 | 0.38 | 0.17 | 0.21 |
| Orb-MPTrj-V2 | Au(111) | CO | 2.16 | 1.94 | 0.23 | -0.49 | -1.06 | 0.57 |
| | | $CO_2$ | 2.18 | 3.48 | 1.30 | -0.10 | 0.09 | 0.19 |
| | | H | 1.87 | 1.92 | 0.05 | 0.04 | -0.37 | 0.41 |
| | | $H_2O$ | 2.37 | 3.02 | 0.64 | – | – | – |
| | | O | 2.03 | 2.07 | 0.04 | 2.32 | 1.60 | 0.71 |
| | | OH | 2.11 | 2.03 | 0.08 | 1.04 | 1.20 | 0.17 |
| Orb-MPTrj-V2 | Ni(110) | CO | 1.97 | 1.81 | 0.16 | -2.01 | -1.24 | 0.77 |
| | | $CO_2$ | 1.59 | 3.95 | 2.37 | -0.61 | 0.09 | 0.70 |

Continued on next page

**Table S8 – Continued from previous page**

| Model | Surface | Species | $d_{\mathrm{DFT}}$ / Å | $d_{\mathrm{Pred}}$ / Å | $\lvert\Delta d\rvert$ | $E_{\mathrm{DFT}}$ / eV | $E_{\mathrm{Pred}}$ / eV | $\lvert\Delta E\rvert$ |
|---|---|---|---|---|---|---|---|---|
| | | H | 1.39 | 1.72 | 0.34 | -0.45 | -0.57 | 0.12 |
| | | $H_2O$ | 1.75 | 2.07 | 0.32 | – | – | – |
| | | O | 1.92 | 1.76 | 0.15 | 0.28 | 2.21 | 1.93 |
| | | OH | 2.06 | 1.94 | 0.12 | -0.55 | 0.92 | 1.47 |
| Orb-MPTrj-V2 | Ni(111) | CO | 1.97 | 1.90 | 0.08 | -2.15 | -1.87 | 0.28 |
| | | $CO_2$ | 2.13 | 5.62 | 3.48 | -0.06 | 0.11 | 0.17 |
| | | H | 1.63 | 1.65 | 0.02 | -0.63 | -0.84 | 0.21 |
| | | $H_2O$ | 2.15 | 2.06 | 0.09 | – | – | – |
| | | O | 1.83 | 1.85 | 0.02 | -0.00 | 0.78 | 0.78 |
| | | OH | 1.91 | 1.95 | 0.04 | -0.33 | 0.26 | 0.59 |
| Orb-MPTrj-V2 | Pd(111) | CO | 2.06 | 2.01 | 0.05 | -2.36 | -1.98 | 0.37 |
| | | $CO_2$ | 2.06 | 3.25 | 1.19 | -0.00 | 0.12 | 0.13 |
| | | H | 1.80 | 1.76 | 0.04 | -0.72 | -0.96 | 0.24 |
| | | $H_2O$ | 2.35 | 2.10 | 0.25 | – | – | – |
| | | O | 1.99 | 2.01 | 0.02 | 0.85 | 0.94 | 0.09 |
| | | OH | 2.14 | 1.96 | 0.18 | 0.38 | 0.96 | 0.57 |
| Orb-V2 | Ag(111) | CO | 1.92 | 2.16 | 0.24 | -0.40 | -0.51 | 0.11 |
| | | $CO_2$ | 2.18 | 5.70 | 3.52 | -0.11 | 0.09 | 0.19 |
| | | H | 1.86 | 1.96 | 0.10 | 0.07 | -0.05 | 0.12 |
| | | $H_2O$ | 2.37 | 2.61 | 0.24 | – | – | – |
| | | O | 2.02 | 2.15 | 0.12 | 1.84 | 1.93 | 0.09 |
| | | OH | 2.11 | 2.31 | 0.20 | 0.38 | 0.28 | 0.10 |
| Orb-V2 | Au(111) | CO | 2.16 | 2.00 | 0.16 | -0.49 | -0.68 | 0.18 |
| | | $CO_2$ | 2.18 | 5.25 | 3.07 | -0.10 | 0.07 | 0.17 |
| | | H | 1.87 | 1.91 | 0.04 | 0.04 | 0.00 | 0.04 |
| | | $H_2O$ | 2.37 | 3.05 | 0.67 | – | – | – |
| | | O | 2.03 | 2.14 | 0.11 | 2.32 | 2.47 | 0.15 |
| | | OH | 2.11 | 2.10 | 0.01 | 1.04 | 1.33 | 0.30 |
| Orb-V2 | Ni(110) | CO | 1.97 | 1.99 | 0.02 | -2.01 | 37.96 | 39.97 |
| | | $CO_2$ | 1.59 | 4.43 | 2.85 | -0.61 | 0.12 | 0.73 |
| | | H | 1.39 | 1.53 | 0.14 | -0.45 | 9.36 | 9.82 |
| | | $H_2O$ | 1.75 | 2.16 | 0.41 | – | – | – |
| | | O | 1.92 | 1.80 | 0.12 | 0.28 | 116.94 | 116.67 |
| | | OH | 2.06 | 2.06 | 0.00 | -0.55 | 114.82 | 115.37 |
| Orb-V2 | Ni(111) | CO | 1.97 | 1.77 | 0.20 | -2.15 | 46.48 | 48.63 |
| | | $CO_2$ | 2.13 | 5.27 | 3.13 | -0.06 | -0.02 | 0.03 |
| | | H | 1.63 | 1.48 | 0.15 | -0.63 | 0.19 | 0.82 |
| | | $H_2O$ | 2.15 | 2.23 | 0.08 | – | – | – |
| | | O | 1.83 | 1.71 | 0.12 | -0.00 | 70.00 | 70.00 |
| | | OH | 1.91 | 1.85 | 0.06 | -0.33 | 65.75 | 66.07 |
| Orb-V2 | Pd(111) | CO | 2.06 | 2.06 | 0.00 | -2.36 | -2.01 | 0.35 |
| | | $CO_2$ | 2.06 | 8.61 | 6.55 | -0.00 | 0.01 | 0.02 |
| | | H | 1.80 | 1.81 | 0.01 | -0.72 | -0.52 | 0.20 |
| | | $H_2O$ | 2.35 | 2.41 | 0.06 | – | – | – |
| | | O | 1.99 | 2.02 | 0.03 | 0.85 | 1.35 | 0.49 |
| | | OH | 2.14 | 1.98 | 0.16 | 0.38 | 0.90 | 0.52 |
| Orb-V3-conservative-20-mpa | Ag(111) | CO | 1.92 | 2.15 | 0.23 | -0.40 | -0.33 | 0.07 |

Continued on next page

**Table S8 – Continued from previous page**

| Model | Surface | Species | $d_{DFT}$ / Å | $d_{Pred}$ / Å | $\|\Delta d\|$ | $E_{DFT}$ / eV | $E_{Pred}$ / eV | $\|\Delta E\|$ |
|---|---|---|---|---|---|---|---|---|
| | | $CO_2$ | 2.18 | 5.62 | 3.44 | -0.11 | 0.05 | 0.15 |
| | | H | 1.86 | 1.93 | 0.07 | 0.07 | 0.31 | 0.24 |
| | | $H_2O$ | 2.37 | 2.89 | 0.52 | – | – | – |
| | | O | 2.02 | 2.15 | 0.13 | 1.84 | 2.18 | 0.34 |
| | | OH | 2.11 | 3.13 | 1.02 | 0.38 | 0.94 | 0.57 |
| Orb-V3-conservative-20-mpa | Au(111) | CO | 2.16 | 2.00 | 0.16 | -0.49 | -0.47 | 0.02 |
| | | $CO_2$ | 2.18 | 3.99 | 1.81 | -0.10 | -0.01 | 0.08 |
| | | H | 1.87 | 1.89 | 0.03 | 0.04 | 0.27 | 0.23 |
| | | $H_2O$ | 2.37 | 2.86 | 0.48 | – | – | – |
| | | O | 2.03 | 2.14 | 0.11 | 2.32 | 2.60 | 0.28 |
| | | OH | 2.11 | 2.08 | 0.03 | 1.04 | 1.50 | 0.47 |
| Orb-V3-conservative-20-mpa | Ni(110) | CO | 1.97 | 3.70 | 1.73 | -2.01 | 3.07 | 5.08 |
| | | $CO_2$ | 1.59 | 1.94 | 0.35 | -0.61 | 57.09 | 57.70 |
| | | H | 1.39 | 1.77 | 0.38 | -0.45 | -0.41 | 0.05 |
| | | $H_2O$ | 1.75 | 1.47 | 0.28 | – | – | – |
| | | O | 1.92 | 3.00 | 1.08 | 0.28 | 16.83 | 16.55 |
| | | OH | 2.06 | 3.12 | 1.06 | -0.55 | 19.23 | 19.78 |
| Orb-V3-conservative-20-mpa | Ni(111) | CO | 1.97 | 1.89 | 0.08 | -2.15 | 9.60 | 11.75 |
| | | $CO_2$ | 2.13 | 1.64 | 0.50 | -0.06 | 34.78 | 34.84 |
| | | H | 1.63 | 1.69 | 0.06 | -0.63 | -0.58 | 0.05 |
| | | $H_2O$ | 2.15 | 1.64 | 0.51 | – | – | – |
| | | O | 1.83 | 1.93 | 0.10 | -0.00 | 31.45 | 31.45 |
| | | OH | 1.91 | 2.95 | 1.03 | -0.33 | 16.59 | 16.91 |
| Orb-V3-conservative-20-mpa | Pd(111) | CO | 2.06 | 1.99 | 0.06 | -2.36 | -1.89 | 0.47 |
| | | $CO_2$ | 2.06 | 3.04 | 0.98 | -0.00 | 0.10 | 0.10 |
| | | H | 1.80 | 1.80 | 0.00 | -0.72 | -0.45 | 0.27 |
| | | $H_2O$ | 2.35 | 2.56 | 0.21 | – | – | – |
| | | O | 1.99 | 2.00 | 0.02 | 0.85 | 1.21 | 0.36 |
| | | OH | 2.14 | 1.98 | 0.17 | 0.38 | 0.94 | 0.56 |
| Orb-V3-conservative-20-omat | Ag(111) | CO | 1.92 | 2.16 | 0.24 | -0.40 | -0.17 | 0.23 |
| | | $CO_2$ | 2.18 | 5.68 | 3.50 | -0.11 | 0.11 | 0.22 |
| | | H | 1.86 | 1.93 | 0.06 | 0.07 | 0.34 | 0.27 |
| | | $H_2O$ | 2.37 | 2.84 | 0.46 | – | – | – |
| | | O | 2.02 | 2.14 | 0.12 | 1.84 | 2.31 | 0.47 |
| | | OH | 2.11 | 2.30 | 0.19 | 0.38 | 1.02 | 0.64 |
| Orb-V3-conservative-20-omat | Au(111) | CO | 2.16 | 2.01 | 0.15 | -0.49 | -0.38 | 0.11 |
| | | $CO_2$ | 2.18 | 4.17 | 1.99 | -0.10 | 0.04 | 0.14 |
| | | H | 1.87 | 1.89 | 0.02 | 0.04 | 0.31 | 0.27 |
| | | $H_2O$ | 2.37 | 2.84 | 0.46 | – | – | – |
| | | O | 2.03 | 2.13 | 0.10 | 2.32 | 2.76 | 0.44 |
| | | OH | 2.11 | 2.07 | 0.04 | 1.04 | 1.63 | 0.60 |
| Orb-V3-conservative-20-omat | Ni(110) | CO | 1.97 | 1.94 | 0.03 | -2.01 | 0.39 | 2.40 |
| | | $CO_2$ | 1.59 | 3.25 | 1.66 | -0.61 | 1.13 | 1.74 |
| | | H | 1.39 | 1.60 | 0.22 | -0.45 | -0.44 | 0.02 |
| | | $H_2O$ | 1.75 | 3.51 | 1.76 | – | – | – |
| | | O | 1.92 | 2.76 | 0.84 | 0.28 | 6.91 | 6.63 |
| | | OH | 2.06 | 2.84 | 0.78 | -0.55 | 3.53 | 4.08 |

Continued on next page

**Table S8 – Continued from previous page**

| Model | Surface | Species | $d_{\mathrm{DFT}}$ / Å | $d_{\mathrm{Pred}}$ / Å | $\lvert\Delta d\rvert$ | $E_{\mathrm{DFT}}$ / eV | $E_{\mathrm{Pred}}$ / eV | $\lvert\Delta E\rvert$ |
|---|---|---|---|---|---|---|---|---|
| Orb-V3-conservative-20-omat | Ni(111) | CO | 1.97 | 1.88 | 0.09 | -2.15 | -0.24 | 1.91 |
| | | $CO_2$ | 2.13 | 5.90 | 3.76 | -0.06 | 0.04 | 0.10 |
| | | H | 1.63 | 1.68 | 0.05 | -0.63 | -0.59 | 0.04 |
| | | $H_2O$ | 2.15 | 2.85 | 0.71 | – | – | – |
| | | O | 1.83 | 2.78 | 0.95 | -0.00 | 7.43 | 7.43 |
| | | OH | 1.91 | 2.07 | 0.15 | -0.33 | 5.47 | 5.80 |
| Orb-V3-conservative-20-omat | Pd(111) | CO | 2.06 | 1.98 | 0.08 | -2.36 | -2.07 | 0.29 |
| | | $CO_2$ | 2.06 | 3.31 | 1.25 | -0.00 | 0.02 | 0.02 |
| | | H | 1.80 | 1.80 | 0.00 | -0.72 | -0.72 | 0.00 |
| | | $H_2O$ | 2.35 | 2.47 | 0.12 | – | – | – |
| | | O | 1.99 | 2.00 | 0.01 | 0.85 | 0.99 | 0.14 |
| | | OH | 2.14 | 1.96 | 0.18 | 0.38 | 0.86 | 0.48 |
| Orb-V3-conservative-inf-mpa | Ag(111) | CO | 1.92 | 2.10 | 0.18 | -0.40 | -0.48 | 0.08 |
| | | $CO_2$ | 2.18 | 3.55 | 1.37 | -0.11 | -0.06 | 0.04 |
| | | H | 1.86 | 1.92 | 0.06 | 0.07 | 0.16 | 0.09 |
| | | $H_2O$ | 2.37 | 2.63 | 0.26 | – | – | – |
| | | O | 2.02 | 2.14 | 0.12 | 1.84 | 1.99 | 0.15 |
| | | OH | 2.11 | 2.29 | 0.18 | 0.38 | 0.44 | 0.07 |
| Orb-V3-conservative-inf-mpa | Au(111) | CO | 2.16 | 1.98 | 0.18 | -0.49 | -0.57 | 0.08 |
| | | $CO_2$ | 2.18 | 3.79 | 1.61 | -0.10 | -0.05 | 0.05 |
| | | H | 1.87 | 1.88 | 0.02 | 0.04 | 0.13 | 0.08 |
| | | $H_2O$ | 2.37 | 2.77 | 0.40 | – | – | – |
| | | O | 2.03 | 2.13 | 0.10 | 2.32 | 2.39 | 0.07 |
| | | OH | 2.11 | 2.08 | 0.04 | 1.04 | 1.35 | 0.31 |
| Orb-V3-conservative-inf-mpa | Ni(110) | CO | 1.97 | 4.70 | 2.73 | -2.01 | -0.07 | 1.94 |
| | | $CO_2$ | 1.59 | 5.89 | 4.30 | -0.61 | 0.02 | 0.63 |
| | | H | 1.39 | 1.61 | 0.23 | -0.45 | -0.45 | 0.00 |
| | | $H_2O$ | 1.75 | 7.18 | 5.43 | – | – | – |
| | | O | 1.92 | 5.92 | 4.01 | 0.28 | 5.20 | 4.92 |
| | | OH | 2.06 | 5.84 | 3.78 | -0.55 | 2.95 | 3.50 |
| Orb-V3-conservative-inf-mpa | Ni(111) | CO | 1.97 | 5.74 | 3.76 | -2.15 | -0.02 | 2.13 |
| | | $CO_2$ | 2.13 | 6.78 | 4.65 | -0.06 | 0.03 | 0.08 |
| | | H | 1.63 | 1.71 | 0.08 | -0.63 | -0.60 | 0.03 |
| | | $H_2O$ | 2.15 | 5.16 | 3.01 | – | – | – |
| | | O | 1.83 | 5.98 | 4.15 | -0.00 | 5.19 | 5.19 |
| | | OH | 1.91 | 6.40 | 4.49 | -0.33 | 2.95 | 3.27 |
| Orb-V3-conservative-inf-mpa | Pd(111) | CO | 2.06 | 2.08 | 0.02 | -2.36 | -2.16 | 0.19 |
| | | $CO_2$ | 2.06 | 3.49 | 1.42 | -0.00 | -0.08 | 0.08 |
| | | H | 1.80 | 1.82 | 0.02 | -0.72 | -0.56 | 0.16 |
| | | $H_2O$ | 2.35 | 2.32 | 0.03 | – | – | – |
| | | O | 1.99 | 2.01 | 0.02 | 0.85 | 1.21 | 0.35 |
| | | OH | 2.14 | 1.96 | 0.18 | 0.38 | 0.80 | 0.42 |
| Orb-V3-conservative-inf-omat | Ag(111) | CO | 1.92 | 2.09 | 0.17 | -0.40 | -0.46 | 0.06 |
| | | $CO_2$ | 2.18 | 3.35 | 1.17 | -0.11 | -0.05 | 0.06 |
| | | H | 1.86 | 1.93 | 0.07 | 0.07 | 0.06 | 0.01 |
| | | $H_2O$ | 2.37 | 2.63 | 0.26 | – | – | – |
| | | O | 2.02 | 2.14 | 0.11 | 1.84 | 1.98 | 0.14 |

Continued on next page

Table S8 – Continued from previous page

| Model | Surface | Species | $d_{DFT}$ / Å | $d_{Pred}$ / Å | $\|\Delta d\|$ | $E_{DFT}$ / eV | $E_{Pred}$ / eV | $\|\Delta E\|$ |
|---|---|---|---|---|---|---|---|---|
| | | OH | 2.11 | 2.28 | 0.17 | 0.38 | 0.51 | 0.13 |
| Orb-V3-conservative-inf-omat | Au(111) | CO | 2.16 | 1.98 | 0.19 | -0.49 | -0.35 | 0.14 |
| | | $CO_2$ | 2.18 | 4.08 | 1.90 | -0.10 | 0.10 | 0.20 |
| | | H | 1.87 | 1.89 | 0.02 | 0.04 | 0.21 | 0.17 |
| | | $H_2O$ | 2.37 | 2.93 | 0.55 | – | – | – |
| | | O | 2.03 | 2.12 | 0.09 | 2.32 | 2.75 | 0.44 |
| | | OH | 2.11 | 2.06 | 0.05 | 1.04 | 1.55 | 0.51 |
| Orb-V3-conservative-inf-omat | Ni(110) | CO | 1.97 | 1.87 | 0.10 | -2.01 | 3.30 | 5.31 |
| | | $CO_2$ | 1.59 | 1.84 | 0.26 | -0.61 | 14.67 | 15.27 |
| | | H | 1.39 | 1.61 | 0.22 | -0.45 | -0.43 | 0.03 |
| | | $H_2O$ | 1.75 | 1.85 | 0.10 | – | – | – |
| | | O | 1.92 | 3.18 | 1.26 | 0.28 | 6.32 | 6.04 |
| | | OH | 2.06 | 2.39 | 0.33 | -0.55 | 4.27 | 4.82 |
| Orb-V3-conservative-inf-omat | Pd(111) | CO | 2.06 | 1.85 | 0.21 | -2.36 | -1.48 | 0.87 |
| | | $CO_2$ | 2.06 | 3.74 | 1.68 | -0.00 | 0.05 | 0.06 |
| | | H | 1.80 | 1.82 | 0.01 | -0.72 | -0.52 | 0.20 |
| | | $H_2O$ | 2.35 | 2.31 | 0.03 | – | – | – |
| | | O | 1.99 | 2.00 | 0.01 | 0.85 | 1.35 | 0.49 |
| | | OH | 2.14 | 1.96 | 0.18 | 0.38 | 0.94 | 0.56 |
| Orb-V3-direct-20-mpa | Ag(111) | CO | 1.92 | 2.13 | 0.21 | -0.40 | -0.36 | 0.04 |
| | | $CO_2$ | 2.18 | 3.84 | 1.66 | -0.11 | 0.07 | 0.17 |
| | | H | 1.86 | 1.92 | 0.05 | 0.07 | 0.19 | 0.12 |
| | | $H_2O$ | 2.37 | 2.63 | 0.26 | – | – | – |
| | | O | 2.02 | 2.14 | 0.12 | 1.84 | 2.44 | 0.60 |
| | | OH | 2.11 | 2.29 | 0.18 | 0.38 | 0.73 | 0.35 |
| Orb-V3-direct-20-mpa | Au(111) | CO | 2.16 | 2.00 | 0.16 | -0.49 | -0.49 | 0.01 |
| | | $CO_2$ | 2.18 | 3.73 | 1.55 | -0.10 | -0.02 | 0.07 |
| | | H | 1.87 | 1.88 | 0.02 | 0.04 | 0.11 | 0.06 |
| | | $H_2O$ | 2.37 | 2.86 | 0.49 | – | – | – |
| | | O | 2.03 | 2.13 | 0.11 | 2.32 | 2.81 | 0.49 |
| | | OH | 2.11 | 2.26 | 0.15 | 1.04 | 1.36 | 0.33 |
| Orb-V3-direct-20-mpa | Ni(110) | CO | 1.97 | 1.88 | 0.09 | -2.01 | 6.59 | 8.60 |
| | | $CO_2$ | 1.59 | 1.96 | 0.38 | -0.61 | 21.65 | 22.25 |
| | | H | 1.39 | 1.62 | 0.24 | -0.45 | -0.54 | 0.09 |
| | | $H_2O$ | 1.75 | 3.01 | 1.26 | – | – | – |
| | | O | 1.92 | 1.68 | 0.24 | 0.28 | 12.62 | 12.34 |
| | | OH | 2.06 | 1.99 | 0.07 | -0.55 | 14.12 | 14.67 |
| Orb-V3-direct-20-mpa | Ni(111) | CO | 1.97 | 1.92 | 0.06 | -2.15 | 6.84 | 8.99 |
| | | $CO_2$ | 2.13 | 3.89 | 1.76 | -0.06 | 4.03 | 4.09 |
| | | H | 1.63 | 1.70 | 0.07 | -0.63 | -0.72 | 0.09 |
| | | $H_2O$ | 2.15 | 2.15 | 0.00 | – | – | – |
| | | O | 1.83 | 1.88 | 0.05 | -0.00 | 22.95 | 22.95 |
| | | OH | 1.91 | 2.00 | 0.09 | -0.33 | 19.07 | 19.39 |
| Orb-V3-direct-20-mpa | Pd(111) | CO | 2.06 | 1.99 | 0.07 | -2.36 | -1.69 | 0.66 |
| | | $CO_2$ | 2.06 | 3.74 | 1.68 | -0.00 | 0.06 | 0.06 |
| | | H | 1.80 | 1.80 | 0.00 | -0.72 | -0.60 | 0.12 |
| | | $H_2O$ | 2.35 | 2.35 | 0.00 | – | – | – |

Continued on next page

**Table S8 – Continued from previous page**

| Model | Surface | Species | $d_{DFT}$ / Å | $d_{Pred}$ / Å | $\|\Delta d\|$ | $E_{DFT}$ / eV | $E_{Pred}$ / eV | $\|\Delta E\|$ |
|---|---|---|---|---|---|---|---|---|
| | | O | 1.99 | 1.99 | 0.00 | 0.85 | 1.64 | 0.79 |
| | | OH | 2.14 | 1.98 | 0.16 | 0.38 | 1.14 | 0.76 |
| Orb-V3-direct-20-omat | Ag(111) | CO | 1.92 | 2.10 | 0.18 | -0.40 | -0.34 | 0.06 |
| | | $CO_2$ | 2.18 | 4.05 | 1.87 | -0.11 | 0.05 | 0.16 |
| | | H | 1.86 | 1.91 | 0.05 | 0.07 | -0.01 | 0.08 |
| | | $H_2O$ | 2.37 | 2.61 | 0.24 | – | – | – |
| | | O | 2.02 | 2.14 | 0.11 | 1.84 | 2.17 | 0.33 |
| | | OH | 2.11 | 2.27 | 0.16 | 0.38 | 0.55 | 0.18 |
| Orb-V3-direct-20-omat | Au(111) | CO | 2.16 | 1.97 | 0.19 | -0.49 | -0.33 | 0.16 |
| | | $CO_2$ | 2.18 | 3.79 | 1.61 | -0.10 | -0.03 | 0.06 |
| | | H | 1.87 | 1.89 | 0.02 | 0.04 | 0.20 | 0.16 |
| | | $H_2O$ | 2.37 | 2.93 | 0.56 | – | – | – |
| | | O | 2.03 | 2.13 | 0.10 | 2.32 | 2.87 | 0.55 |
| | | OH | 2.11 | 2.08 | 0.03 | 1.04 | 1.67 | 0.63 |
| Orb-V3-direct-20-omat | Ni(110) | CO | 1.97 | 1.70 | 0.27 | -2.01 | 6.26 | 8.27 |
| | | $CO_2$ | 1.59 | 1.96 | 0.37 | -0.61 | 35.39 | 36.00 |
| | | H | 1.39 | 1.61 | 0.22 | -0.45 | -0.45 | 0.00 |
| | | $H_2O$ | 1.75 | 2.08 | 0.33 | – | – | – |
| | | O | 1.92 | 1.79 | 0.13 | 0.28 | 26.73 | 26.45 |
| | | OH | 2.06 | 2.00 | 0.06 | -0.55 | 19.50 | 20.05 |
| Orb-V3-direct-20-omat | Ni(111) | CO | 1.97 | 1.71 | 0.27 | -2.15 | 6.39 | 8.54 |
| | | $CO_2$ | 2.13 | 1.89 | 0.24 | -0.06 | 31.04 | 31.09 |
| | | H | 1.63 | 1.70 | 0.07 | -0.63 | -0.69 | 0.06 |
| | | $H_2O$ | 2.15 | 2.12 | 0.02 | – | – | – |
| | | O | 1.83 | 1.88 | 0.05 | -0.00 | 36.70 | 36.70 |
| | | OH | 1.91 | 1.82 | 0.09 | -0.33 | 21.60 | 21.92 |
| Orb-V3-direct-20-omat | Pd(111) | CO | 2.06 | 1.97 | 0.08 | -2.36 | -1.41 | 0.95 |
| | | $CO_2$ | 2.06 | 7.63 | 5.57 | -0.00 | 0.09 | 0.09 |
| | | H | 1.80 | 1.81 | 0.00 | -0.72 | -0.34 | 0.38 |
| | | $H_2O$ | 2.35 | 2.60 | 0.25 | – | – | – |
| | | O | 1.99 | 1.99 | 0.00 | 0.85 | 1.94 | 1.09 |
| | | OH | 2.14 | 2.12 | 0.02 | 0.38 | 1.00 | 0.62 |
| Orb-V3-direct-inf-mpa | Ag(111) | CO | 1.92 | 2.12 | 0.20 | -0.40 | -0.46 | 0.06 |
| | | $CO_2$ | 2.18 | 3.97 | 1.79 | -0.11 | 0.13 | 0.23 |
| | | H | 1.86 | 1.91 | 0.05 | 0.07 | 0.21 | 0.14 |
| | | $H_2O$ | 2.37 | 2.61 | 0.24 | – | – | – |
| | | O | 2.02 | 2.15 | 0.13 | 1.84 | 2.41 | 0.57 |
| | | OH | 2.11 | 2.29 | 0.18 | 0.38 | 0.83 | 0.45 |
| Orb-V3-direct-inf-mpa | Au(111) | CO | 2.16 | 2.00 | 0.16 | -0.49 | -0.65 | 0.16 |
| | | $CO_2$ | 2.18 | 3.90 | 1.71 | -0.10 | 0.07 | 0.17 |
| | | H | 1.87 | 1.88 | 0.01 | 0.04 | 0.08 | 0.03 |
| | | $H_2O$ | 2.37 | 2.91 | 0.53 | – | – | – |
| | | O | 2.03 | 2.14 | 0.12 | 2.32 | 2.71 | 0.40 |
| | | OH | 2.11 | 2.09 | 0.02 | 1.04 | 1.51 | 0.48 |
| Orb-V3-direct-inf-mpa | Ni(110) | CO | 1.97 | 1.93 | 0.04 | -2.01 | 0.83 | 2.84 |
| | | $CO_2$ | 1.59 | 2.16 | 0.58 | -0.61 | 10.47 | 11.08 |
| | | H | 1.39 | 1.76 | 0.38 | -0.45 | -0.52 | 0.07 |

Continued on next page

Table S8 – Continued from previous page

| Model | Surface | Species | $d_{DFT}$ / Å | $d_{Pred}$ / Å | $\|\Delta d\|$ | $E_{DFT}$ / eV | $E_{Pred}$ / eV | $\|\Delta E\|$ |
|---|---|---|---|---|---|---|---|---|
| | | $H_2O$ | 1.75 | 3.20 | 1.45 | – | – | – |
| | | O | 1.92 | 1.68 | 0.24 | 0.28 | 7.03 | 6.75 |
| | | OH | 2.06 | 2.02 | 0.04 | -0.55 | 6.27 | 6.82 |
| Orb-V3-direct-inf-mpa | Ni(111) | CO | 1.97 | 1.75 | 0.23 | -2.15 | -0.90 | 1.25 |
| | | $CO_2$ | 2.13 | 3.80 | 1.67 | -0.06 | 1.22 | 1.28 |
| | | H | 1.63 | 1.71 | 0.08 | -0.63 | -0.71 | 0.08 |
| | | $H_2O$ | 2.15 | 2.18 | 0.03 | – | – | – |
| | | O | 1.83 | 1.90 | 0.07 | -0.00 | 9.15 | 9.15 |
| | | OH | 1.91 | 1.85 | 0.06 | -0.33 | 4.53 | 4.85 |
| Orb-V3-direct-inf-mpa | Pd(111) | CO | 2.06 | 2.07 | 0.02 | -2.36 | -2.13 | 0.23 |
| | | $CO_2$ | 2.06 | 3.63 | 1.57 | -0.00 | 0.01 | 0.01 |
| | | H | 1.80 | 1.82 | 0.02 | -0.72 | -0.56 | 0.16 |
| | | $H_2O$ | 2.35 | 2.38 | 0.03 | – | – | – |
| | | O | 1.99 | 1.99 | 0.01 | 0.85 | 1.42 | 0.57 |
| | | OH | 2.14 | 1.98 | 0.16 | 0.38 | 1.04 | 0.66 |
| Orb-V3-direct-inf-omat | Ag(111) | CO | 1.92 | 2.14 | 0.22 | -0.40 | -0.23 | 0.17 |
| | | $CO_2$ | 2.18 | 3.92 | 1.74 | -0.11 | 0.20 | 0.31 |
| | | H | 1.86 | 1.91 | 0.05 | 0.07 | 0.12 | 0.05 |
| | | $H_2O$ | 2.37 | 2.60 | 0.22 | – | – | – |
| | | O | 2.02 | 2.15 | 0.13 | 1.84 | 2.37 | 0.53 |
| | | OH | 2.11 | 2.29 | 0.18 | 0.38 | 0.85 | 0.47 |
| Orb-V3-direct-inf-omat | Au(111) | CO | 2.16 | 1.97 | 0.19 | -0.49 | -0.41 | 0.08 |
| | | $CO_2$ | 2.18 | 4.14 | 1.96 | -0.10 | 0.17 | 0.27 |
| | | H | 1.87 | 1.87 | 0.01 | 0.04 | -0.12 | 0.16 |
| | | $H_2O$ | 2.37 | 2.96 | 0.59 | – | – | – |
| | | O | 2.03 | 2.12 | 0.10 | 2.32 | 2.62 | 0.30 |
| | | OH | 2.11 | 2.09 | 0.02 | 1.04 | 1.48 | 0.44 |
| Orb-V3-direct-inf-omat | Ni(110) | CO | 1.97 | 1.88 | 0.09 | -2.01 | -0.37 | 1.64 |
| | | $CO_2$ | 1.59 | 6.12 | 4.53 | -0.61 | 0.06 | 0.67 |
| | | H | 1.39 | 1.63 | 0.24 | -0.45 | -0.43 | 0.03 |
| | | $H_2O$ | 1.75 | 4.87 | 3.12 | – | – | – |
| | | O | 1.92 | 1.70 | 0.22 | 0.28 | 6.24 | 5.97 |
| | | OH | 2.06 | 2.01 | 0.05 | -0.55 | 5.09 | 5.64 |
| Orb-V3-direct-inf-omat | Ni(111) | CO | 1.97 | 1.95 | 0.02 | -2.15 | -0.75 | 1.40 |
| | | $CO_2$ | 2.13 | 4.44 | 2.31 | -0.06 | 0.36 | 0.42 |
| | | H | 1.63 | 1.71 | 0.08 | -0.63 | -0.69 | 0.07 |
| | | $H_2O$ | 2.15 | 2.21 | 0.06 | – | – | – |
| | | O | 1.83 | 1.90 | 0.07 | -0.00 | 6.41 | 6.41 |
| | | OH | 1.91 | 1.85 | 0.06 | -0.33 | 3.19 | 3.52 |
| Orb-V3-direct-inf-omat | Pd(111) | CO | 2.06 | 2.06 | 0.01 | -2.36 | -2.07 | 0.29 |
| | | $CO_2$ | 2.06 | 3.81 | 1.75 | -0.00 | -0.05 | 0.04 |
| | | H | 1.80 | 1.81 | 0.00 | -0.72 | -0.70 | 0.02 |
| | | $H_2O$ | 2.35 | 2.40 | 0.06 | – | – | – |
| | | O | 1.99 | 1.97 | 0.01 | 0.85 | 1.32 | 0.46 |
| | | OH | 2.14 | 1.96 | 0.18 | 0.38 | 0.96 | 0.58 |

**Table S9:** Performance metrics for PET-MAD models

| Model | Surface | Species | $d_{\text{DFT}}$ / Å | $d_{\text{Pred}}$ / Å | $\|\Delta d\|$ | $E_{\text{DFT}}$ / eV | $E_{\text{Pred}}$ / eV | $\|\Delta E\|$ |
|---|---|---|---|---|---|---|---|---|
| PET-MAD-latest | Ag(111) | CO | 1.92 | 2.07 | 0.15 | -0.40 | -0.53 | 0.13 |
| | | $CO_2$ | 2.18 | 4.07 | 1.89 | -0.11 | -0.12 | 0.02 |
| | | H | 1.86 | 1.92 | 0.06 | 0.07 | -0.37 | 0.44 |
| | | $H_2O$ | 2.37 | 2.76 | 0.38 | – | – | – |
| | | O | 2.02 | 2.10 | 0.07 | 1.84 | 2.77 | 0.93 |
| | | OH | 2.11 | 2.24 | 0.13 | 0.38 | 0.92 | 0.54 |
| PET-MAD-latest | Au(111) | CO | 2.16 | 2.09 | 0.08 | -0.49 | -1.30 | 0.81 |
| | | $CO_2$ | 2.18 | 3.76 | 1.58 | -0.10 | -0.08 | 0.01 |
| | | H | 1.87 | 1.90 | 0.04 | 0.04 | -0.47 | 0.51 |
| | | $H_2O$ | 2.37 | 2.54 | 0.16 | – | – | – |
| | | O | 2.03 | 2.10 | 0.07 | 2.32 | 2.95 | 0.63 |
| | | OH | 2.11 | 2.20 | 0.09 | 1.04 | 1.31 | 0.28 |
| PET-MAD-latest | Ni(110) | CO | 1.97 | 1.85 | 0.12 | -2.01 | -2.38 | 0.37 |
| | | $CO_2$ | 1.59 | 1.89 | 0.30 | -0.61 | -1.51 | 0.90 |
| | | H | 1.39 | 1.75 | 0.36 | -0.45 | -1.05 | 0.60 |
| | | $H_2O$ | 1.75 | 2.31 | 0.56 | – | – | – |
| | | O | 1.92 | 1.72 | 0.20 | 0.28 | 1.12 | 0.84 |
| | | OH | 2.06 | 1.95 | 0.11 | -0.55 | 0.29 | 0.84 |
| PET-MAD-latest | Ni(111) | CO | 1.97 | 1.86 | 0.11 | -2.15 | -2.13 | 0.02 |
| | | $CO_2$ | 2.13 | 2.01 | 0.13 | -0.06 | -0.39 | 0.33 |
| | | H | 1.63 | 1.69 | 0.05 | -0.63 | -1.17 | 0.55 |
| | | $H_2O$ | 2.15 | 2.06 | 0.08 | – | – | – |
| | | O | 1.83 | 1.81 | 0.02 | -0.00 | 0.89 | 0.89 |
| | | OH | 1.91 | 1.93 | 0.02 | -0.33 | -0.02 | 0.30 |
| PET-MAD-latest | Pd(111) | CO | 2.06 | 1.95 | 0.11 | -2.36 | -2.51 | 0.15 |
| | | $CO_2$ | 2.06 | 2.51 | 0.45 | -0.00 | -0.24 | 0.24 |
| | | H | 1.80 | 1.80 | 0.00 | -0.72 | -1.16 | 0.44 |
| | | $H_2O$ | 2.35 | 2.45 | 0.10 | – | – | – |
| | | O | 1.99 | 1.95 | 0.03 | 0.85 | 1.92 | 1.06 |
| | | OH | 2.14 | 2.11 | 0.04 | 0.38 | 0.90 | 0.52 |

**Table S10:** Performance metrics for SevenNet models

| Model | Surface | Species | $d_{\text{DFT}}$ / Å | $d_{\text{Pred}}$ / Å | $\|\Delta d\|$ | $E_{\text{DFT}}$ / eV | $E_{\text{Pred}}$ / eV | $\|\Delta E\|$ |
|---|---|---|---|---|---|---|---|---|
| SevenN-0 | Ag(111) | CO | 1.92 | 2.17 | 0.25 | -0.40 | -0.34 | 0.06 |
| | | $CO_2$ | 2.18 | 3.80 | 1.62 | -0.11 | -0.32 | 0.22 |
| | | H | 1.86 | 1.92 | 0.06 | 0.07 | 0.04 | 0.03 |
| | | $H_2O$ | 2.37 | 2.95 | 0.57 | – | – | – |
| | | O | 2.02 | 2.16 | 0.14 | 1.84 | 2.23 | 0.39 |
| | | OH | 2.11 | 2.12 | 0.01 | 0.38 | 1.01 | 0.63 |
| SevenN-0 | Au(111) | CO | 2.16 | 2.03 | 0.13 | -0.49 | -0.47 | 0.03 |
| | | $CO_2$ | 2.18 | 3.85 | 1.67 | -0.10 | -0.37 | 0.28 |
| | | H | 1.87 | 1.89 | 0.03 | 0.04 | -0.01 | 0.06 |
| | | $H_2O$ | 2.37 | 3.05 | 0.67 | – | – | – |
| | | O | 2.03 | 2.14 | 0.11 | 2.32 | 2.60 | 0.28 |
| | | OH | 2.11 | 2.09 | 0.03 | 1.04 | 1.23 | 0.20 |

Continued on next page

Table S10 – Continued from previous page

| Model | Surface | Species | $d_{DFT}$ / Å | $d_{Pred}$ / Å | $\|\Delta d\|$ | $E_{DFT}$ / eV | $E_{Pred}$ / eV | $\|\Delta E\|$ |
|---|---|---|---|---|---|---|---|---|
| SevenN-0 | Ni(110) | CO | 1.97 | 5.72 | 3.75 | -2.01 | -0.01 | 2.00 |
| | | $CO_2$ | 1.59 | 6.08 | 4.50 | -0.61 | -0.00 | 0.61 |
| | | H | 1.39 | 1.61 | 0.23 | -0.45 | -0.63 | 0.17 |
| | | $H_2O$ | 1.75 | 5.29 | 3.54 | – | – | – |
| | | O | 1.92 | 6.00 | 4.08 | 0.28 | 5.42 | 5.14 |
| | | OH | 2.06 | 5.61 | 3.55 | -0.55 | 3.07 | 3.62 |
| SevenN-0 | Ni(111) | CO | 1.97 | 5.27 | 3.29 | -2.15 | 0.01 | 2.16 |
| | | $CO_2$ | 2.13 | 6.19 | 4.05 | -0.06 | 0.00 | 0.06 |
| | | H | 1.63 | 1.69 | 0.06 | -0.63 | -0.69 | 0.06 |
| | | $H_2O$ | 2.15 | 5.06 | 2.91 | – | – | – |
| | | O | 1.83 | 6.30 | 4.47 | -0.00 | 5.43 | 5.43 |
| | | OH | 1.91 | 5.22 | 3.31 | -0.33 | 3.08 | 3.41 |
| SevenN-0 | Pd(111) | CO | 2.06 | 2.08 | 0.02 | -2.36 | -2.35 | 0.00 |
| | | $CO_2$ | 2.06 | 3.60 | 1.54 | -0.00 | -0.38 | 0.37 |
| | | H | 1.80 | 1.82 | 0.01 | -0.72 | -0.70 | 0.02 |
| | | $H_2O$ | 2.35 | 2.32 | 0.03 | – | – | – |
| | | O | 1.99 | 2.00 | 0.01 | 0.85 | 1.54 | 0.68 |
| | | OH | 2.14 | 1.97 | 0.17 | 0.38 | 0.82 | 0.44 |
| SevenN-l3i5 | Ag(111) | CO | 1.92 | 2.18 | 0.26 | -0.40 | -0.34 | 0.06 |
| | | $CO_2$ | 2.18 | 3.82 | 1.64 | -0.11 | -0.33 | 0.22 |
| | | H | 1.86 | 1.92 | 0.06 | 0.07 | 0.04 | 0.03 |
| | | $H_2O$ | 2.37 | 2.94 | 0.56 | – | – | – |
| | | O | 2.02 | 2.16 | 0.14 | 1.84 | 2.23 | 0.39 |
| | | OH | 2.11 | 2.12 | 0.01 | 0.38 | 1.01 | 0.63 |
| SevenN-l3i5 | Au(111) | CO | 2.16 | 2.03 | 0.13 | -0.49 | -0.46 | 0.03 |
| | | $CO_2$ | 2.18 | 3.67 | 1.49 | -0.10 | -0.37 | 0.27 |
| | | H | 1.87 | 1.89 | 0.03 | 0.04 | -0.01 | 0.06 |
| | | $H_2O$ | 2.37 | 3.01 | 0.64 | – | – | – |
| | | O | 2.03 | 2.14 | 0.11 | 2.32 | 2.60 | 0.28 |
| | | OH | 2.11 | 2.09 | 0.03 | 1.04 | 1.24 | 0.20 |
| SevenN-l3i5 | Ni(110) | CO | 1.97 | 5.71 | 3.74 | -2.01 | -0.01 | 2.00 |
| | | $CO_2$ | 1.59 | 7.15 | 5.57 | -0.61 | -0.00 | 0.61 |
| | | H | 1.39 | 1.61 | 0.23 | -0.45 | -0.63 | 0.17 |
| | | $H_2O$ | 1.75 | 5.25 | 3.50 | – | – | – |
| | | O | 1.92 | 6.00 | 4.08 | 0.28 | 5.42 | 5.14 |
| | | OH | 2.06 | 5.27 | 3.21 | -0.55 | 3.08 | 3.63 |
| SevenN-l3i5 | Ni(111) | CO | 1.97 | 5.27 | 3.29 | -2.15 | 0.01 | 2.16 |
| | | $CO_2$ | 2.13 | 6.32 | 4.19 | -0.06 | 0.00 | 0.06 |
| | | H | 1.63 | 1.70 | 0.07 | -0.63 | -0.69 | 0.06 |
| | | $H_2O$ | 2.15 | 5.40 | 3.25 | – | – | – |
| | | O | 1.83 | 6.30 | 4.47 | -0.00 | 5.43 | 5.43 |
| | | OH | 1.91 | 5.13 | 3.22 | -0.33 | 3.08 | 3.40 |
| SevenN-l3i5 | Pd(111) | CO | 2.06 | 2.08 | 0.02 | -2.36 | -2.35 | 0.00 |
| | | $CO_2$ | 2.06 | 3.62 | 1.56 | -0.00 | -0.38 | 0.38 |
| | | H | 1.80 | 1.81 | 0.01 | -0.72 | -0.70 | 0.02 |
| | | $H_2O$ | 2.35 | 2.32 | 0.03 | – | – | – |
| | | O | 1.99 | 2.00 | 0.01 | 0.85 | 1.53 | 0.68 |

Continued on next page

Table S10 – Continued from previous page

| Model | Surface | Species | $d_{DFT}$ / Å | $d_{Pred}$ / Å | $\|\Delta d\|$ | $E_{DFT}$ / eV | $E_{Pred}$ / eV | $\|\Delta E\|$ |
|---|---|---|---|---|---|---|---|---|
| | | OH | 2.14 | 1.97 | 0.17 | 0.38 | 0.82 | 0.44 |
| SevenN-mf-ompa-mpa | Ag(111) | CO | 1.92 | 2.19 | 0.27 | -0.40 | -0.34 | 0.06 |
| | | $CO_2$ | 2.18 | 3.80 | 1.62 | -0.11 | -0.33 | 0.22 |
| | | H | 1.86 | 1.92 | 0.06 | 0.07 | 0.04 | 0.03 |
| | | $H_2O$ | 2.37 | 2.91 | 0.53 | – | – | – |
| | | O | 2.02 | 2.16 | 0.14 | 1.84 | 2.23 | 0.39 |
| | | OH | 2.11 | 2.12 | 0.01 | 0.38 | 1.01 | 0.63 |
| SevenN-mf-ompa-mpa | Au(111) | CO | 2.16 | 2.03 | 0.13 | -0.49 | -0.47 | 0.03 |
| | | $CO_2$ | 2.18 | 3.75 | 1.57 | -0.10 | -0.37 | 0.27 |
| | | H | 1.87 | 1.89 | 0.03 | 0.04 | -0.02 | 0.06 |
| | | $H_2O$ | 2.37 | 3.02 | 0.64 | – | – | – |
| | | O | 2.03 | 2.13 | 0.11 | 2.32 | 2.60 | 0.28 |
| | | OH | 2.11 | 2.09 | 0.03 | 1.04 | 1.24 | 0.20 |
| SevenN-mf-ompa-mpa | Ni(110) | CO | 1.97 | 5.69 | 3.72 | -2.01 | -0.01 | 2.00 |
| | | $CO_2$ | 1.59 | 7.30 | 5.71 | -0.61 | -0.00 | 0.61 |
| | | H | 1.39 | 1.61 | 0.23 | -0.45 | -0.63 | 0.18 |
| | | $H_2O$ | 1.75 | 5.26 | 3.51 | – | – | – |
| | | O | 1.92 | 6.00 | 4.08 | 0.28 | 5.42 | 5.14 |
| | | OH | 2.06 | 5.14 | 3.08 | -0.55 | 3.07 | 3.62 |
| SevenN-mf-ompa-mpa | Ni(111) | CO | 1.97 | 5.56 | 3.59 | -2.15 | -0.01 | 2.14 |
| | | $CO_2$ | 2.13 | 6.28 | 4.15 | -0.06 | 0.00 | 0.06 |
| | | H | 1.63 | 1.70 | 0.07 | -0.63 | -0.69 | 0.06 |
| | | $H_2O$ | 2.15 | 5.23 | 3.08 | – | – | – |
| | | O | 1.83 | 6.30 | 4.47 | -0.00 | 5.43 | 5.43 |
| | | OH | 1.91 | 5.17 | 3.26 | -0.33 | 3.08 | 3.41 |
| SevenN-mf-ompa-mpa | Pd(111) | CO | 2.06 | 2.08 | 0.02 | -2.36 | -2.36 | 0.00 |
| | | $CO_2$ | 2.06 | 3.61 | 1.55 | -0.00 | -0.38 | 0.38 |
| | | H | 1.80 | 1.81 | 0.01 | -0.72 | -0.70 | 0.02 |
| | | $H_2O$ | 2.35 | 2.32 | 0.03 | – | – | – |
| | | O | 1.99 | 2.00 | 0.01 | 0.85 | 1.53 | 0.68 |
| | | OH | 2.14 | 1.97 | 0.17 | 0.38 | 0.82 | 0.43 |
| SevenN-omat24 | Ag(111) | CO | 1.92 | 2.18 | 0.26 | -0.40 | -0.34 | 0.06 |
| | | $CO_2$ | 2.18 | 3.80 | 1.62 | -0.11 | -0.32 | 0.22 |
| | | H | 1.86 | 1.92 | 0.06 | 0.07 | 0.04 | 0.03 |
| | | $H_2O$ | 2.37 | 2.91 | 0.53 | – | – | – |
| | | O | 2.02 | 2.16 | 0.14 | 1.84 | 2.23 | 0.39 |
| | | OH | 2.11 | 2.12 | 0.01 | 0.38 | 1.01 | 0.63 |
| SevenN-omat24 | Au(111) | CO | 2.16 | 2.03 | 0.13 | -0.49 | -0.47 | 0.03 |
| | | $CO_2$ | 2.18 | 3.74 | 1.56 | -0.10 | -0.37 | 0.27 |
| | | H | 1.87 | 1.89 | 0.03 | 0.04 | -0.02 | 0.06 |
| | | $H_2O$ | 2.37 | 3.01 | 0.63 | – | – | – |
| | | O | 2.03 | 2.14 | 0.11 | 2.32 | 2.60 | 0.28 |
| | | OH | 2.11 | 2.09 | 0.03 | 1.04 | 1.24 | 0.20 |
| SevenN-omat24 | Ni(110) | CO | 1.97 | 5.38 | 3.41 | -2.01 | 0.00 | 2.01 |
| | | $CO_2$ | 1.59 | 6.10 | 4.52 | -0.61 | -0.00 | 0.61 |
| | | H | 1.39 | 1.61 | 0.23 | -0.45 | -0.62 | 0.17 |
| | | $H_2O$ | 1.75 | 5.42 | 3.67 | – | – | – |

Continued on next page

**Table S10 – Continued from previous page**

| Model | Surface | Species | $d_{DFT}$ / Å | $d_{Pred}$ / Å | $\|\Delta d\|$ | $E_{DFT}$ / eV | $E_{Pred}$ / eV | $\|\Delta E\|$ |
|---|---|---|---|---|---|---|---|---|
| | | O | 1.92 | 6.00 | 4.08 | 0.28 | 5.42 | 5.15 |
| | | OH | 2.06 | 5.25 | 3.19 | -0.55 | 3.08 | 3.63 |
| SevenN-omat24 | Ni(111) | CO | 1.97 | 5.42 | 3.45 | -2.15 | 0.00 | 2.15 |
| | | $CO_2$ | 2.13 | 6.31 | 4.17 | -0.06 | 0.01 | 0.07 |
| | | H | 1.63 | 1.70 | 0.07 | -0.63 | -0.68 | 0.06 |
| | | $H_2O$ | 2.15 | 5.27 | 3.12 | – | – | – |
| | | O | 1.83 | 6.30 | 4.47 | -0.00 | 5.43 | 5.44 |
| | | OH | 1.91 | 5.13 | 3.22 | -0.33 | 3.08 | 3.41 |
| SevenN-omat24 | Pd(111) | CO | 2.06 | 2.08 | 0.02 | -2.36 | -2.35 | 0.00 |
| | | $CO_2$ | 2.06 | 3.50 | 1.44 | -0.00 | -0.38 | 0.37 |
| | | H | 1.80 | 1.81 | 0.00 | -0.72 | -0.70 | 0.02 |
| | | $H_2O$ | 2.35 | 2.32 | 0.03 | – | – | – |
| | | O | 1.99 | 2.00 | 0.01 | 0.85 | 1.54 | 0.68 |
| | | OH | 2.14 | 1.97 | 0.17 | 0.38 | 0.82 | 0.44 |

**Table S11:** Reaction energies [eV] for the reverse water gas shift steps. $R_1$: $H_2(g) \longrightarrow 2\,H^*$, $R_2$: $CO_2(g) \longrightarrow CO_2{}^*$, $R_3$: $CO_2{}^* \longrightarrow CO^* + O^*$, $R_4$: $O^* + H^* \longrightarrow OH^*$, $R_5$: $OH^* + H^* \longrightarrow H_2O^*$, $R_6$: $CO^* \longrightarrow CO\,(g)$, $R_7$: $H_2O^* \longrightarrow H_2O\,(g)$.

| Surface | Model | $R_1$ | $R_2$ | $R_3$ | $R_4$ | $R_5$ | $R_6$ | $R_7$ |
|---|---|---|---|---|---|---|---|---|
| **Ag(111)** | **DFT** | **0.14** | **-0.11** | **2.27** | **-1.53** | **-0.70** | **0.40** | **0.25** |
| | Equiformerv2-153M | 0.66 | -0.48 | 2.66 | -1.38 | -1.56 | 0.06 | 0.03 |
| | Equiformerv2-31M | 0.57 | -0.51 | 2.65 | -1.30 | -1.52 | 0.06 | 0.06 |
| | Equiformerv2-83M | 0.67 | 0.04 | 1.96 | -1.33 | -1.54 | 0.18 | 0.02 |
| | MACE-MATPES-R2SCAN | 0.23 | -0.16 | 2.03 | -0.99 | -1.65 | 0.95 | 1.45 |
| | MACE-MP-0-large | -0.10 | -0.24 | 2.79 | -1.62 | -0.90 | 0.65 | 0.35 |
| | MACE-MP-0-large-b2 | 0.07 | -0.28 | 2.66 | -1.69 | -0.95 | 0.82 | 0.31 |
| | MACE-MP-0-medium | -0.17 | -0.31 | 2.48 | -1.07 | -1.69 | 1.04 | 0.54 |
| | MACE-MP-0-medium-b | 0.10 | -0.33 | 3.00 | -1.15 | -1.62 | 0.81 | 0.57 |
| | MACE-MP-0-medium-b2 | -0.56 | -0.39 | 2.76 | -1.42 | -0.74 | 0.73 | 0.59 |
| | MACE-MP-0-medium-b3 | -0.14 | -0.22 | 2.69 | -0.99 | -1.32 | 0.83 | 0.54 |
| | MACE-MP-0-small | -0.07 | -0.14 | 2.28 | -0.86 | -1.46 | 0.81 | 0.41 |
| | MACE-MP-0-small-b | -2.04 | -0.48 | 3.00 | -1.76 | -0.85 | 1.16 | 0.28 |
| | MACE-MP-0-small-b2 | -0.56 | -0.28 | 2.60 | -0.84 | -1.30 | 0.83 | 0.51 |
| | MACE-MPA-0-medium | -0.05 | -0.33 | 2.62 | -1.07 | -1.43 | 0.71 | 0.55 |
| | MACE-OMat-medium | -0.16 | -0.49 | 2.50 | -1.05 | -1.41 | 1.19 | 0.68 |
| | Orb-D3-SM-V2 | 0.32 | -0.31 | 2.85 | -1.88 | -1.17 | 0.68 | 0.25 |
| | Orb-D3-V2 | 0.19 | -0.12 | 2.53 | -1.74 | -0.83 | 0.57 | 0.34 |
| | Orb-D3-XS-V2 | -0.43 | 0.20 | 1.74 | -1.42 | -0.62 | 0.91 | 0.34 |
| | Orb-MPTrj-V2 | -0.68 | 0.73 | 1.62 | -1.51 | -0.30 | 0.53 | 0.47 |
| | Orb-V2 | -0.10 | 0.09 | 2.15 | -1.60 | -0.44 | 0.51 | 0.21 |

Continued on next page

**Table S11 – Continued from previous page.**

| Surface | Model | S1 | S2 | S3 | S4 | S5 | S6 | S7 |
|---|---|---|---|---|---|---|---|---|
| | Orb-V3-conservative-20-mpa | 0.61 | 0.05 | 2.72 | -1.54 | -1.33 | 0.33 | 0.09 |
| | Orb-V3-conservative-20-omat | 0.68 | 0.11 | 2.99 | -1.63 | -1.34 | 0.17 | -0.02 |
| | Orb-V3-conservative-inf-mpa | 0.32 | -0.06 | 2.56 | -1.70 | -0.98 | 0.48 | 0.38 |
| | Orb-V3-conservative-inf-omat | 0.11 | -0.05 | 2.49 | -1.53 | -0.88 | 0.46 | 0.32 |
| | Orb-V3-direct-20-mpa | 0.38 | 0.07 | 2.85 | -1.90 | -1.10 | 0.36 | 0.18 |
| | Orb-V3-direct-20-omat | -0.02 | 0.05 | 2.63 | -1.60 | -0.84 | 0.34 | 0.29 |
| | Orb-V3-direct-inf-mpa | 0.41 | 0.13 | 2.70 | -1.79 | -1.14 | 0.46 | 0.11 |
| | Orb-V3-direct-inf-omat | 0.24 | 0.20 | 2.74 | -1.64 | -1.07 | 0.23 | 0.10 |
| | PET-MAD-latest | -0.74 | -0.12 | 2.54 | -1.48 | -0.59 | 0.53 | 0.05 |
| | SevenN-0 | 0.09 | -0.32 | 2.91 | -1.26 | -1.47 | 0.34 | 0.42 |
| | SevenN-l3i5 | 0.08 | -0.33 | 2.91 | -1.26 | -1.47 | 0.34 | 0.42 |
| | SevenN-mf-ompa-mpa | 0.08 | -0.33 | 2.90 | -1.26 | -1.46 | 0.34 | 0.41 |
| | SevenN-omat24 | 0.08 | -0.32 | 2.91 | -1.26 | -1.46 | 0.34 | 0.41 |
| **Au(111)** | **DFT** | **0.08** | **-0.10** | **2.64** | **-1.32** | **-1.29** | **0.49** | **0.21** |
| | Equiformerv2-153M | 0.61 | -0.45 | 3.09 | -1.53 | -1.84 | 0.09 | 0.03 |
| | Equiformerv2-31M | 0.52 | -0.47 | 3.07 | -1.46 | -1.80 | 0.09 | 0.04 |
| | Equiformerv2-83M | 0.59 | 0.17 | 2.25 | -1.42 | -1.82 | 0.21 | 0.03 |
| | MACE-MATPES-R2SCAN | -0.21 | -0.27 | 2.50 | -1.17 | -1.64 | 1.17 | 1.49 |
| | MACE-MP-0-large | 0.21 | -0.19 | 2.77 | -1.30 | -1.60 | 0.89 | 0.14 |
| | MACE-MP-0-large-b2 | -0.18 | -0.49 | 2.83 | -1.35 | -1.60 | 1.35 | 0.38 |
| | MACE-MP-0-medium | -0.03 | -0.16 | 2.71 | -1.04 | -1.79 | 0.91 | 0.22 |
| | MACE-MP-0-medium-b | -0.19 | -0.34 | 2.91 | -1.20 | -1.52 | 1.39 | 0.32 |
| | MACE-MP-0-medium-b2 | -0.40 | -0.49 | 2.72 | -1.00 | -1.43 | 1.29 | 0.29 |

Continued on next page

**Table S11 – Continued from previous page.**

| Surface | Model | S1 | S2 | S3 | S4 | S5 | S6 | S7 |
|---|---|---|---|---|---|---|---|---|
| | MACE-MP-0-medium-b3 | 0.11 | -0.44 | 2.29 | -1.18 | -1.38 | 1.71 | 0.28 |
| | MACE-MP-0-small | 0.05 | -0.16 | 1.58 | -0.47 | -1.39 | 1.04 | 0.30 |
| | MACE-MP-0-small-b | -2.41 | -0.53 | 2.72 | -1.08 | -1.44 | 1.82 | 0.23 |
| | MACE-MP-0-small-b2 | -0.56 | -0.37 | 2.75 | -1.09 | -1.33 | 1.11 | 0.44 |
| | MACE-MPA-0-medium | -0.14 | -0.30 | 2.72 | -1.13 | -1.49 | 0.85 | 0.47 |
| | MACE-OMat-medium | -0.16 | -0.60 | 2.87 | -1.15 | -1.58 | 1.27 | 0.60 |
| | Orb-D3-SM-V2 | 0.05 | -0.36 | 2.72 | -1.44 | -1.40 | 0.95 | 0.21 |
| | Orb-D3-V2 | 0.19 | -0.21 | 2.55 | -1.56 | -1.20 | 0.94 | 0.23 |
| | Orb-D3-XS-V2 | 0.05 | -0.06 | 1.93 | -0.94 | -1.42 | 1.09 | 0.06 |
| | Orb-MPTrj-V2 | -0.74 | 0.09 | 1.30 | -0.03 | -1.14 | 1.06 | 0.30 |
| | Orb-V2 | 0.00 | 0.07 | 2.54 | -1.14 | -1.55 | 0.68 | 0.22 |
| | Orb-V3-conservative-20-mpa | 0.54 | -0.01 | 3.07 | -1.37 | -1.93 | 0.47 | 0.15 |
| | Orb-V3-conservative-20-omat | 0.63 | 0.04 | 3.29 | -1.44 | -2.06 | 0.38 | 0.11 |
| | Orb-V3-conservative-inf-mpa | 0.25 | -0.05 | 2.86 | -1.17 | -1.76 | 0.57 | 0.29 |
| | Orb-V3-conservative-inf-omat | 0.42 | 0.10 | 3.23 | -1.42 | -1.84 | 0.35 | 0.07 |
| | Orb-V3-direct-20-mpa | 0.21 | -0.02 | 3.18 | -1.55 | -1.65 | 0.49 | 0.18 |
| | Orb-V3-direct-20-omat | 0.41 | -0.03 | 3.42 | -1.41 | -2.08 | 0.33 | 0.21 |
| | Orb-V3-direct-inf-mpa | 0.15 | 0.07 | 2.87 | -1.28 | -1.73 | 0.65 | 0.14 |
| | Orb-V3-direct-inf-omat | -0.24 | 0.17 | 2.84 | -1.02 | -1.59 | 0.41 | 0.23 |
| | PET-MAD-latest | -0.94 | -0.08 | 1.91 | -1.17 | -0.93 | 1.30 | 0.08 |
| | SevenN-0 | -0.03 | -0.37 | 3.19 | -1.35 | -1.69 | 0.47 | 0.48 |
| | SevenN-l3i5 | -0.03 | -0.37 | 3.19 | -1.35 | -1.70 | 0.46 | 0.48 |
| | SevenN-mf-ompa-mpa | -0.03 | -0.37 | 3.19 | -1.34 | -1.70 | 0.47 | 0.48 |

Continued on next page

**Table S11 – Continued from previous page.**

| Surface | Model | S1 | S2 | S3 | S4 | S5 | S6 | S7 |
|---|---|---|---|---|---|---|---|---|
| | SevenN-omat24 | -0.03 | -0.37 | 3.19 | -1.35 | -1.69 | 0.47 | 0.47 |
| **Ni(110)** | **DFT** | **-0.91** | **-0.61** | **-0.41** | **-0.37** | **0.38** | **2.01** | **0.62** |
| | Equiformerv2-153M | -0.76 | -0.64 | -0.44 | 0.07 | 0.02 | 1.43 | 0.32 |
| | Equiformerv2-31M | -0.79 | -0.66 | -0.45 | 0.09 | 0.05 | 1.44 | 0.32 |
| | Equiformerv2-83M | -0.86 | -0.12 | -1.03 | 0.08 | 0.11 | 1.51 | 0.32 |
| | MACE-MATPES-R2SCAN | -1.24 | -1.34 | -0.14 | -0.05 | -0.00 | 2.56 | 2.09 |
| | MACE-MP-0-large | -0.98 | -0.12 | 4.39 | -1.55 | -1.42 | 0.52 | 0.08 |
| | MACE-MP-0-large-b2 | -1.15 | -0.09 | 5.06 | -1.79 | -1.49 | 0.29 | 0.10 |
| | MACE-MP-0-medium | -1.28 | -0.05 | 4.89 | -1.58 | -1.77 | 0.58 | 0.03 |
| | MACE-MP-0-medium-b | -1.21 | -0.04 | 4.76 | -1.20 | -2.04 | 1.07 | 0.04 |
| | MACE-MP-0-medium-b2 | -0.98 | -0.15 | 3.25 | -1.48 | -0.88 | 1.15 | 0.08 |
| | MACE-MP-0-medium-b3 | -0.90 | -0.08 | 3.62 | -1.12 | -1.46 | 1.18 | 0.14 |
| | MACE-MP-0-small | -1.86 | -0.04 | 5.30 | -1.77 | -1.04 | 0.35 | 0.02 |
| | MACE-MP-0-small-b | -2.68 | -0.09 | 4.81 | -1.45 | -2.38 | 1.09 | 0.02 |
| | MACE-MP-0-small-b2 | -1.28 | -0.16 | 3.66 | -1.31 | -1.46 | 1.11 | 0.40 |
| | MACE-MPA-0-medium | -1.71 | -0.04 | 7.27 | -2.00 | -2.14 | -0.51 | 0.14 |
| | MACE-OMat-medium | -1.32 | 5.60 | 1.28 | -2.55 | -1.95 | 0.16 | 0.03 |
| | Orb-D3-SM-V2 | -2.64 | 113.89 | 27.92 | 24.28 | -17.38 | -66.37 | -78.97 |
| | Orb-D3-V2 | -0.10 | 128.01 | -25.73 | 10.01 | -15.31 | -26.13 | -69.82 |
| | Orb-D3-XS-V2 | 0.93 | 62.39 | 18.61 | -46.02 | -26.35 | -6.93 | -1.91 |
| | Orb-MPTrj-V2 | -1.15 | 0.09 | 1.73 | -0.72 | -0.55 | 1.24 | 0.20 |
| | Orb-V2 | 18.73 | 0.12 | 155.59 | -11.49 | -38.20 | -37.96 | -85.99 |
| | Orb-V3-conservative-20-mpa | -0.82 | 57.09 | -36.28 | 2.81 | 14.94 | -3.07 | -33.76 |

Continued on next page

**Table S11 – Continued from previous page.**

| Surface | Model | S1 | S2 | S3 | S4 | S5 | S6 | S7 |
|---|---|---|---|---|---|---|---|---|
| | Orb-V3-conservative-20-omat | -0.87 | 1.13 | 7.14 | -2.94 | -2.80 | -0.39 | -0.30 |
| | Orb-V3-conservative-inf-mpa | -0.91 | 0.02 | 6.10 | -1.80 | -2.57 | 0.07 | 0.07 |
| | Orb-V3-conservative-inf-omat | -0.85 | 14.67 | -4.12 | -1.62 | 0.46 | -3.30 | -4.30 |
| | Orb-V3-direct-20-mpa | -1.09 | 21.65 | -1.60 | 2.05 | -6.56 | -6.59 | -7.02 |
| | Orb-V3-direct-20-omat | -0.91 | 35.39 | -1.55 | -6.78 | -7.05 | -6.26 | -11.99 |
| | Orb-V3-direct-inf-mpa | -1.04 | 10.47 | -1.74 | -0.24 | -4.55 | -0.83 | -1.19 |
| | Orb-V3-direct-inf-omat | -0.86 | 0.06 | 6.62 | -0.73 | -4.64 | 0.37 | -0.02 |
| | PET-MAD-latest | -2.10 | -1.51 | 0.43 | 0.22 | 0.04 | 2.38 | 0.72 |
| | SevenN-0 | -1.25 | -0.00 | 6.11 | -1.72 | -2.46 | 0.01 | 0.01 |
| | SevenN-l3i5 | -1.25 | -0.00 | 6.11 | -1.72 | -2.46 | 0.01 | 0.01 |
| | SevenN-mf-ompa-mpa | -1.26 | -0.00 | 6.10 | -1.72 | -2.46 | 0.01 | 0.01 |
| | SevenN-omat24 | -1.25 | -0.00 | 6.11 | -1.72 | -2.46 | -0.00 | 0.01 |
| **Ni(111)** | **DFT** | **-1.25** | **-0.06** | **-1.38** | **0.30** | **0.52** | **2.15** | **0.43** |
| | Equiformerv2-153M | -0.96 | -0.44 | -1.17 | 0.90 | -0.23 | 1.80 | 0.10 |
| | Equiformerv2-31M | -0.98 | -0.48 | -1.10 | 0.89 | -0.22 | 1.79 | 0.11 |
| | Equiformerv2-83M | -0.97 | 1.01 | -2.51 | 0.88 | -0.24 | 1.69 | 0.13 |
| | MACE-MATPES-R2SCAN | -1.41 | -0.63 | -1.19 | 0.23 | 0.43 | 2.67 | 1.78 |
| | MACE-MP-0-large | -1.09 | -0.13 | 5.30 | -1.61 | -1.84 | 0.18 | 0.10 |
| | MACE-MP-0-large-b2 | -1.34 | -0.11 | 5.59 | -1.95 | -2.01 | 0.60 | 0.15 |
| | MACE-MP-0-medium | -1.40 | -0.05 | 5.08 | -1.47 | -2.13 | 0.76 | 0.02 |
| | MACE-MP-0-medium-b | -1.20 | -0.04 | 5.30 | -1.19 | -2.57 | 1.03 | 0.05 |
| | MACE-MP-0-medium-b2 | -0.94 | -0.15 | 3.43 | -1.35 | -1.33 | 1.22 | 0.10 |
| | MACE-MP-0-medium-b3 | -0.95 | -0.09 | 3.56 | -0.92 | -1.60 | 1.23 | 0.15 |

Continued on next page

**Table S11 – Continued from previous page.**

| Surface | Model | S1 | S2 | S3 | S4 | S5 | S6 | S7 |
|---|---|---|---|---|---|---|---|---|
| | MACE-MP-0-small | -1.66 | -0.06 | 6.54 | -1.90 | -2.27 | 0.27 | 0.04 |
| | MACE-MP-0-small-b | -2.80 | -0.07 | 4.95 | -1.43 | -2.69 | 1.32 | 0.03 |
| | MACE-MP-0-small-b2 | -1.36 | -0.17 | 4.02 | -1.44 | -1.43 | 1.14 | 0.20 |
| | MACE-MPA-0-medium | -1.81 | -0.06 | 7.54 | -1.91 | -2.14 | -0.79 | 0.16 |
| | MACE-OMat-medium | -1.53 | -0.04 | 6.90 | -2.45 | -1.84 | 0.18 | 0.03 |
| | Orb-D3-SM-V2 | -1.81 | 37.39 | 17.70 | 10.89 | -6.38 | 1.08 | -58.14 |
| | Orb-D3-V2 | 0.00 | 93.22 | -9.88 | -8.87 | 3.64 | -15.64 | -61.53 |
| | Orb-D3-XS-V2 | -0.72 | 49.95 | 35.18 | -0.76 | -1.85 | -30.68 | -50.40 |
| | Orb-MPTrj-V2 | -1.68 | 0.11 | -0.35 | 0.32 | 0.13 | 1.87 | 0.45 |
| | Orb-V2 | 0.39 | -0.02 | 117.32 | -4.45 | -2.16 | -46.48 | -63.78 |
| | Orb-V3-conservative-20-mpa | -1.15 | 34.78 | 7.19 | -14.29 | 17.07 | -9.60 | -33.08 |
| | Orb-V3-conservative-20-omat | -1.17 | 0.04 | 8.11 | -1.37 | -3.96 | 0.24 | -0.93 |
| | Orb-V3-conservative-inf-mpa | -1.19 | 0.03 | 6.14 | -1.65 | -2.43 | 0.02 | 0.08 |
| | Orb-V3-direct-20-mpa | -1.43 | 4.03 | 26.59 | -3.16 | -11.33 | -6.84 | -7.02 |
| | Orb-V3-direct-20-omat | -1.37 | 31.04 | 12.90 | -14.42 | -3.17 | -6.39 | -17.75 |
| | Orb-V3-direct-inf-mpa | -1.42 | 1.22 | 7.90 | -3.91 | -1.85 | 0.90 | -1.96 |
| | Orb-V3-direct-inf-omat | -1.39 | 0.36 | 6.10 | -2.52 | -1.15 | 0.75 | -1.35 |
| | PET-MAD-latest | -2.35 | -0.39 | -0.67 | 0.26 | 0.76 | 2.13 | 0.43 |
| | SevenN-0 | -1.37 | 0.00 | 6.12 | -1.66 | -2.40 | -0.01 | 0.00 |
| | SevenN-l3i5 | -1.37 | 0.00 | 6.12 | -1.66 | -2.40 | -0.01 | 0.01 |
| | SevenN-mf-ompa-mpa | -1.38 | 0.00 | 6.11 | -1.66 | -2.40 | 0.01 | 0.01 |
| | SevenN-omat24 | -1.36 | 0.01 | 6.11 | -1.67 | -2.41 | -0.00 | 0.00 |
| **Pd(111)** | **DFT** | **-1.44** | **-0.00** | **-0.77** | **0.25** | **0.16** | **2.36** | **0.18** |

Continued on next page

**Table S11 – Continued from previous page.**

| Surface | Model | S1 | S2 | S3 | S4 | S5 | S6 | S7 |
|---|---|---|---|---|---|---|---|---|
| | Equiformerv2-153M | -0.81 | -0.43 | 0.75 | -0.06 | -0.70 | 1.17 | 0.08 |
| | Equiformerv2-31M | -0.78 | -0.43 | 0.72 | -0.04 | -0.71 | 1.18 | 0.07 |
| | Equiformerv2-83M | -0.82 | 0.19 | 0.08 | -0.03 | -0.68 | 1.16 | 0.09 |
| | MACE-MATPES-R2SCAN | -1.55 | -0.31 | -0.27 | 0.17 | -0.66 | 2.82 | 1.68 |
| | MACE-MP-0-large | -1.36 | 0.33 | 0.15 | 0.02 | -0.52 | 1.94 | 0.36 |
| | MACE-MP-0-large-b2 | -1.16 | -0.18 | 1.75 | -0.84 | -0.94 | 2.01 | 0.29 |
| | MACE-MP-0-medium | -1.87 | -0.24 | 1.05 | 0.04 | -0.87 | 2.07 | 0.64 |
| | MACE-MP-0-medium-b | -1.85 | 0.20 | 0.62 | -0.04 | -0.12 | 2.28 | 0.28 |
| | MACE-MP-0-medium-b2 | -0.70 | 0.19 | 0.85 | -0.64 | -1.19 | 2.04 | 0.44 |
| | MACE-MP-0-medium-b3 | -2.05 | -0.94 | 0.73 | 0.10 | -0.52 | 2.99 | 1.06 |
| | MACE-MP-0-small | -1.90 | -0.69 | 0.97 | -0.38 | 0.03 | 2.18 | 0.75 |
| | MACE-MP-0-small-b | -4.07 | -0.49 | 0.23 | 0.17 | -0.62 | 3.27 | 0.83 |
| | MACE-MP-0-small-b2 | -1.57 | -0.31 | 1.92 | -0.42 | -0.70 | 1.63 | 0.42 |
| | MACE-MPA-0-medium | -1.56 | -0.43 | 0.62 | 0.15 | -0.91 | 2.28 | 0.85 |
| | MACE-OMat-medium | -1.53 | -0.55 | 0.05 | 0.02 | -0.69 | 3.12 | 0.84 |
| | Orb-D3-SM-V2 | -1.20 | -0.27 | 0.31 | -0.47 | -0.02 | 2.09 | 0.28 |
| | Orb-D3-V2 | -1.33 | -0.18 | 0.38 | -0.03 | -0.24 | 1.85 | 0.47 |
| | Orb-D3-XS-V2 | -0.78 | -0.08 | -0.29 | 0.34 | -0.74 | 2.28 | -0.02 |
| | Orb-MPTrj-V2 | -1.92 | 0.12 | -0.32 | 0.97 | -0.68 | 1.98 | 0.68 |
| | Orb-V2 | -1.04 | 0.01 | 0.14 | 0.07 | -0.80 | 2.01 | 0.41 |
| | Orb-V3-conservative-20-mpa | -0.91 | 0.10 | 0.14 | 0.18 | -0.95 | 1.89 | 0.46 |
| | Orb-V3-conservative-20-omat | -1.45 | 0.02 | -0.13 | 0.60 | -0.72 | 2.07 | 0.58 |
| | Orb-V3-conservative-inf-mpa | -1.12 | -0.08 | 0.12 | 0.16 | -0.83 | 2.16 | 0.59 |

Continued on next page

Table S11 – Continued from previous page.

| Surface | Model | S1 | S2 | S3 | S4 | S5 | S6 | S7 |
|---|---|---|---|---|---|---|---|---|
| | Orb-V3-conservative-inf-omat | -1.05 | 0.05 | 0.73 | 0.11 | -0.84 | 1.48 | 0.42 |
| | Orb-V3-direct-20-mpa | -1.20 | 0.06 | 0.73 | 0.09 | -0.88 | 1.69 | 0.34 |
| | Orb-V3-direct-20-omat | -0.67 | 0.09 | 1.30 | -0.60 | -0.78 | 1.41 | 0.11 |
| | Orb-V3-direct-inf-mpa | -1.11 | 0.01 | 0.16 | 0.18 | -0.82 | 2.13 | 0.33 |
| | Orb-V3-direct-inf-omat | -1.41 | -0.05 | 0.09 | 0.35 | -0.66 | 2.07 | 0.41 |
| | PET-MAD-latest | -2.31 | -0.24 | -0.18 | 0.14 | -0.20 | 2.51 | 0.46 |
| | SevenN-0 | -1.40 | -0.38 | 0.25 | -0.02 | -0.76 | 2.35 | 0.64 |
| | SevenN-l3i5 | -1.40 | -0.38 | 0.25 | -0.02 | -0.76 | 2.35 | 0.64 |
| | SevenN-mf-ompa-mpa | -1.41 | -0.38 | 0.24 | -0.01 | -0.76 | 2.36 | 0.64 |
| | SevenN-omat24 | -1.40 | -0.38 | 0.25 | -0.02 | -0.76 | 2.35 | 0.64 |

# References


(1) Mallikarjun Sharada, S.; Karlsson, R. K. B.; Maimaiti, Y.; Voss, J.; Bligaard, T. Adsorption on transition metal surfaces: Transferability and accuracy of DFT using the ADS41 dataset. *Phys. Rev. B* **2019**, *100*, 035439.

(2) Araujo, R. B.; Rodrigues, G. L. S.; dos Santos, E. C.; Pettersson, L. G. M. Adsorption energies on transition metal surfaces: towards an accurate and balanced description. *Nature Communications* **2022**, *13*, 6853.

(3) Perdew, J. P.; Burke, K.; Ernzerhof, M. Generalized Gradient Approximation Made Simple. *Physical Review Letters* **1996**, *77*, 3865.

(4) Hammer, B.; Hansen, L. B.; Nørskov, J. K. Improved adsorption energetics within density-functional theory using revised Perdew-Burke-Ernzerhof functionals. *Phys. Rev. B* **1999**, *59*, 7413.

(5) Wellendorff, J.; Lundgaard, K. T.; Møgelhøj, A.; Petzold, V.; Landis, D. D.; Nørskov, J. K.; Bligaard, T.; Jacobsen, K. W. Density functionals for surface science: Exchange-correlation model development with Bayesian error estimation. *Phys. Rev. B* **2012**, *85*, 235149.

(6) González, J. M.; Sabadell-Rendón, A.; Kaźmierczak, K.; Euzenat, F.; Montroussier, N.; Curulla-Ferré, D.; López, N. Nickel Dynamics Switches the Selectivity of $CO_2$ Hydrogenation. *Angewandte Chemie International Edition* **2025**, *64*, e202417392.

(7) Kresse, G.; Furthmüller, J. Efficiency of Ab-Initio Total Energy Calculations for Metals and Semiconductors Using a Plane-Wave Basis Set. *Computational Materials Science* **1996**, *6*, 15.

(8) Blöchl, P. E. Projector Augmented-Wave Method. *Physical Review B* **1994**, *50*, 17953.

(9) Kresse, G.; Joubert, D. From ultrasoft pseudopotentials to the projector augmented-wave method. *Phys. Rev. B* **1999**, *59*, 1758.

(10) Lee, K.; Murray, É. D.; Kong, L.; Lundqvist, B. I.; Langreth, D. C. Higher-accuracy van der Waals density functional. *Phys. Rev. B* **2010**, *82*, 081101.

(11) Rajbhandari, S.; Rasley, J.; Ruwase, O.; He, Y. ZeRO: Memory Optimizations Toward Training Trillion Parameter Models, 2020, DOI: `10.48550/arXiv.1910.02054`.

(12) Geiger, M.; Smidt, T. e3nn: Euclidean Neural Networks, 2022, DOI: `10.48550/arXiv.2207.09453`.

(13) Chanussot, L. et al. Open Catalyst 2020 (OC20) Dataset and Community Challenges. *ACS Catalysis* **2021**, *11*, 6059.
